# InAsSb-Based Infrared Photodetectors: Thirty Years Later On

**DOI:** 10.3390/s20247047

**Published:** 2020-12-09

**Authors:** Antoni Rogalski, Piotr Martyniuk, Malgorzata Kopytko, Pawel Madejczyk, Sanjay Krishna

**Affiliations:** 1 Institute of Applied Physics, Military University of Technology, 00-908 Warsaw, Poland; antoni.rogalski@wat.edu.pl (A.R.); malgorzata.kopytko@wat.edu.pl (M.K.); pawel.madejczyk@wat.edu.pl (P.M.); 2 Department of Electrical Engineering, The Ohio State University, Columbus, OH 43210-1210, USA; krishna.53@osu.edu

**Keywords:** infrared detectors, InAsSb, superlattice, higher operating temperature

## Abstract

In 1989, one author of this paper (A.R.) published the very first review paper on InAsSb infrared detectors. During the last thirty years, many scientific breakthroughs and technological advances for InAsSb-based photodetectors have been made. Progress in advanced epitaxial methods contributed considerably to the InAsSb improvement. Current efforts are directed towards the photodetector’s cut-off wavelength extension beyond lattice-available and lattice-strained binary substrates. It is suspected that further improvement of metamorphic buffers for epitaxial layers will lead to lower-cost InAsSb-based focal plane arrays on large-area alternative substrates like GaAs and silicon. Most photodetector reports in the last decade are devoted to the heterostructure and barrier architectures operating in high operating temperature conditions. In the paper, at first InAsSb growth methods are briefly described. Next, the fundamental material properties are reviewed, stressing electrical and optical aspects limiting the photodetector performance. The last part of the paper highlights new ideas in design of InAsSb-based bulk and superlattice infrared detectors and focal plane arrays. Their performance is compared with the state-of-the-art infrared detector technologies.

## 1. Introduction

The development of InAs_1−x_Sb_x_ (InAsSb) has a long history. InAsSb was first synthesized in 1958 by Woolley and Smith [1]. This III–V material belongs to the family of narrow gap semiconductor compounds. Among them we can also distinguish IV–VI (Pb_1−x_Sn_x_Te), and II–VI (Hg_1−x_Cd_x_Te) infrared (IR) material systems. The bandgap of these semiconductors and hence the spectral response of the devices can be tuned for specific detectors’ applications. The favorable properties of narrow-gap semiconductors: high electron mobility, high optical absorption coefficient, and low thermal generation rate together with the capability for bandgap engineering, make these compounds almost perfect and ideal material for the design of detectors that are applied in a wide range of IR spectrum.

The most important position in IR detector technology is held by HgCdTe discovered in 1959 in the UK by Lawson, Neilsen, Putley, and Young [2]. HgCdTe has triggered the development an unprecedented degree of freedom in IR detector design and has inspired the development of the three “generations” of detectors considered mainly for military applications [3].

The first discoverers of HgCdTe also knew [4], and the explosions they experienced would have confirmed it, that the Hg vapor pressure over HgCdTe, which can be much greater than 20 atmospheres, would be a severe problem for crystal growth. The need to provide high mercury vapor pressure has been a major drawback during the growth of HgCdTe and for this reason alternative detector technologies were attempted over the past sixty years. One of those was PbSnTe, which was thoroughly studied in parallel with HgCdTe in the late 1960s and early 1970s [5,6,7]. Good quality long-wave infrared (LWIR) photodiodes were demonstrated as PbSnTe was comparatively easy to grow. However, in the late of 1970s there were two reasons which contributed to the termination of the research on PbSnTe: large temperature coefficient of expansion (TCE) mismatch with silicon and high dielectric constant. Relatively fast response time was required in scanned IR imaging systems of the 70s last century so that the scanned image is not blurry in the scan direction.

Nowadays with the trend towards focal-plane arrays (FPAs) this drawback does not play a key role like during the very first-generation systems’ design. In turn, due to large TCE, the indium bonds between silicon readout and the detector array in hybrid structure are exposed to adverse stresses during repeated cooling cycles from room temperature to cryogenic temperature. Today this drawback is omitted by fabrication of monolithic PbS and PbSe polycrystalline arrays on Si substrates.

The report on InAs_0.8_Sb_0.15_ photodiodes grown by step-graded liquid phase epitaxy (LPE) technique [8] initiated extensive research on InAsSb IR photodetectors in the mid 1970s. Near-lattice-matched InAs_1−x_Sb_x_ (0.09 ≤ *x* ≤ 0.15) device structures grown on GaSb substrates revealed the best parameters of InAs_1−x_Sb_x_ photodiodes [9]. InAsSb has the smallest bandgap of conventional III–V semiconductors and can be used as active material for mid-wavelength infrared (MWIR) and LWIR photodetectors. However, the energy gap of InAs_1−x_Sb_x_ during the 1970s was not exactly controlled in the middle composition range. In consequence, the InAsSb did not have a sufficiently small gap at lower temperatures, especially 77 K, for operation in the 8–14 μm wavelength range. Newly designed III–V strained-layer superlattices (SLs) consisting of a number of alternating thin mismatched crystal layers were developed to face this disadvantage.

Low dimensional solids representing new generation of materials for IR detectors have been proposed since the 1970s. Only a few years after the first GaAs/AlGaAs quantum heterostructures, the HgTe/CdTe SLs system was demonstrated in 1979 [10]. Two additional structures were subsequently introduced: InSb/InAsSb SLs with strain-induced bandgap reduction [11] and InAs/GaInSb SLs with superlattice-induced band inversion [12,13,14]. Even smaller bandgaps can be obtained than either constituent if InAs_1−x_Sb_x_ is combined with InSb or InAs in a SL. The first high-detectivity InAs_0.15_Sb_0.85_/InSb SLs photodiodes with detectivity ≥1 × 10^10^ cmHz^1/2^/W at wavelength, *λ* = 10 µm and 77 K were fabricated in 1990 by Kurtz et al. [15]. InSb/InAsSb SLs grown on InSb substrates revealed the excessive net stress, which inevitably caused cracks of the epitaxial material, making the fabrication of detector arrays impossible at that time [16].

The next step in development of SLs structures for IR detectors was concentrated on InAs/GaInSb material [12,13]. The larger valence band energy differences between InAs and GaSb than that between InSb and InAsSb directed mainstream research towards InAs/GaInSb type-II superlattices (T2SLs) allowing larger absorption in LWIR spectral range.

Historically, the InAs/InAsSb SLs as material for IR detectors had been selected more than 20 years after InSb/InAsSb ones [17]. Shorter Shockley-Read-Hall (SRH) minority carrier lifetimes of InAs/GaInSb T2SLs, were the main motivation to revive research into InAs/InAsSb T2SLs [18]. In addition, InAsSb-based detectors could be used in many civilian and military applications. The topic of InAsSb-based IR detectors has been covered only in two review papers [19,20] and one book [21]. In the last year, Steenbergen published an excellent chapter with the main emphasis on growth methods, material properties, and device fabrications [20]. In the present paper more attention is directed on the position of InAsSb-based detectors in confrontation with the present stage of IR detectors offered on global market. This evaluation is supported by theoretical estimates and experimental data.

## 2. Brief View on Crystal Growth of InAsSb-Based Materials

**Figure 1:**
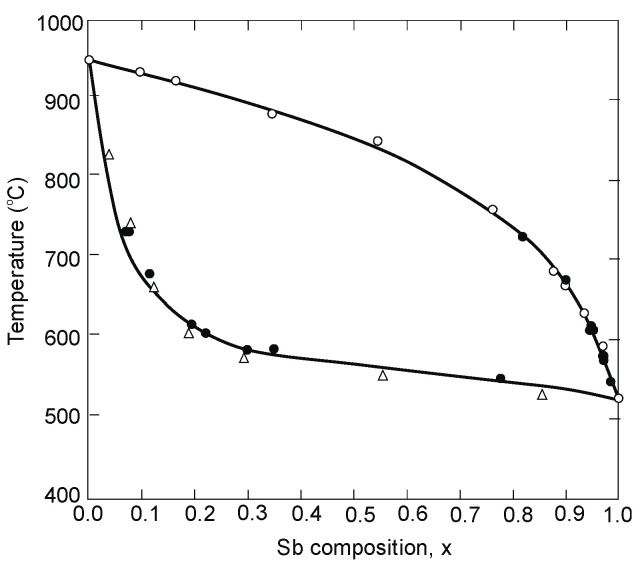
Pseudobinary phase diagram for the InAs-InSb systems [1].

The InAs_1−x_Sb_x_ properties were first investigated by Woolley and coworkers in the late 1950s. They determined InAs-InSb pseudobinary phase diagram [1], the miscibility [1], and identified the variation of properties such as bandgap [22,23] and effective masses on composition [24,25,26]. Polycrystalline InAsSb samples were measured to determine fundamental parameters. Many problems related to the InAsSb crystal growth arise from the pseudobinary phase diagram shown in Figure 1. The very low diffusion rates in the solid phase, the large separation between the liquidus and solidus, and the lattice mismatch (6.9% between InAs and InSb [a_o_(InAs) = 0.60584 nm, a_o_(InSb) = 0.64794 nm] are challenging for the crystal growth.

Progress in InAsSb ternary system has been limited by crystal synthesis problems. These difficulties are being overcome systematically by epitaxial methods such as: LPE, molecular beam epitaxy (MBE), and metalorganic chemical vapor deposition (MOCVD). Bulk single crystals are prepared mainly for compositions close to the binary compounds. A wide range of topics are gathered in thorough studies on electronic and photonic materials published in the *Springer Handbook of Electronic and Photonic Materials* [27].

LPE is a relatively simple high-quality technique, with less expensive epitaxial equipment and high utilization rate of the source material. It is particularly suitable for the preparation of thick-film layers. Since LPE technique is a near-thermodynamic equilibrium growth method, it cannot be used for the growth of the metastable ternary and quaternary antimonide compounds with miscibility gaps like InAsSb. The growth rate of LPE varies typically of 100 nm/min to a few μm/min (depending on substrate crystalline phases) and is generally higher than MBE and MOCVD. However, the controlled growth precision of very thin epilayers, especially quantum-well (QW) devices, SLs, and other complex microstructure materials cannot be implemented by LPE. In addition, the surface morphology of MBE or MOCVD grown layers is usually better than that grown by LPE.

The era of MBE and MOCVD growth of III–V semiconductors began in the early 1970s. It is often difficult to decide which epitaxial growth technique, MBE or MOCVD, to choose. Each of them has specific advantages in a given device application. Table 1 presents some characteristics related to the different classes of methods for III–V compounds [28].

MBE heterostructures are grown in ultra-high vacuum (UHV) chambers (base pressure ~10^−10^ Torr) on heated substrates typically by elemental sources. The elements are directed from high temperature effusion cells toward the substrate in the form of streams being chemically unchanged. The temperature of the substrate is independently controlled to facilitate layer-by-layer material incorporation to the substrate. The use of carrier gas is not necessary-the inherently long mean free paths result in highly directional elemental beams and the UHV environment ensures high material purity. Fast shutters enable deposition of more sophisticated layer structures like SLs and QWs. The valved sources are generally used because of relatively high vapor pressures for V group elements. Gas sources (e.g., CBr_4_ for C doping), precursor sources (e.g., GaTe for Te doping), and plasma sources (e.g., nitrogen plasma) are applied.

MBE enables the control of the composition of the growing crystal with monolayer (ML) resolution due to the both slow growth rate (~1.0 ML per second) and mounting the shutters just in front of the crucibles. Calibration of the growing crystal with the electron beams is possible because of low background pressure in MBE. The layer-by-layer growth mode, III/V flux ratio, as well as the quality of the growing substrate can be determined by the reflection high energy electron diffraction (RHEED) technique. MBE provides the ability to control the interfaces by shutter sequence with a precision of 0.1 s because the growth proceeds in thermodynamic imbalance conditions. The leaks can be a major problem because the growth proceeds under extremely low pressure. The growth chamber is predominantly cooled by liquid nitrogen to strengthen high vacuum by preventing molecules from peeling off from the chamber walls.

MOCVD is another important growth technique widely used for heteroepitaxy of the QW structures and SL. Similarly to MBE, it also enables the growth of heterostructures with abrupt interfaces between the individual layers. The growth in MOCVD technique proceeds on a heated substrate but in a much higher pressure than MBE (typically 15 to 750 Torr). There are several types of MOCVD reactor designs. The growth proceeds at near atmospheric pressure in the atmospheric MOCVD and this requires the use of a large amount of carrier gases. In turn, the reactor pressure is kept low in the low pressure MOCVD and in this case the growth rate is then slower as in the MBE. Strict security precautions must be incorporated in the MOCVD laboratory. In order to avoid any deadly accidents, many safety precautions have to be implemented because the precursor gases and the carrier gases are often highly toxic or explosive. In almost all semiconductor fabrications, safety and environmental concerns are important issues because the growth processes are almost always associated with toxic and dangerous materials. 

MOCVD growth proceeds not by physical deposition but by chemical reaction. In contrast to MBE, MOCVD technique requires more complex compound precursors, namely metal-organic sources (e.g., di-methyl or tri-ethyl Cd, Te, Al, etc.), hydrides (e.g., PH_3_, etc.), and other gas sources (e.g., Si_2_H_6_). The partial pressures of the precursors are controlled with mass flow controllers. In MOCVD, the precursors are transported over the substrate where they pyrolyze resulting in epitaxial growth. MOCVD requires the use of a carrier gas (typically hydrogen) to transport the precursors across the wafer surface. The valve actuation for varying injection ports of a gas manifold enables the growth of complex heterostructures. The fabrication of optoelectronics devices involving thermodynamically metastable alloys is dominated by both MBE and MOCVD techniques.

From the economical point of view (see Table 1 and [28]):

- the cost characteristics are very different because of the specific requirements:
  - for MBE the overhead is relatively fixed and does not vary with volume,
  - for MOCVD, the overhead costs tend to vary with production volume, and therefore
- MOCVD exceeds in a case of significant overcapacity (long idle time), and the opposite is true for MBE which wins on a cost basis when fully loaded,
- the two methods are very similar considering the production efficiency time,
  - for MBE, the idle time lasts on average several months where the reactor is down,
  - MOCVD service procedures are much more frequent but less time consuming, and therefore
- for MBE system, significant bake times are needed when the growth chamber must be opened for the service repairs,
- in contrast, MOCVD does not require long bake times (MOCVD is able to recover more quickly from failures).

Manasevit and Simpson firstly performed epitaxial growth of antimonides thin layers using MOCVD with TMGa (trimethylgallium) and SbH_3_ (stibine) precursors for GaSb films deposition in 1969 [29]. At present, the typically used III-group metal-organic sources by MOCVD for antimonide-based materials are 3-methyl compound and 3-ethyl compound, such as: TMAl, TMIn, TMGa, TEIn, TEGa, etc. [30,31]. In turn, V-group commonly used precursors are: AsH_3_, PH_3_, TMBi, TMSb, and RF-N_2_, etc.

The growth temperature of antimonides (low melting point materials) is close to 500 °C. It appears that below 500 °C the vast majority of III-group metal-organic precursors cannot decompose in 100%. Therefore, new organic source materials with a lower decomposition temperature are introduced including: TDMASb (trisdimethylaminoantimony), TASb (triallyantimony), TMAA (trimethylamine alane), TTBAl (tritertiarybutylaluminum), EDMAA (ethyldimethylaminealane), etc. In the case of Al-containing antimonide materials, carbon and oxygen contamination problems exist. The active hydrogen atoms’ absence on the surface of epitaxial layers is expected to be the reason of this effect. Carbon is typically p-type doping impurity, which causes certain difficulties in growing of n-type doping Al-containing antimonide epitaxial layers. 

MBE grown antimonides was first reported in the late 1970s [32,33]. In comparison with GaAs and other arsenides, the growth of GaSb-based is characterized by relatively low Sb vapor pressures, or, equivalently, by its high sublimation energy. GaSb and AlSb are nearly lattice-matched mutually and to InAs and for this reason they are the subject of intensive research. The substrate temperature during the growth of GaSb and AlSb is usually between 550 and 600 °C. MBE reduces the concentration of O-doping and avoids the C-contamination issue in Al-based materials growing by MOCVD. Most of low-dimensional structures (quantum wires-QRs, QWs, and quantum dots-QDs) and devices having complex structures were first grown by MBE. It was experimentally proven that the implementation of crystallographically misoriented substrates (small angle offset) contributed to higher-quality epitaxial layers [34].

The growth of antimonide-based III–V epitaxial layers is usually performed on InSb, InAs, and GaSb low-defect substrates. GaAs and GaAs-coated Si substrates and other heterogeneous substrate materials for epitaxy were introduced to face the problem that antimonides have no semi-insulating substrate. The growth of multilayers with abrupt but incremental compositional shifts between adjacent layers and continuous compositional grading of thick epilayers enables one to obtain a variety of substrate structures. Wafer bonding techniques and selective removal of the seeding substrate methods can be combined in IR detector fabrications. With high-quality substrates with increased functionality and bandgaps and lattice constants differing significantly more than feasible with binary compound wafers (e.g., GaSb or InAs), it seems possible to implement [35].

The initial efforts in fabrication and characterization of InAsSb material until 1994 were described in two Rogalski papers [19,21]. The book chapter written recently by Steenbergen [20] has provided updated information on the growth methods and characterization techniques of InAsSb bulk and SLs materials for the last thirty years. Because of this, here only main trends in material fabrication are covered.

Current interest is directed toward the InAsSb photodetectors cut-off wavelength (*λ_cut-off_*) extension beyond that available when lattice-matched or lattice-strained to binary substrates; mainly GaSb. In order to improve FPAs manufacturability and increase their size, low-cost GaAs and Si substrates are used. The Si substrates are very convenient in IR FPA technology because the coupling with Si readout enables fabrication of very large arrays exhibiting the prolonged thermal cycle reliability (Si substrates are cheaper and available in large area).

In practical design of InAsSb-based device structures, a serious problem is the control of photodetector’s *λ_cut-off_*. The accuracy of the bandgap depends on material composition, stress in the buffer layer between substrate and the active region, the crystalline quality of the ternary samples, (potentially CuPt ordering) which is determined by the growth method and conditions, and finally the bandgap measurement uncertainty. Different techniques used in bandgap characterization (absorption, ellipsometry, photoluminescence (PL) and electroluminescence (EL)) and experimental results are given by Steenbergen [20].

**Figure 2:**
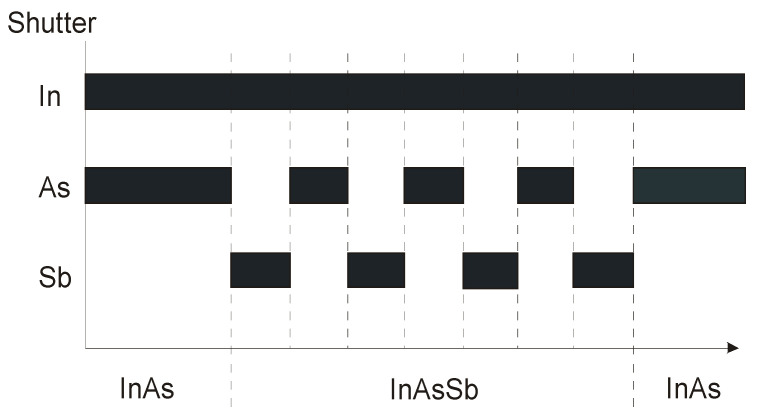
The scheme of the MMBE shutter sequence during the growth of InAs_0.76_Sb_0.24_ and the surrounding InAs layers. The filled bars denote intervals when the corresponding shutters are open (after [37]).

Usually, the spontaneous ordering of CuPt in mid-composition range of InAs_1−x_Sb_x_ causes a problem. Better alloy composition uniformity of group V materials on lattice-mismatched substrates is required to grow controlled, high crystalline quality InAsSb without spontaneous ordering [36]. In the technology of ternary semiconductors, atoms of group V having different sticking coefficients are more difficult to control than group III ternaries [37,38]. Using modulated MBE (MMBE) technique with arsenic and antimony shutter modulation (see Figure 2), better control of InAsSb composition is reached.

**Figure 3:**
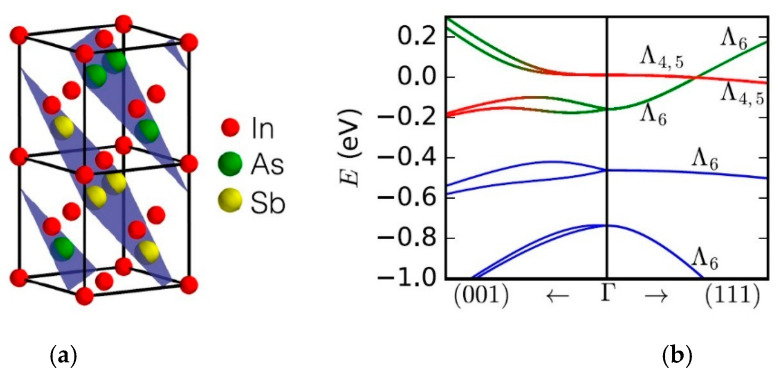
CuPt-ordered InAs_0.50_Sb_0.50_: (**a**) crystal structure with the alternating Sb-As (111) planes highlighted, (**b**) empirical tight binding band structure calculation around Γ for |**k**| ≤ 0.1 Å^−1^ (after [40]).

The spontaneous CuPt ordering during InAsSb MBE growth is probably caused by residual strains [39]. Figure 3 presents the crystal structure of CuPt-ordered InAs_0.50_Sb_0.50_ and the calculated band structure indicating inversion of the Λ_4,5_ and Λ_6_ bands, pointing the semimetallic character.

The quality of InAsSb has improved greatly in the last decade. Significant progress has been made to advance metamorphic buffer layers for LWIR MBE photodetector structures. Different compositionally graded buffer schemes have been studied with the absorber composition *x* ≈ 0.60, including GaInSb and AlGaInSb graded buffer layers on a GaSb substrate to extend detector’s *λ_cut-off_* [41,42].

In comparison with MBE, MOCVD also allows to grow epitaxial structures on large-area wafers providing higher production throughput (MOCVD is more economical and characterized by less problematical maintenance). However, a disadvantage of this technique is less sharp interfaces, especially in SLs fabrication. More details about InAsSb structures MOCVD growth are included in [20].

## 3. InAsSb Alloy Properties

Table 2 shows selected properties of semiconductors, along with narrow-gap alloys, used in IR photodetectors manufacturing characterized by zincblende (ZB) or diamond (D) crystal structure. There is a tendency in transition of chemical bond from the covalent group IV-semiconductors to more ionic II–VI materials with the lattice constant increasing when moving across the table from the left to the right. The materials become softer and the chemical bonds become more delicate which results in the quality of the bulk. The semiconductors with a greater share of covalent bonds are more mechanically stable, which contributes to better fabrication. The supreme position of GaAs in optoelectronics and silicon in electronic materials supports this observation. In turn, the energy gap of semiconductors on the right side of the table shows a tendency to have lower values. Higher band-to-band (BtB) absorption provides increased quantum efficiency (*QE, η*) observed for example in HgCdTe or InSb because of their direct bandgap structure.

A high density of states in the valence band (VB) and conduction band (CB) leading to enhanced absorption of IR radiation and a comparatively low thermal generation rate arise from the direct energy bandgap structure of narrow gap semiconductors applied in the IR detectors. The III–V semiconductors are characterized by much stronger chemical bonds and therefore higher chemical stability compared to HgCdTe, which is important from the producibility viewpoint.

### 3.1. Energy Gap

The III–V semiconductors are characterized by a ZB structure and direct energy gap at the Brillouin zone centre. The **k** · **p** theory explains the shape of the electron and the light mass hole bands. For different semiconductors, the momentum matrix element changes slightly and is typically about 9.0 × 10^−8^ eVcm. Therefore, the CB densities of states and the electron effective masses have comparable values for materials with the same energy gap. 

The Varshni formula clearly describes a conventional negative temperature coefficient of the energy gap of these materials [43]:

(1) $$E_{g}\left(T\right)=E_{o}-\frac{\alpha T^{2}}{T+\beta },$$

where *E_o_* is the bandgap at 0 K, *T* is the temperature, *α* and *β* are fitting parameters for a given material. *β* is closely related to the material’s Debye temperature (in Kelvin). 

If *T* >> *β*, then Equation (1) becomes ∂*E_g_*(*T*)/*∂T ~−α.* Viña et al. [44] proposed a new expression for temperature dependence of the bandgap energy by taking into account the Bose–Einstein occupation factor [45]

(2) $$E_{g}\left(T\right)=E_{o}-\alpha_{B}\left(1+\frac{2}{exp\left(\Theta /T\right)-1}\right),$$

where the parameter Θ describes the mean frequency of the phonons involved and *α_B_* is the strength of the electron-phonon interaction. While Varshni expression is entirely empirical, the Bose–Einstein retains physical meaning and is more palatable from the theoretical point of view. It is assumed that the Varshni parameter *β* is comparable to the Debye temperature, *Θ* = *<E_p_>*/*k*, thus the Bose–Einstein average phonon energy can be used to calculate the Debye temperature. If *kT* >> <*E_p_*>, the Varshni and Bose–Einstein expressions are equivalent. For lower temperatures in which *kT* < *<E_p_>*, the Bose–Einstein formula usually provides a better fit to the experimental results (Ref. [20] discusses in detail the bandgap temperature-dependent parameters).

The dependence *E_g_*(*x*,*T*) for InAs_1−x_Sb_x_ has been experimentally investigated by many research groups since 1964 [22]. The first investigation of the polycrystalline samples optical properties indicated on the nonlinearity of the InAs_1−x_Sb_x_ bandgap composition dependence, which can be represented by the bowing parameter *C_g_* as

(3) $$E_{g}\left(x\right)=E_{gInSb}x+E_{gInAs\left(1-x\right)}-C_{g}x\left(1-x\right).$$

**Figure 4:**
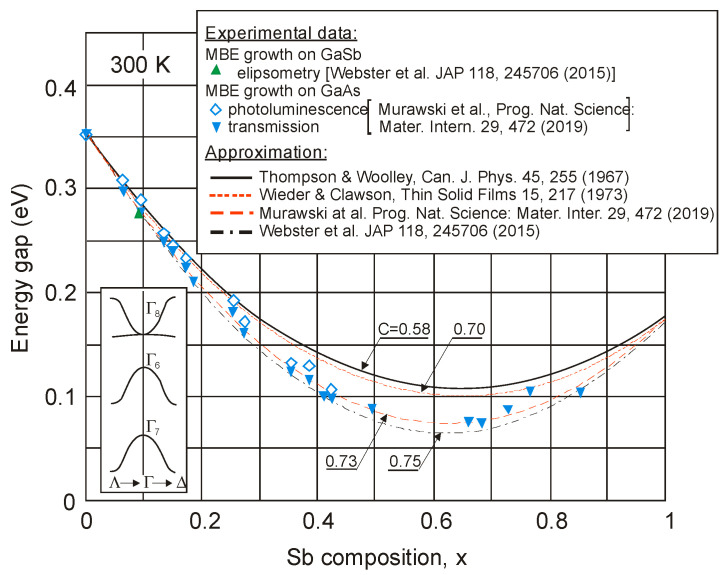
InAs_1−x_Sb_x_ bandgap energy versus the Sb composition at room temperature. The experimental data is taken with different papers [46,47,48,49] as indicated in the legend.

**Figure 5:**
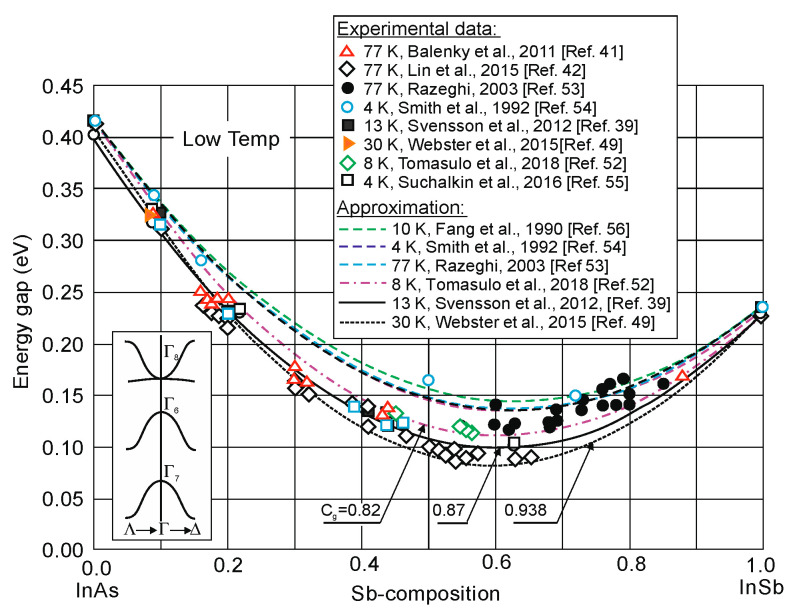
InAs_1−x_Sb_x_ bandgap energy versus the Sb composition at low temperatures. The experimental data are taken with different papers as indicated in the key [39,41,42,49,52,53,54,55,56].

Ten different relations *E_g_*(*x*,*T*) obtained from measurements over different *x*-composition values for a wide range of temperatures are presented in [20]. The low-temperature (4–13 K) *λ_cut-off_* estimated from *λ_cut-off_* [μm] = 1.24/*E_g_*[eV] changes from 8.4 μm to 12.5 μm. The smallest bandgap of InAs_1-*x*_Sb*_x_* occurs for *x* ≈ 0.60–0.64; however, for such composition lattice-matched binary substrate has not been developed yet. The bandgap expressions from many papers are plotted in Figure 4 and Figure 5 for both high and low temperatures.

At present, *C_g_* value close to 0.7 eV is correct in higher temperature range (Figure 4). The evaluated *E_g_*(*x,T*) dependence differs from that previously described by Wieder and Clawson in 1973 [46]:

(4) $$\begin{array}{c} E_{g}\left(x,T\right)=0.411-\frac{3.4\;\times \;10^{-4}T^{2}}{210+T}-0.876x+0.70x^{2}\\ +3.4\times \;10^{-4}xT\left(1-x\right). \end{array}$$

Initial reports based on experimental data at temperatures above or near 100 K estimate the direct-gap bowing parameter in InAsSb at 0.58–0.6 eV [50]. According to Rogalski and Jóźwikowski’s theoretical considerations, the bowing parameter should tend to move to a higher value of about 0.7 eV [51]. 

The experimental data from VIGO System collected in recently published paper [47] fits well with a parabola having *C* = 0.72 eV and relation

(5) $$\begin{array}{c} E_{g}\left(x,T\right)=0.417-1.28\;\times \;10^{-4}T-2.6\;\times \;10^{-7}T^{2}\\ -x\left(C_{g}+0.182+10^{-9}T^{2}\right)+C_{g}x^{2}. \end{array}$$

This relation is recommended for InAsSb layers grown on GaAs lattice-mismatched substrates and is valid for both low and high temperatures.

More recent PL studies on unrelaxed MBE-grown InAs_1−x_Sb_x_ in wide range of composition gave the bowing parameter from 0.83 to 0.938 eV [39,42,49,52]. Until now, the highest bandgap bowing parameter 0.938 eV for low temperature has been reported by Webster et al. [49]. Such large parameter results in the considerable reduction in the smallest bandgap, ≈25–75 meV, at low temperature. At room temperature, Webster et al. [49] have obtained much lower bowing parameter of 0.75 eV and developed the temperature-dependent bandgap bowing model. The literature data for InAsSb layers grown by MBE on GaSb fits well with this model end equation

(6) $$\begin{array}{c} E_{g}\left(x,T\right)=0.417-1.28\;\times \;10^{-4}T-2.6\;\times \;10^{-7}T^{2}-x\left(C_{g}+0.182+10^{-9}T^{2}\right)+\\ +x^{2}\left(C_{g}-5.8\;\times \;10^{-4}+10^{-7}T^{2}\right). \end{array}$$

Figure 5 summarizes the experimental data and theoretical predictions of energy gap in temperature range 4–77 K published in different papers. Discrepancies in the *E_g_*(*x,T*)-dependence may result from several factors among others: strains, structural quality of samples, and CuPt-type ordering effect.

The low energy gap data reported earlier were masked probably by electron filling of the CB, which resulted from background doping due to various degrees of residual strain and relaxation in these samples. As is marked in Section 2, high quality of unstrained and unrelaxed InAsSb epilayers have been developed by using special graded buffer layers which reduces the substantial difference between the lattice constant of the alloy and the substrate. Electron diffraction patterns of unstrained InAsSb alloys, described in [41,42], showed an ordering-free distribution of group V elements, which indicates that the observed energy gaps of ternary alloys are inherent (both ordering and residual strain effects were eliminated).

**Figure 6:**
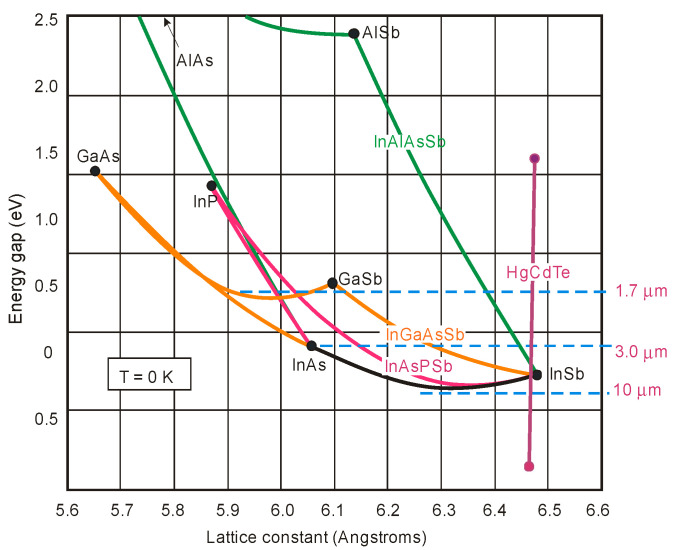
Composition and wavelength diagram of semiconductor material systems.

The above discussion indicates that the InAsSb bandgap energy is approximately a square function of the composition and shows a weak dependence of the band edge on composition in comparison with HgCdTe (see Figure 6).

### 3.2. Bandgap Alignment and Offsets

**Figure 7:**
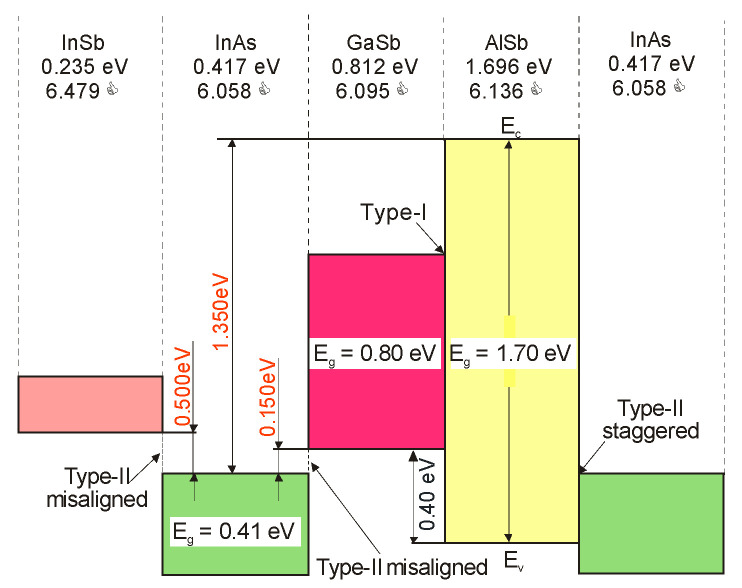
Schematic diagram of the low-temperature energy band alignment in the nearly 6.1 Å lattice matched InAs/GaSb/AlSb compounds. In this material system three types of band alignment are possible: type-I (nested) band alignment between GaSb and AlSb, type-II staggered alignment between InAs and AlSb, and type-II misaligned (or broken gap) alignment between InAs and GaSb. The approximate values of band offsets are marked in red.

The SL photodetector’s design process requires the bandgap alignment and offsets for InAsSb in relation to InAs, InSb, and other III–V semiconductor alloys. As is shown in Figure 6, these materials belong to 6.1 Å III–V semiconductor family. They are the most important in proposing new solutions of high-performance IR detectors exhibiting direct energy gaps, high optical absorption, and high design flexibility. This group consists of three alloys having approximately matched lattice constant about 6.1 Å: InAs, GaSb, and AlSb. Their energy gaps are varying in the range starting from 0.417 eV (InAs) to 1.696 eV (AlSb) at lower temperatures [57]. Similarly to other semiconductor compounds, they are chosen as the subject of research due to their heterostructures, in particular combining InAs with the three antimonides (InSb, AlSb, and GaSb) and their alloys. This combination provides a band alignment that is fundamentally different from that of the more thoroughly researched AlGaAs and that band alignment flexibility draws the attention of scientists on the 6.1 Å materials. InAs/GaSb heterojunctions reveal the most unique band alignment, which is defined as the broken gap. The top of the VB of GaSb is located above the bottom of CB of InAs by ~150 meV at the interface. On both sides of the heterointerface, electrons and holes are separated and located in self-consistent QWs, which is related to partial overlapping of the InAs CB with the GaSb VB. Consequently, exceptional tunneling-assisted radiative recombination transitions and novel transport properties are observed. A wide range of different alloys and SLs can be designed due to high versatility of these compounds, with the accessibility of type-I (nested, or straddling), type-II staggered, and type-II broken gap (misaligned) band offsets between the GaSb/AlSb, InAs/AlSb, and InAs/GaSb material pairs, respectively, as illustrated in Figure 7.

**Figure 8:**
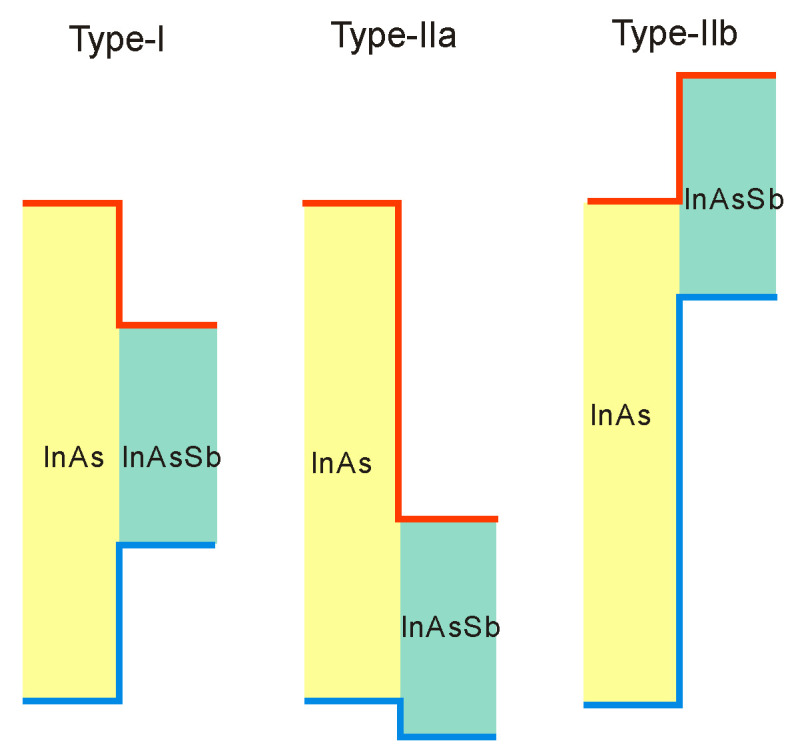
Three possible band alignments between InAs and InAs_1−x_Sb_x_.

Due to different growth conditions causing the variety of residual strain in the materials and due to the discrepancies related to the bandgap measurement methods, the band offsets are historically not easy to estimate. Steenbergen et al. [58] have reviewed the band edge alignment models for InAs-InAs_1−x_Sb_x_ system and considered different types of heterojunctions. This review is updated in recently published monograph [20]. Figure 8 shows three possible band alignments between InAs and InAs_1−x_Sb_x_ including two type-II band alignments: with the InAs CB higher in energy than the InAsSb CB and with the InAsSb CB higher in energy than the InAs CB. From these two papers [20,58], the following conclusions could be drawn:

- the type-I alignment is notified for SLs with low Sb compositions and ordering typical for the InAs1-xSbx layer, which is characterized by a lower alloy bandgap than that of a random alloy with equal composition x [59],
- the type-IIa alignment is based on argued conclusions for the reduced mass values of the lowest two transitions given by PL and magneto-transmission measurement results [60,61],
- type-IIb, is chosen for InAs/InAs0.93Sb0.07 SLs on InAs substrates [62].

**Figure 9:**
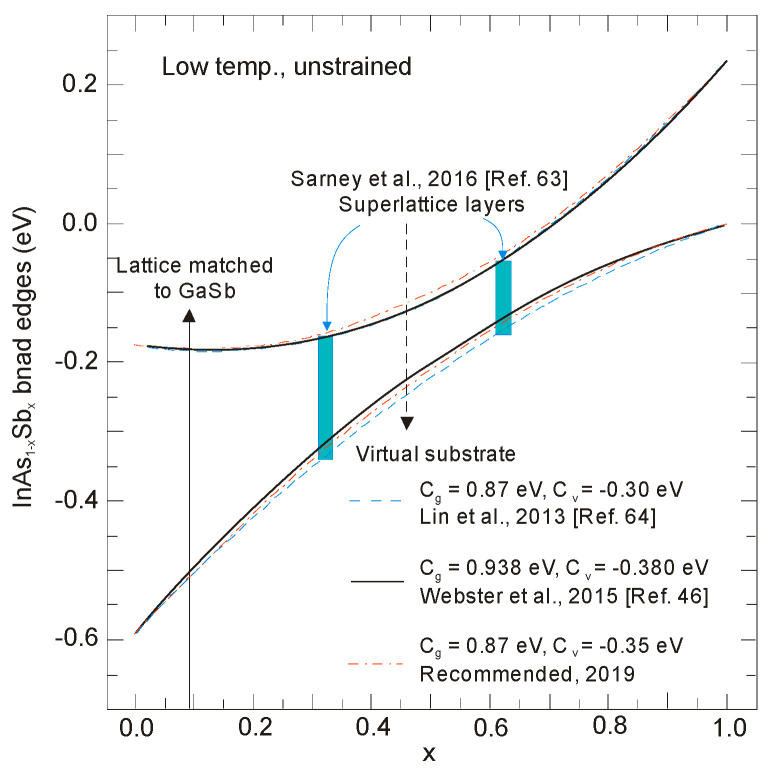
InAs_1−x_Sb_x_ VB and CB edges and their alignment assuming that *x* = 0 for InAs and *x* = 1 for InSb. As noted by the dashed arrow, longer wavelengths than InAsSb can be reached with T2SLs grown on a virtual substrate InA_0.54_Sb_0.46_ (after [20,63]). Bandgap bowing, *C_g_*, has been distributed to the VB, *C_v_*, and CB as shown according to the references [49,64]. The dash-dot curves represent the suggested bandgap and VB bowing equal 0.87 and −0.35 eV, respectively. The solid arrow points the composition of InA_0.91_Sb_0.09_ lattice-matched to GaSb substrates.

Investigations carried out during the last several years have revealed the InAsSb band edges versus composition dependence as shown in Figure 9 [20]. The recommended parameters are as follows: InAs/InSb valence band offset (VBO), −0.59 eV; the InAsSb bandgap bowing, 0.87 eV; and the VB bowing, –0.35 eV. Moreover, a concept of the band offsets between InAs and InAsSb with slight dependence on temperature was introduced. It follows that the bandgaps are the main temperature-dependent factors with the band offsets and VB bowing being distributed by the same fractions in the whole temperature range [49].

The InAsSb properties are direct consequence of the energy band structure. Table 3 contains some material parameters of the InAs, InSb, GaSb, and InAs_0.35_Sb_0.65_ semiconductors [3,65]. Between them, InSb has been investigated most broadly.

### 3.3. Transport Properties

Virtual crystal approximation (VCA) describes the electronic properties of ternary alloys according to the approach [66], where the disordered alloy is modeled by an ideal crystal with an average potential at each sublattice site. Linear interpolation of the potentials of corresponding binary semiconductors is realized by the potential averaging. The nonlinearity of composition dependence versus the energy gap (see Equation (3)) is calculated based on VCA model because of the nonlinear relationship of the band energies against the crystal potential. In order to explain the large bandgap bowing, the VCA has to be modified by adding the random potential due to fluctuations of the alloy composition [67,68]. Presently, it is commonly assumed that the material disorder influences not only the large bandgap bowing in the InAsSb alloy but also shapes the composition dependence on fundamental parameters such as mobilities or carrier effective masses.

**Figure 10:**
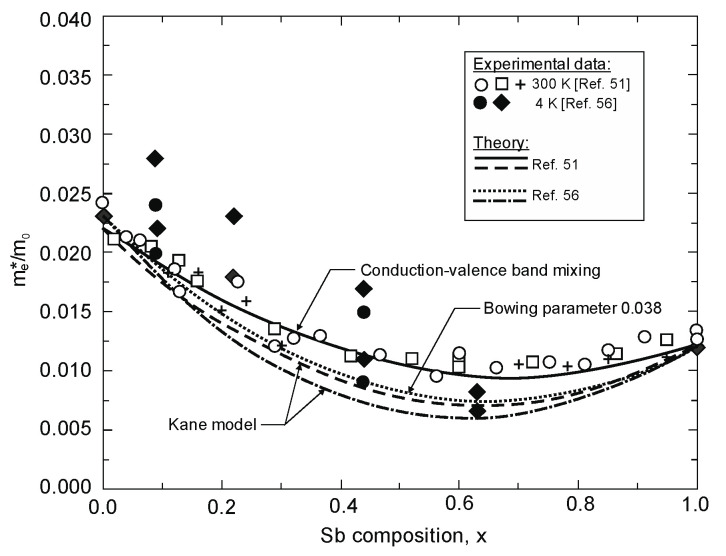
Effective mass of electron versus composition for InAs_1−x_Sb_x_. The experimental data is taken from [51,56].

The conduction-valence-band mixing theory [23] has been implemented to obtain a good agreement between experimental room temperature effective masses and calculations, as reported by Rogalski and Jóźwikowski [51] (see Figure 10). More recently published low-temperature data, especially for mid-composition range, indicates lower electron effective masses. Estimated negative bowing parameter for the electron effective mass is *C_m_* = 0.038 and is slightly less than expected from the Kane model (*C_m_* = 0.045), reaching the lowest effective mass (0.0082*m*_0_ at *x* = 0.63 and 4 K) ever reported for III–V semiconductors. A possible reason for the effective mass value discrepancy, shown in Figure 10, is the alloy disorder causing the random potential distribution which disturbs the CB and VB states.

**Figure 11:**
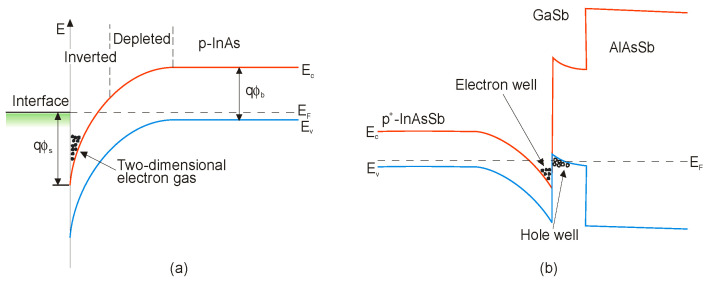
Band edges of p-type InAs and InAsSb with surface electron accumulation: (**a**) InAs with surface inversion layer; the electrostatic potential has the value *Φ_s_* at surface and *Φ_b_* in the bulk, (**b**) InAsSb/AlAsSb heterojunction with depicting surface electrons, epilayer, and interface with AlAsSb.

The electrical properties of semiconductor compounds and their dependence on composition are very important for device design with the optimal performance. However, it is difficult to control carrier concentration because insulating or semi-insulating substrates for InAsSb epitaxial layers are generally not available. Parallel conduction layers such as the substrate and/or the interface with the substrate presence make Hall effect measurements difficult to interpret. Moreover, it has been well known from the beginning of the 1970s [69,70] that narrow gap III–V semiconductors, especially InAs, creates surface electron accumulation layers. For example, Figure 11 presents band edges of p-type InAs [69] and InAsSb [71] with surface electron accumulation. There is strong electron accumulation on the InAsSb side of InAsSb/GaSb heterojunction due to the staggered type II band alignment. As is shown in Figure 11b, the surface states cause the Femi level to be pinned above the CB creating a parallel conduction path to the epilayer. In this case, the accurate determination of the carrier concentration and mobility with standard Hall measurements is challenging. 

The above-mentioned effect should be taken into account to analyze electrical carrier concentration and mobility measurements. A popular two-layer model has been often used to separate the surface accumulation and epilayer properties. Another approach is applied in the case of more complicated structures like that shown in Figure 11b. Mobility spectrum analysis and/or variable temperature and magnetic field-dependent transport analysis with multicarrier fitting models have been applied to segregate the epilayer and surface and interface layer characteristics [71].

The variation of InAs_1–*x*_Sb*_x_* electrical properties depend on the composition. Similarly to binary compounds, ternary alloys also have the surface accumulation layers and interface layers with the buffers or substrates. Special attention is required in estimates of carrier concentrations in selected device regions. More information on that topic can be found in [20].

As a popular donor dopant in InAs, silicon is used. Beryllium and zinc have been used for InAs and InSb photodiode fabrications for conversion of n-type materials to p-type ones [3]. Silicon behavior in InSb is amphoteric; in pulled crystals results in p-type conductivity for temperatures >400 °C, however for MBE growth epilayers results in n-type when the growth temperature is >350 °C, but for growth temperatures <320 °C gives donor conductivity [20]. Germanium is used as an acceptor dopant in InSb but behaves as a donor in InAs for LPE-grown epilayers [72].

Rogalski and Jóźwikowski have calculated the InAs_1−x_Sb_x_ intrinsic carrier concentration for the conditions 0 ≤ *x* ≤ 1 and 50 ≤ *T* ≤ 300 K. In order to obtain a good agreement between experimental values and calculations, the conduction-valence-band mixing was taken into account [51]:

(7) $$\begin{array}{c} n_{i}=\left(1.35+8.50x+4.22\;\times \;10^{-3}T-1.53\;\times \;10^{-3}xT-6.73x^{2}\right)\;\times \\ 10^{14}T^{3/2}E_{g}^{3/4}exp\left(-\frac{E_{g}}{2kT}\right), \end{array}$$

where *n_i_* is in cm^−3^ and *k* is the Boltzmann constant. The maximum of *n_i_* occurs at *x* ≈ 0.63 which corresponds to the minimum energy gap for a given temperature.

**Figure 12:**
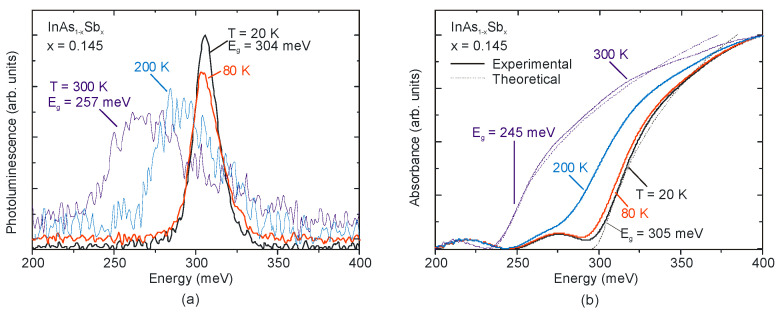
PL (**a**) and absorbance (**b**) spectra of 5-µm thick InAs_0.855_Sb_0.145_ MBE epilayer deposited on GaAs substrate with 3-µm thick InAs buffer layer. The residual background donor concentration is 3 × 10^16^ cm^−3^ (after [47]).

The CB density of states is low due to the very small effective mass of electrons and filling the available band states can be realized by doping shifting the absorption edge to shorter wavelengths. This phenomenon has been recognized as the Burstein-Moss (BM) effect, which is shown in Figure 12 for n-type InAs_0.855_Sb_0.145_ epilayer with residual background donor concentration of 3 × 10^16^ cm^−3^.

The theoretically predicted absorption was calculated using the Kane model, including the BM shift according to Anderson theory [73]. As is shown, the experimental spectrum is in proper agreement with the theoretical absorption simulation at 20 K and 300 K. The bandgap energy values measured with IR transmission spectroscopy agree with those obtained from PL peaks, particularly at low temperatures.

The first measurements of transport properties of n-type InAsSb alloys were performed on samples prepared in the late 1960s by various freezing and annealing techniques [74,75]. The properties of high-quality InAsSb epitaxial layers with *x* < 0.35 fabricated by LPE are comparable to those of pure InAs (when *n* = 2 × 10^16^ cm^−3^, typical mobilities are 30.000 cm^2^/Vs at 300 K and 50.000 cm^2^/Vs at 77 K). For InSb-rich alloys with *x* ≥ 0.90, typical mobilities are 60.000 cm^2^/Vs at 300 K. When As is added to InAs_1−x_Sb_x_ alloys, the residual background concentration has increased to a low 10^17^ cm^−3^; in turn the mobility first increased and then dropped by a factor of 1.5 to 2 as the temperature decreases from 300 to 77 K. At the present stage of MBE-growth development, the background electron concentration in InAs_1–*x*_Sb*_x_* with 40% Sb at 77 K is as low as 1.5 × 10^15^ cm^−3^ [39].

The carrier mobility in the III–V binary compounds is primarily limited by scattering due to optical phonons, and ionized impurities with a contribution from alloy disorder in the corresponding ternary alloy. The last kind of scattering mechanism is so-called alloy scattering. Their relative contributions depend on temperature, carrier concentration, compensation, and alloy composition. Acoustic phonon and deformation potential, optical deformation potential, and neutral impurity scattering are minor effects under most conditions. The total carrier mobility *μ_tot_* in alloy InAs_1–*x*_Sb*_x_* can be simply expressed as [27]:

(8) $$\frac{1}{\mu_{tot}\left(x\right)}=\frac{1-x}{\mu_{totInSb}}+\frac{x}{\mu_{totInAs}}+\frac{x\left(1-x\right)}{C_{\mu }},$$

The first two terms of Equation (8) result from the linear interpolation scheme and the third term reflects the effects of alloying.

**Figure 13:**
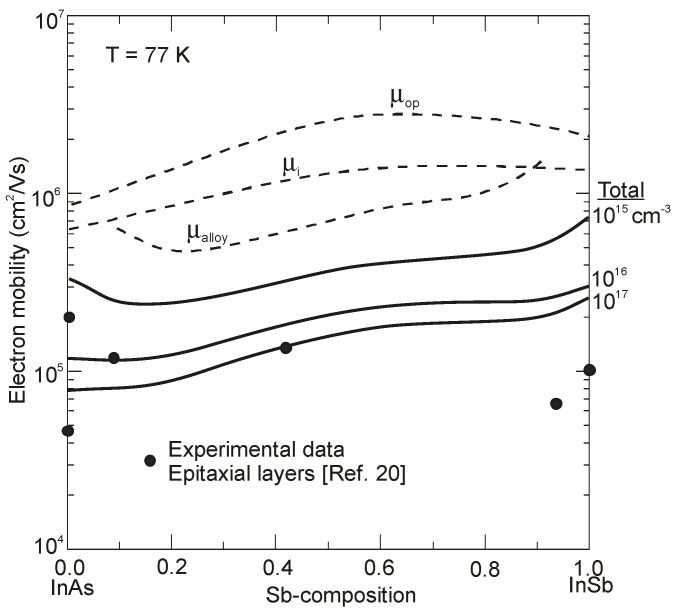
InAs_1−x_Sb_x_ mobility composition dependence at 77 K. The solid lines are the calculated mobilities for selected carrier concentrations, and the dashed lines are the component mobilities determined for a carrier concentration of 10^15^ cm^−3^ [77]. Experimental data is marked for epitaxial layers with carrier concentrations between 10^15^ to 10^16^ cm^−3^. Scattering legend: µ_op_-optical phonons, µ_i_-ionized impurity, µ_alloy_-alloy disorder.

The InAs_1−x_Sb_x_ mobility composition dependence is illustrated in Figure 13 at 77 and 300 K for carrier concentrations of experimental significance [76,77]. The component mobilities are shown for carrier concentrations of *n* = 10^15^, 10^16^ and 10^17^ cm^−3^ at 77 K, and 10^16^, 10^17^ cm^−3^ at 300 K. A decrease in impurity concentration causes continuous increase of carrier mobility throughout the temperature range. The significance of alloy scattering is visible when temperature and carrier concentration decrease as the respective contributions from polar optical phonons and ionized impurities are reduced, until it remains as the dominant scatterer across most of the compositional range for low carrier concentrations (below 10^15^ cm^−3^) and low temperatures.

**Figure 14:**
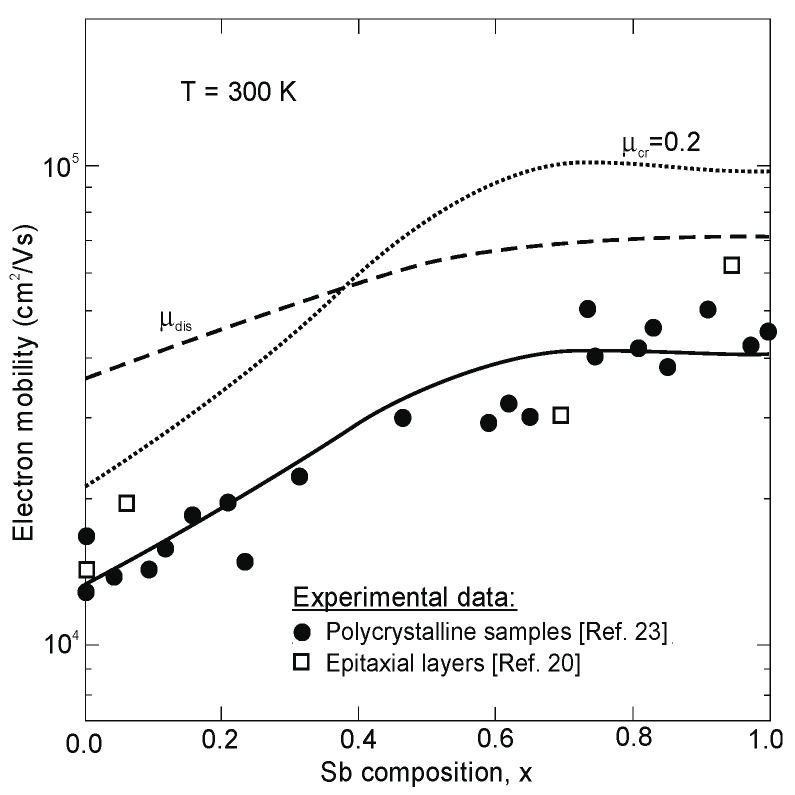
InAs_1−x_Sb_x_ electron mobility composition dependence for room-temperature (solid lines [77]) and carrier concentration of 10^17^ cm^−3^ with a compensation ratio of 0.2 and the effects of a dislocation density of 3.8 × 10^8^ cm^−3^. The experimental data is taken after [20,23] for samples with electron concentrations above 10^16^ cm^−3^.

Egan et al. have calculated the electron mobility of InAsSb by considering all the possible scattering mechanisms: impurities, acoustic phonons, optical phonons, alloy scattering, and dislocations [77]. Comparison with experiment confirms that dislocation scattering has a strong effect on transport, while alloy scattering limits mobility in ternary samples grown with a minimum of defects (see Figure 14).

**Figure 15:**
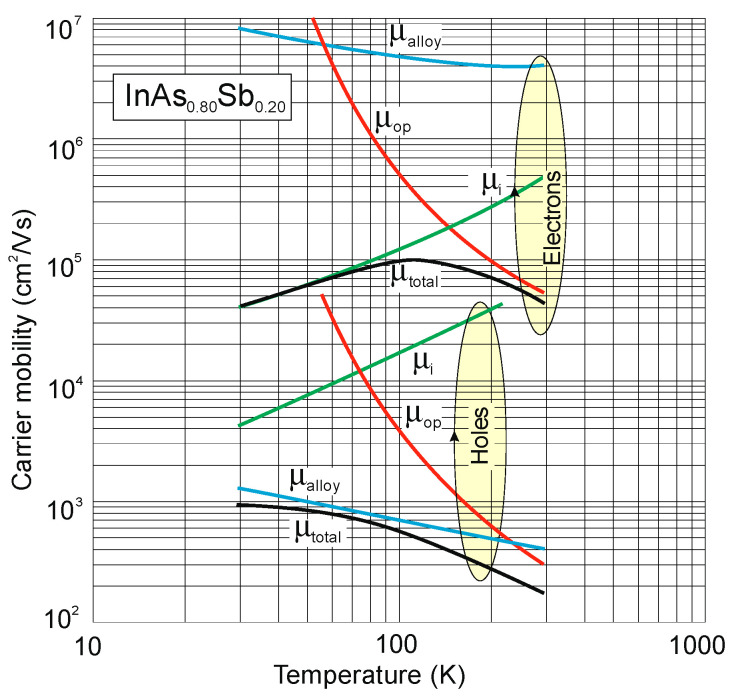
Modeled mobility versus temperature for electrons and holes in undoped InAs_0.80_Sb_0.20_.

The next figure (Figure 15) presents the modeled InAs_0.80_Sb_0.20_ electron and hole mobilities temperature dependence with low residual background doping [78]. As is shown, the mobility ratio *b* = *μ_e_*/*μ_h_* assumes ~10^2^, similarly to InSb.

### 3.4. Thermal Generation-Recombination Processes

The photodetector’s performance depends directly on the generation-recombination (G-R) processes, determining a steady-state concentration of carriers in semiconductors based on the optical (kinetics of photogenerated carriers) and thermal excitation. A wide study of G-R processes in semiconductors is reported in literature (see for example [79,80,81]). We describe here only selected carrier lifetime results directly related to photodetector performance. Assuming bulk processes only, there are three main thermal G-R processes to be considered in the narrow bandgap semiconductors: SRH, radiative, and Auger.

The statistical theory for the G-R processes via intermediate centers was developed first by Shockley and Read [82], and Hall [83]. It may be reduced by decreasing foreign impurities and native defects concentrations, which can be achieved by low-temperature growth and by progress in the material’s development. Since the SRH does not represent a fundamental limit to photodetector performance (it can be reduced with progress toward purer and higher-quality material), a significant research effort is still needed. 

Absorption of internally generated photons causes the radiative generation of charge carriers. The annihilation of electron-hole pairs with emission of photons makes an inversed process. Internal radiative processes are the fundamental factor limiting the detector performance. However, critical re-examination of the radiative mechanism contribution in the IR detection has been presented in the literature [84,85]. Humpreys [84] indicated that due to photon reabsorption (PR), the radiative lifetime is highly extended and depends on the semiconductor geometry. Under reverse bias operation where the electron density in the active layer is reduced below its equilibrium level, the internal radiative generation could be suppressed [86]. In spite of the fundamental nature, the radiative processes do not limit significantly the ultimate performance of IR detectors, especially in the LWIR range [87].

**Figure 16:**
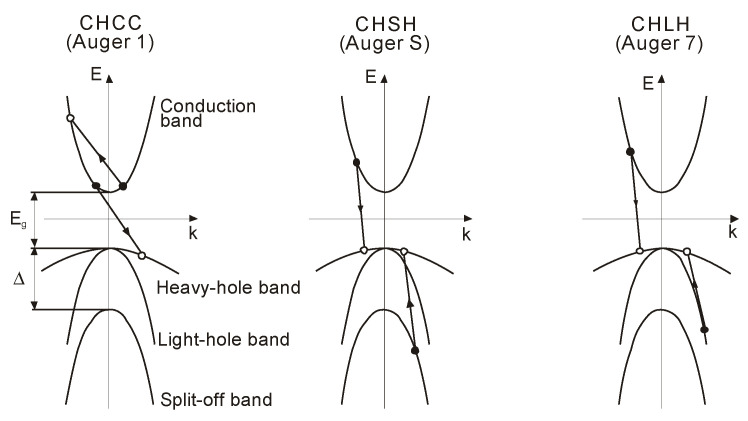
The three BtB Auger recombination processes. Arrows indicate electron transitions; •, occupied state; ○, unoccupied state.

In high-quality narrow gap semiconductors such as InSb and Hg_1−x_Cd_x_Te, Auger mechanisms determine G-R processes at near room temperatures. The Auger mechanisms in InSb-like band structure materials are classified into 10 photonless mechanisms according to the related bands. Figure 16 presents the three predominant mechanisms in the case of this type of band structure. They are characterized by the largest combined density of states and smallest threshold (*E_T_* ≈ *E_g_*).

The conduction band/heavy-hole band/conduction band (CHCC) recombination mechanism (also labeled Auger 1) prevails in n-type material and involves two electrons and a heavy hole. For p-type material the conduction band/heavy-hole band/light-hole band (CHLH) process (marked as Auger 7) is characteristic under condition that the spin split-off band can be neglected. Auger transition probability through the conduction band/heavy-hole band/spin split-off band mechanism (labeled CHSH or Auger S hereafter) can be ignored for semiconductors such as InSb and HgCdTe where the spin split-off energy (∆) is much larger than the bandgap energy *E_g_* and the Auger 7 transition is typical.

The spin split-off band is even more essential than the light-hole band for the direct-bandgap materials, particularly when the bandgap energy *E_g_* approaches the spin-orbit splitting ∆ (as in the case of InAs and InAsSb). The carrier lifetimes in binary compounds (InSb and InAs) are discussed in detail in chapter 2 monograph *Antimonide-Based Infrared Detectors. A New Perspective* [88].

**Figure 17:**
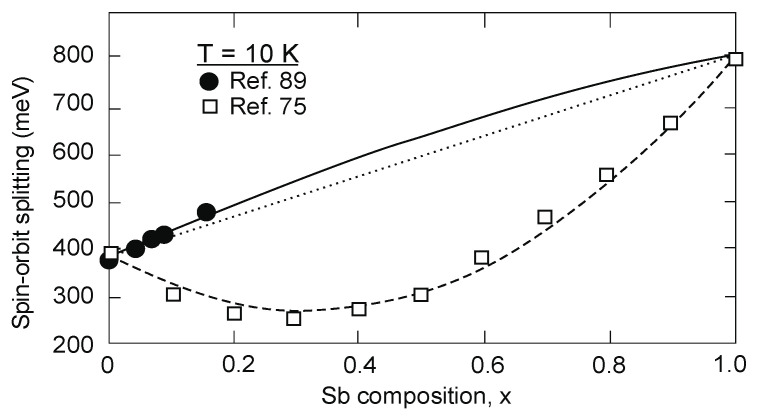
InAs_1−x_Sb_x_ spin-orbit-splitting energy versus composition at *T* = 10 K. The dotted straight line represents the zero bowing behavior consistent with the VCA. The single points represent experimental data [75,89].

Figure 17 summarizes the InAs_1−x_Sb_x_ spin-orbit-splitting energy, ∆, composition dependence at 10 K. Experimental results of ∆(*x*) dependence published in 1972 by Van Vechten et al. [75] differ more strongly than that presented recently in the paper by Cripps et al. [89]. The ∆ parameter versus *x* (Sb fraction) characteristic does not show bowing clearly. A good approximation can be calculated with

(9) Δ(x)=0.81x+0.371(1−x)+0.165x(1−x)

The above formula allows for the calculation of the spin-orbit-splitting bandgap energy which stays in a good agreement with experimental results and does not depend on temperature.

For slight deviation from equilibrium and for nondegenerate materials with equilibrium carrier concentration *n_o_* (or *p_o_*), the carrier lifetimes can be calculated using the intrinsic carrier lifetime and *n_o_*/*n_i_* (or *p_o_*/*n_i_*) ratio [65,79]:

for radiative recombination:

(10) $$\tau_{R}^{h}=\frac{2\tau_{R}^{i}}{\left(n_{o}/n_{i}\right)+\left(n_{i}/n_{o}\right)}\tau_{R}^{e}=\frac{2\tau_{R}^{i}}{\left(p_{o}/n_{i}\right)+\left(n_{i}/p_{o}\right)},$$

for Auger recombination:

(11) $$\tau_{A1}=\frac{2\tau_{A1}^{i}}{1+{\left(n_{o}/n_{i}\right)}^{2}}\;\;\;\;\;\;\tau_{A7}=\frac{2\tau_{A7}^{i}}{1+{\left(p_{o}/n_{i}\right)}^{2}}\;\;\;\;\;\;\tau_{AS}=\frac{2\tau_{AS}^{i}}{1+{\left(p_{o}/n_{i}\right)}^{2}}.$$

The InAs_1−x_Sb_x_ Auger and radiative recombination carrier lifetime for the composition range 0 ≤ *x* ≤ 1 and the temperature range 77–300 K was estimated by Rogalski and Orman [90]. Radiative recombination determines the carrier lifetime in the low temperature range. At higher temperature, the Auger 1 process is dominant in n-type InAs_1–*x*_Sb*_x_* but in p-type material, for the composition range 0 ≤ *x* ≤ 0.3, Auger 7 and Auger S processes compete while for *x* > 0.3, the Auger 7 process dominates. Different recombination mechanisms in InAs_0.35_Sb_0.65_, operating at the longest *λ_cut-off_*, (with *E_g_* close to 0.1 eV at room temperature) are also discussed in [90].

**Figure 18:**
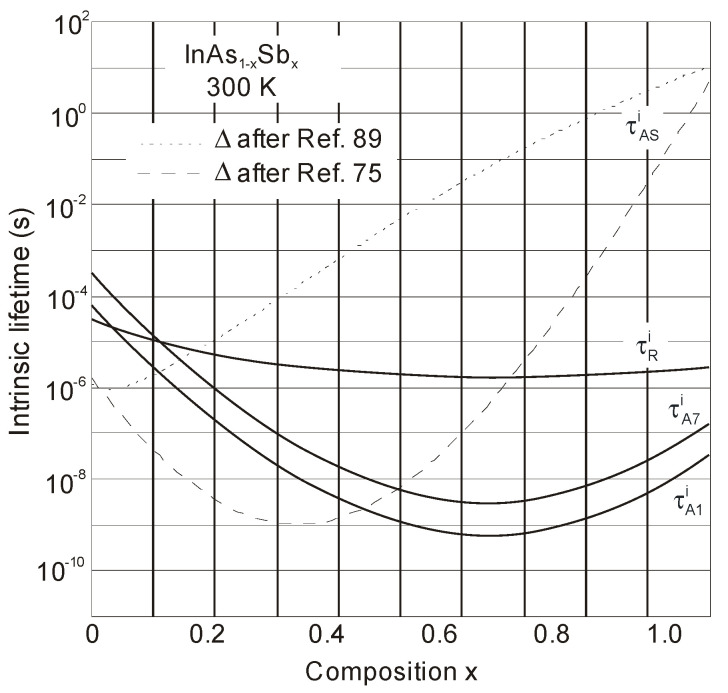
Intrinsic InAs_1−x_Sb_x_ Auger and radiative recombination carrier lifetimes at room temperature. $\tau_{S1}^{i}$ is calculated assuming spin-orbit-splitting bandgap energies after [75,89] (after [91]).

The dependence of $\tau_{R}^{i}$, $\tau_{A1}^{i},\tau_{A7}^{i}$ and $\tau_{AS}^{i}$ on the composition *x* in InAs_1–*x*_Sb*_x_* at room temperature is presented in Figure 18. The effect of the Auger S process was considered as accurate for 0 ≤ *x* ≤ 0.3 according to the out-of-date carrier lifetime calculation [90]. The predicted contribution of Auger S process is dominant in InAs_1−x_Sb_x_ semiconductors close to InAs (for 0 ≤ *x* ≤ 0.15) taking more recently estimated dependence of ∆(*x*) [89]. It follows that InAsSb photodiodes have lower Auger S nonradiative losses than were reported previously [91]. This adjustment has substantial implications on the p-type InAsSb-based devices’ design.

Generally, in III–V semiconductors the carrier lifetime is not limited by the BtB recombination but by SRH mechanism. Two regions are of interest:

- the neutral or diffusion region of the detector, and
- the depletion region of the detector.

As discussed in many papers, there are two types of possible SRH defects-neutral centers at the intrinsic level with carrier lifetimes *τ_po_* ~ *τ_no_* and charged centers at some specific energy level in which there is a large asymmetry in the lifetimes associated with electrons and holes. 

**Figure 19:**
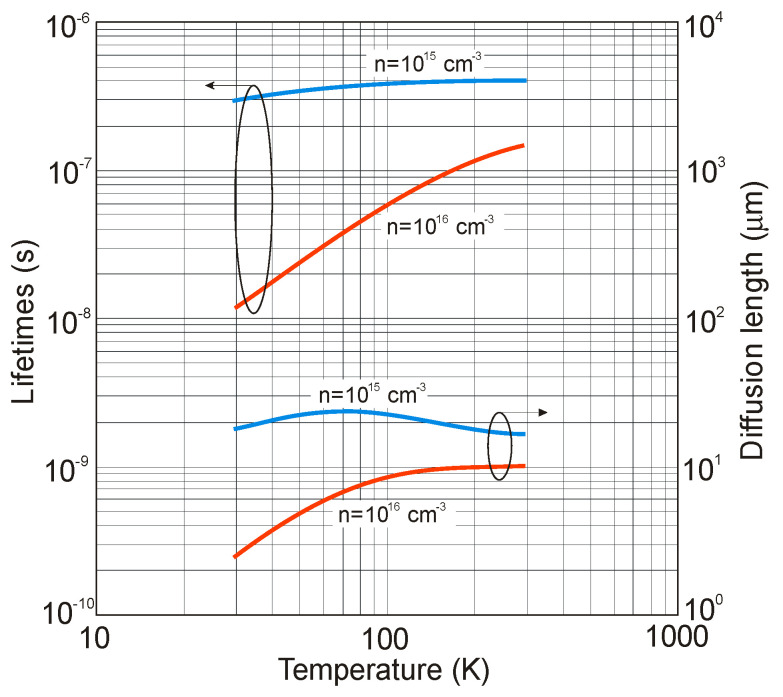
Simulated minority carrier lifetime and diffusion length versus temperature for holes in n-type InAs_0.81_Sb_0.19_ with doping levels 10^15^ and 10^16^ cm^−3^ and *τ_SRH_* = 400 ns.

In comparison with II–VI HgCdTe, the reported SRH lifetimes in the majority of III–V binary alloys are not impressive. For example, the best values for InSb indicate that *τ_SRH_* is about 400 ns for LPE-grown material [92]. It is interesting to stress that this value has not been improved over the last 60 years, and no response defect has been identified. Similar lifetime was reported for MBE grown InAs/InAs_0.72_Sb_0.28_ T2SLs. Assuming this carrier lifetime value, Kinch modeled the minority carrier lifetime and diffusion length versus temperature for holes in n-type InAs_0.81_Sb_0.19_ with doping levels 10^15^ and 10^16^ cm^−3^-see Figure 19 [78]. These values are approximately three orders of magnitude smaller than those in HgCdTe with similar bandgaps.

### 3.5. Other Properties

**Figure 20:**
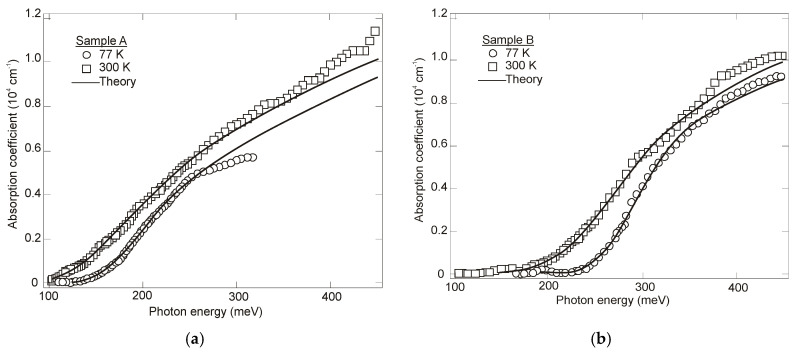
Absorption spectra for InAs_0.30_Sb_0.70_ grown on GaAs substrates at 300 and 77 K with electron concentrations (**a**) 10^17^ cm^−3^ and (**b**) 2 × 10^18^ cm^−3^. Solid lines present theoretical fitting (after [93]).

The optical properties of InAsSb are determined by a nonparabolic three-band model. Dobbelaere et al. [93] have studied IR absorption spectra of Si-doped MBE epitaxial layers grown on Si and GaAs substrates. The absorption coefficient was measured in the wavelength range 3–12 μm and the experimental data were fit by an analytical formula that was derived from the Kane band model. Excellent agreement between the calculations and the measurements was observed (Figure 20). Due to the fact that the measurements were carried out for InAs_1−x_Sb_x_ with *x* ≈ 0.70, a large shift of the absorption edge with Si doping was observed due to low density of states in the CB. This has been referred to the BM effect.

**Figure 21:**
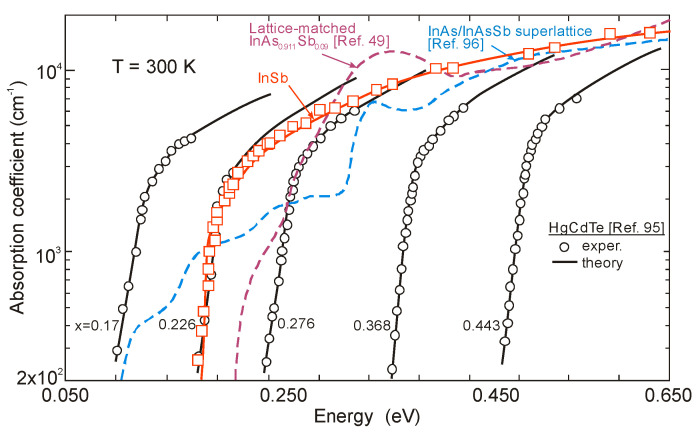
Intrinsic absorption coefficient for Hg_1−x_Cd_x_Te, InSb, and lattice-matched InAs_0.911_Sb_0.09_ and InAs/InAsSb SLs at room temperature.

Comparable values of absorption coefficients for bulk HgCdTe [94,95] and InAsSb [49,96] are reported (see Figure 21), proving relatively minor differences between densities of states in bulk semiconductors with the same energy gap and the optical matrix elements.

**Figure 22:**
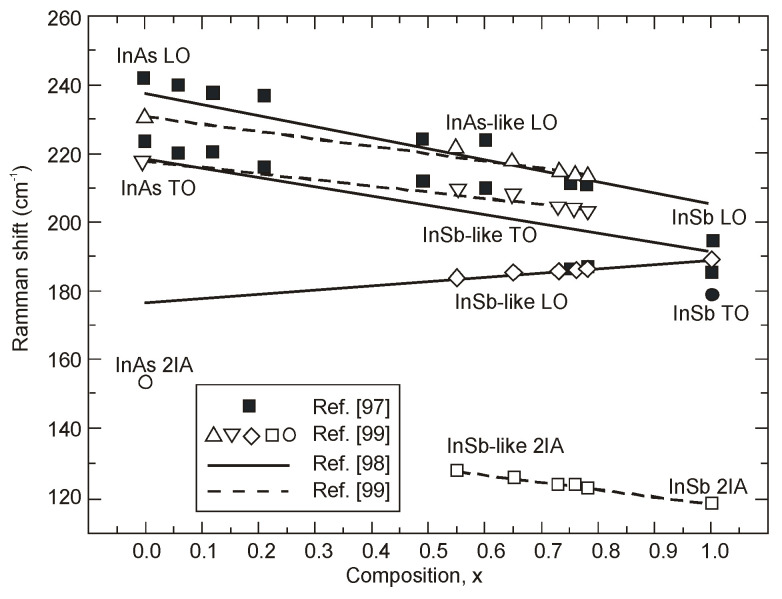
InAs_1−x_Sb_x_ Raman frequency dependence versus composition (after [99]).

The Raman spectroscopy is a well-known technique to understand the lattice vibrations of various material systems. The first Raman spectroscopy study of InAs_1−x_Sb_x_ on InAs and InSb substrates was performed by Cherng et al. [97]. They reported “one-mode” phonon behavior for *x* ≤ 0.6 and “two-mode” phonon behavior for larger values. Then, a similar study for InAsSb grown with different composition on GaAs substrate by MBE was carried out in [98,99], and “two-mode” phonon behavior was observed. Along with the Sb composition increasing in InAs-like longitudinal-optical (LO), transverse-optical (TO) phonon peaks reveal a blue and red-shift occuring for InSb-like LO phonon peak-see Figure 22. Recently, Grodecki et al. [100] suggested that spectra in the region 20–160 cm^−^^1^ are caused by zone folding in phonon dispersion curves as a result of CuPt unit cell of InAsSb being twice as large as typical ZB unit cell.

## 4. Properties of InAs/InAsSb Superlattices

Ting et al. published simple theoretical considerations on the fundamental T2SLs parameters [101]. Their properties differ strongly from those of constituent layers.

InAs/InAsSb SLs have been less researched than InAs/GaSb SL and they are in the early stage of development. Because of only two common elements (In and As) in SL layers and rather uncomplicated interface structure with Sb-shifting elements, the InAs/InAsSb SLs growth follows with a better controllability and easier manufacturability. It appears that InAs/InAsSb SLs are more flexible in the optimization of device performance. In order to reduce the tension between the SL layers and the substrate on which the SL is deposited, a suitable selection of the buffer layer is required in which the tension between the SL and the buffer layer is averaged. Usually, in order to fabricate the defect-free buffer layer, the interfacial misfit array (IMF) technique is applied [102]. By producing the strain-balanced SL to the buffer layer, the influence of the strains and dislocations on the device performance is eliminated.

The carrier localization effect is noticed in T2SLs at low temperature because of local impurities and spatial variations in the CB and VB potentials, layer thickness variation, or composition variation. These local potentials result in low-energy tail states (lower than the bandgap), which can trap carriers. The presence of carrier localization is supported by observation of extremely long carrier lifetimes and PL peak blue shifts at low temperatures [20,103]. The InAs/InAsSb interface disorder was found to be the reason for the carrier localization [104]. Therefore, the greater effect from interface disorder and deeper localization potentials are observed in smaller SL periods. The carrier localization effects are not observed at higher temperatures (>100 K).

### 4.1. Bandgap Energy and Effective Masses

**Figure 23:**
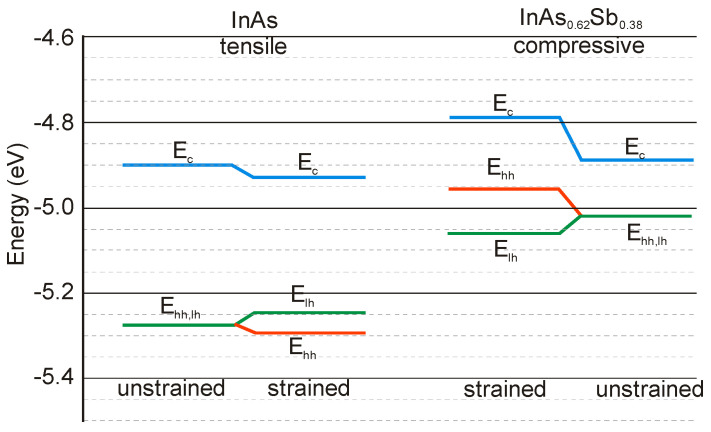
The energy band profiles of unstrained and strained InAs/InAs_0.62_Sb_0.38_ SLs (after [105]).

Since InAs is not lattice-matched to InAsSb, the changing of the layer thickness requires a good understanding of the strain contribution on the material quality. Figure 23 shows the SLs InAs/InAs_0.62_Sb_0.38_ band structure for unstrained and strained conditions. Under the influence of strain, an electron affinity changes and the VB splits on the heavy and light holes’ sub-bands.

**Figure 24:**
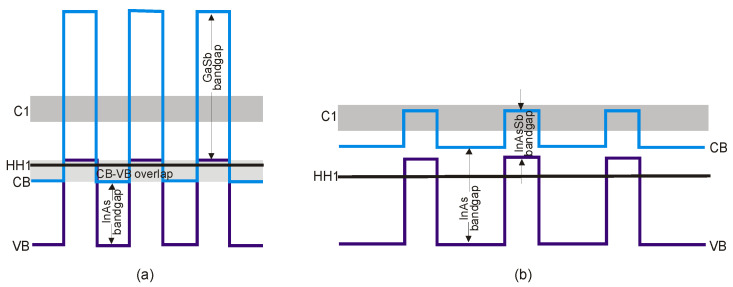
Bandgap diagram for (**a**) InAs/GaSb and (**b**) InAs/InAsSb T2SLs (after [106]).

The basic difference in profiles of the CB and VB in the InAs/GaSb and InAs/InAsSb T2SLs is presented in Figure 24. The band alignment of T2SL is causing a state in which the energy bandgap of the SLs can be adjusted to the configuration, either a semimetal or a narrow bandgap semiconductor material. 

Transitions between electron and hole bands are spatially indirect. The first electron miniband (C_1_) is more sensitive to layer thickness than first heavy hole state (HH_1_) because of the large value of the heavy-hole mass.

Below are emphasized distinctions in the fundamental properties of InAs/GaSb and InAs/InAsSb SLs:

- both type of T2SLs are based on nearly-lattice-matched III-V semiconductors and provide a large range of tunability in *λ_cut-off_*,
- in T2SLs the electron and hole wave functions are located in separate layers,
- the resulting energy gap is determined by the transition energy between the HH_1_ and the C_1_ and depends upon the layer thicknesses and interface compositions,
- the band offsets in conduction (∆*E_c_*) and valence (∆*E_v_*) bands in the InAs/InAsSb SL (∆*E_c_* ~142 meV, ∆*E_v_* ~226 meV) are much smaller as compared to InAs/GaSb SL (∆*E_c_* ~930 meV, ∆*E_v_* ~510 meV),
- a much larger broken gap of the InAs/GaSb SLs makes it easier to reach small SL bandgaps,
- as the period increases, the *λ_cut-off_* of the InAs/GaSb SL increases much faster than in the case of the InAs/InAsSb SL. Shorter period of InAs/GaSb SL gives the same *λ_cut-off_* as for InAs/InAsSb SL.

**Figure 25:**
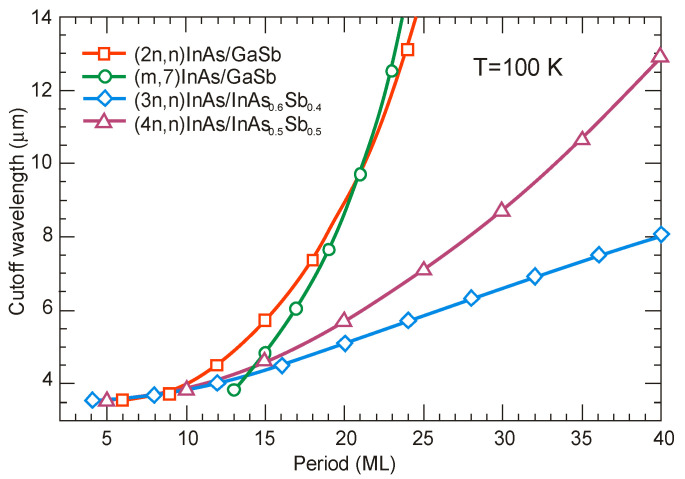
Theoretically estimated *λ_cut-off_* for (2n,n)InAs/GaSb, (m,7)InAs/GaSb, (3n,n)InAs/InAs_0.6_Sb_0.4_, and (4n,n)InAs/InAs_0.5_Sb_0.5_ SLs at 100 K (all on GaSb substrate) versus SLs period in MLs (after [107]).

Figure 25 shows the theoretically estimated the *λ_cut-off_* versus SL period for strain balanced (with respect to the GaSb substrate) InAs/GaSb and InAs/InAsSb T2SLs. In order to achieve strain balance between InAs/GaSb SLs layers, InAsSb interfacial MLs are inserted between the InAs and GaSb layers. As is shown in Figure 25, the (m,n)A/B denotation means SLs with MLs of material “A” and MLs of material “B” in each period of (m + n) MLs.

**Figure 26:**
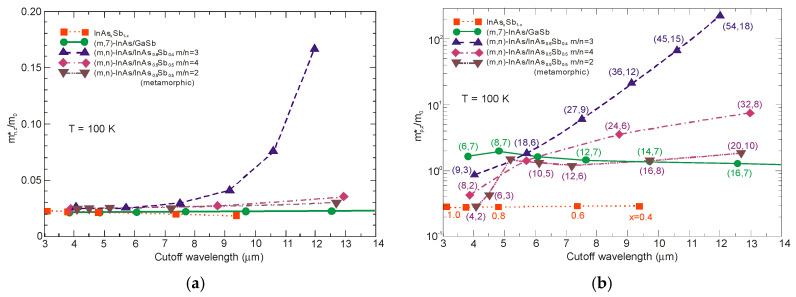
Electron (**a**) and hole (**b**) effective masses along the growth direction versus *λ_cut-off_* for different material systems: free-standing bulk InAsSb with varying Sb composition *x*, (m,7)-InAs/GaSb T2SL, pseudomorphic (m,n)-InAs/InAs_0.6_Sb_0.4_ T2SL on GaSb substrate, pseudomorphic (m,n)-InAs/InAs_0.5_Sb_0.5_ T2SL on GaSb substrate and (m,n)-InAs/InAs_0.5_Sb_0.5_ T2SL on a metamorphic substrate with lattice constant 0.4% larger than that for GaSb (after [107]).

Due to the considerably larger effective mass along the growth direction than those in bulk semiconductors, particularly in LWIR range, T2SLs are characterized by a low mobility, a short diffusion length, and low collection efficiency in n-type devices. Figure 26 shows a comparison of the calculated electron and hole effective masses for bulk InAsSb and both InAs/GaSb and InAs/InAsSb T2SLs versus *λ_cut-off_* [107,108]. Generally, the rate of increase for both $m_{e,z}^{*}$ and $m_{h,z}^{*}$ versus *λ_cut-off_* is faster in SL requiring longer periods to reach the same *λ_cut-off_*. An increase of the SL period to reach longer *λ_cut-off_*, leads to the weaker coupling between wave functions and in consequence results in larger effective masses. The relative insensitivity of the InAs/GaSb T2SL effective mass along the growth direction, $m_{e,z}^{*}$, versus *λ_cut-off_* results from the fact that the HH_1_ state is located within the broken gap energy range. We can notice that in properly designed T2SLs, the electron effective masses are comparable to those in bulk material. However, in the LWIR region the hole effective masses along the growth direction of T2SLs are noticeably larger than those in bulk. In comparison with HgCdTe with the bandgap energy *E_g_* ≈ 0.1 eV and $m_{e}^{*}=0.009m_{0}$, the electron effective mass of T2SLs is larger. Comparison of both types of the SLs in MWIR and LWIR ranges indicates that the InAs/GaSb T2SL would have smaller $m_{h,z}^{*}$ in the very-long wavelength (VLWIR). 

### 4.2. Absorption Coefficient

**Figure 27:**
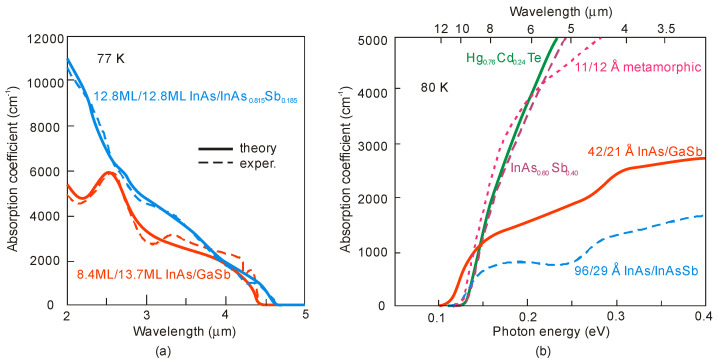
Experimental and theoretical absorption spectra for MWIR InAs/GaSb and InAs/InAsSb T2SLs at 77 K (**a**) (adapted after [110]) and LWIR HgCdTe and different III-V material systems at 80 K (**b**). (**b**) presents only calculated interband absorption coefficients for bulk InAs_0.60_Sb_0.4_, Hg_0.76_Cd_0.24_Te, and T2SLs: 42Å InAs/21Å GaSb, 96Å InAs/29Å InAs_0.61_Sb_0.39_ and 11Å InAs_0.66_Sb_0.34_/12Å InAs_0.36_Sb_0.64_ metamorphic.

The InAs/InAsSb T2SL with an average lattice constant matched to GaSb have significantly lower absorption than InAs/GaSb one (see Figure 21 and Figure 27). It is related to the overlapping of the electron-hole wave functions, which in the case of the InAs/InAsSb T2SL occurs primarily in the well for holes having a relatively small thickness throughout the SL period. The InAs/GaSb SLs have the more favorable oscillator strengths and absorption coefficients than InAs/InAsSb, especially in VLWIR spectral range. However, due to weaker confinement of the CB, the more delocalized electron wave functions help somewhat in enhancement of the absorption properties of the InAs/InAsSb T2SLs. Generally, a shorter SL period provides a larger absorption coefficient due to a better wave function overlap and greater oscillator strength.

In general, the absorption coefficient near *λ_cut-off_* is weaker for the InAs/InAsSb SL than for the InAs/GaSb SL. From the other side (see Figure 21), the absorption coefficient of HgCdTe is stronger than those for both T2SLs. In addition, theoretical estimates of the absorption spectra presented by Vurgaftman et al. [109] for LWIR T2SLs and bulk materials (HgCdTe and InAsSb) support above observations-see Figure 27b. In addition, these estimates show that only for the small-period metamorphic InAs_1−x_Sb_x_/InAs_1-y_Sb_y_ SLs, its value of absorption coefficient is comparable with bulk materials.

Figure 27a demonstrates experimental and theoretical absorption coefficient for InAs/GaSb and InAs/InAsSb T2SLs [110,111]. Theoretical calculations in Figure 27a reproduce well the experimental absorption coefficient for 12.8ML/12.8ML InAs/InAsSb SL and for the 8.4ML/13.7ML InAs/GaSb SL. Visible for the 8.4ML/13.7ML InAs/GaSb SL strong peak at a wavelength of about 2.6 μm is the result of the zone boundary HH_2_ → C_1_ transitions. Another slightly weaker peak at wavelength of about 3.4 μm is due to the zone centre LH_1_ → C_1_ transition. 

### 4.3. Carrier Lifetimes

Apart from simpler manufacturability of InAs/InAsSb T2SLs in comparison with InAs/GaSb system, the interest in InAs/InAsSb SLs stemmed mainly due to the lack of restriction on the carrier lifetime resulting from the Ga presence in the InAs/GaSb SL.

In general, the carrier lifetime is enhanced in T2SLs due to suppression of Auger mechanisms resulting from the separation of electrons and holes. Since the optical transitions occur spatially, the optical matrix element for BtB transitions is relatively small. Theoretical calculations show that Auger recombination rates in T2SLs are suppressed by several orders in comparison to the bulk HgCdTe optimized at the same *λ_cut-off_*. Nevertheless, these theoretical considerations have not been experimentally confirmed yet. In practical devices based on III-V material systems, more active SRH centers are observed, compared to those of HgCdTe ternary alloys, resulting in lower carrier lifetime. What is worth noting is that the SRH carrier lifetime in InSb has been on the same level since the beginning of research in the 1950s. The situation is more complex in the case of T2SLs.

In accordance with the statistical theory of the SRH mechanism, the shortest SRH lifetime occurs through the trap centers located approximately in the intrinsic energy level in the bandgap. The location of the Fermi level has an impact on the location of the energy level associated with native defects. For example, the Fermi level in bulk GaSb is located either in the mid-gap or near the VB edge, while in bulk InAs is located above the CB edge. Thus, the trap level located in the mid-gap of GaSb promotes the SRH recombination, whereas in InAs trap centers are inactive for the SRH process. For this reason, the carrier’s lifetime in bulk InAs is longer than in bulk GaSb. It can be assumed that the native defects associated with the GaSb layer (SRH centers has been attributed to the presence of gallium) in InAs/GaSb T2SL are responsible for the SRH-limited minority carrier lifetime. The gallium-free (Ga-free) SLs show much longer carrier lifetimes, up to 10 μs for undoped material in MWIR region [112,113]. The minority carrier lifetime in InAs/InAsSb SLs increases versus Sb composition and lower thickness of InAsSb layer.

**Figure 28:**
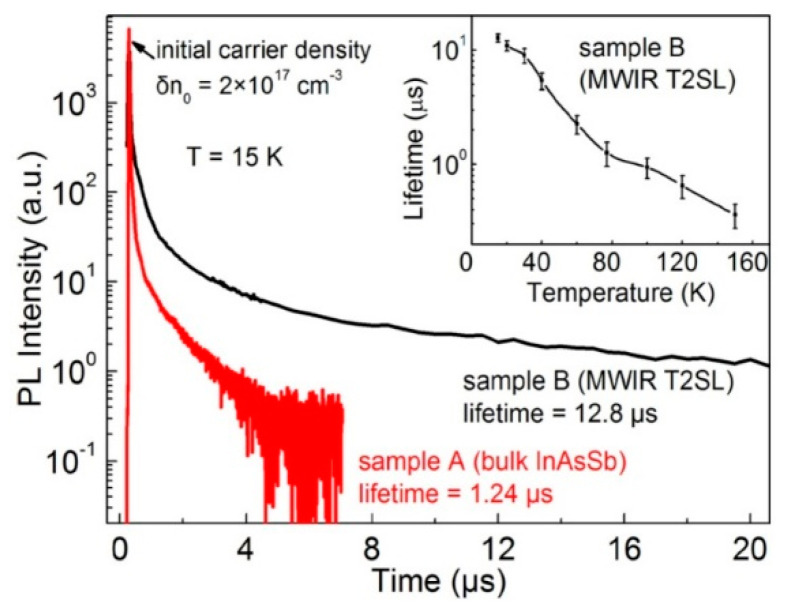
Time-resolved PL decays of samples A (bulk InAsSb) and B (MWIR InAs/InAsSb T2SL) at 15 K. The minority carrier lifetimes of 1.24 µs and 12.8 µs are extracted from the single exponential decays, respectively. The inset shows the carrier lifetime of sample B versus temperature (after [104]).

At low temperature, the carrier lifetime in the InAs/InAsSb SLs is enhanced by the carrier localization effect. The localized trap states tend to be in resonance with the CB rather than in the bandgap. As shown in the inset of Figure 28, the minority carrier lifetime for MWIR T2SLs decreases to 1.26 µs versus temperature and for *T* = 77 K is an order of magnitude shorter than that at 15 K. It is suggested that at 15 K the photo-generated electron-hole pairs recombine through spatially separated localized states. A long minority carrier lifetime of 12.8 µs is due to a small overlapping of the wave functions.

In devices operating at higher temperatures (typically above 80 K), no deleterious effects from carrier localization are observed. However, for detectors with low background doping, operating at low temperatures (typically below 80 K) and under low incident light intensities, carrier localization may be visible; e.g., reduced carrier collection efficiency resulting in lower *QE*.

**Figure 29:**
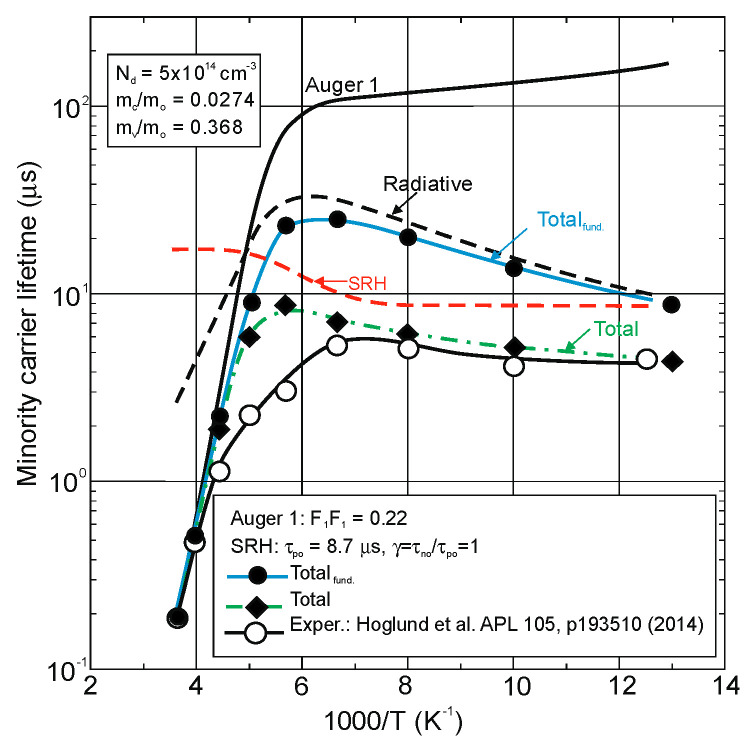
Hole minority carrier lifetime versus inverse temperature for a n-type MWIR InAs/InAs_0.54_Sb_0.46_ T2SL with doping concentration 5 × 10^14^ cm^−3^ (after [114]).

Figure 29 presents the measured and modeled hole minority carrier lifetime versus inverse temperature for n-type InAs/InAs_0.54_Sb_0.46_ T2SL with doping concentration *N_d_* = 5 × 10^14^ cm^−3^ [114]. In the extrinsic region, (*N_d_* > *n_i_*), the lifetime is dominated by the SRH lifetime, *τ_SRH_*. Taking the measured lifetime to be 4.5 μs and the radiative lifetime of 9.3 μs, the calculated SRH lifetime is 8.7 μs, if recombination centre is located at the intrinsic Fermi level. The Auger hole lifetime curve is presented for overlap integral |F_1_F_2_| = 0.22.

**Figure 30:**
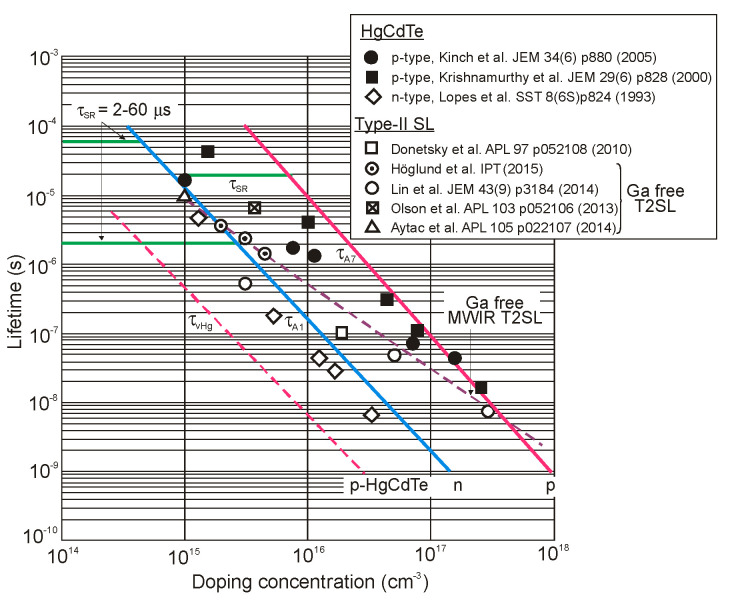
Minority carrier lifetimes versus doping concentration for MWIR HgCdTe and T2SLs at 77 K. Theoretical trend lines for n-type and p-type HgCdTe ternary alloys are taken from [115]. The dashed line for Ga-free T2SLs follows experimental data.

**Figure 31:**
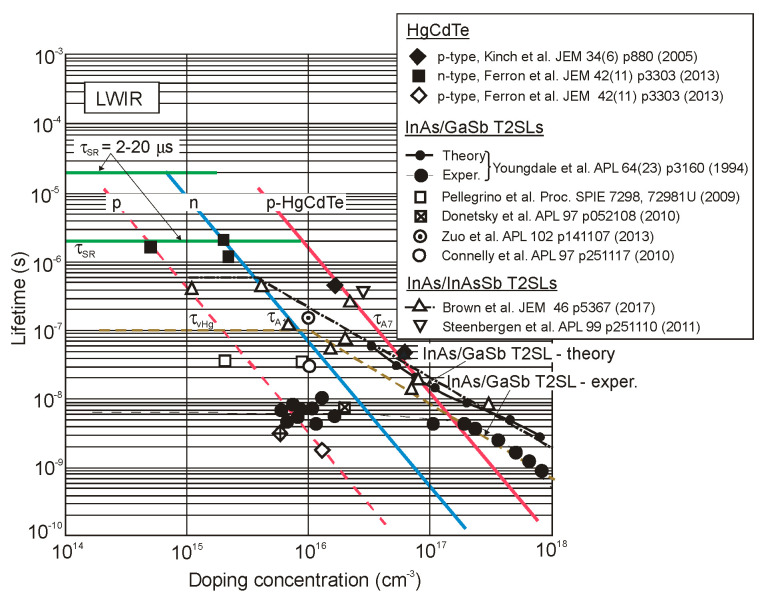
Minority carrier lifetimes versus doping concentration for LWIR HgCdTe and T2SLs at 77 K. Theoretical trend lines for n-type and p-type HgCdTe are taken from [115]. The dashed line for T2SLs follows experimental data.

The next two figures (Figure 30 and Figure 31) summarize carrier lifetimes data for bulk HgCdTe and both types of T2SLs (InAs/GaSb and InAs/InAsSb) operating in MWIR (*λ_cut-off_* ≈ 5 μm), and LWIR (*λ_cut-off_* ≈ 10 μm). The trend lines HgCdTe carrier lifetimes are given after Kinch et al. [115]. InAs/InAsSb SL system shows a significantly longer minority carrier lifetime (1 μs for MWIR material at 77 K) in comparison to the InAs/GaSb SL operating at the same wavelength range and temperature (~100 ns).

In lightly doped n- and p-type HgCdTe, the SRH mechanism determines the carrier lifetime. Generally, values of *τ_SRH_* are approximately more than two orders of magnitude larger for HgCdTe than those reported for the III-V semiconductors with similar bandgaps. At lower temperatures and doping level below 10^14^ cm^−3^, the HgCdTe P-i-N photodiodes become depletion limited due to SRH centers having lifetimes in the range to 10 ms [116,117]. In consequence, the potential properties of room-temperature HgCdTe photodiodes operating above 3 μm guarantee reaching more than order of magnitude higher detectivity (above 10^10^ Jones) in comparison with value predicted by Rule 07 [118], and this detectivity is limited by the background flux [119]. Up till now, long SRH lifetime of HgCdTe gives the potential to use this material system for background limited performance (BLIP) at room-temperature operation. 

## 5. InAsSb Bulk Photodiodes

**Figure 32:**
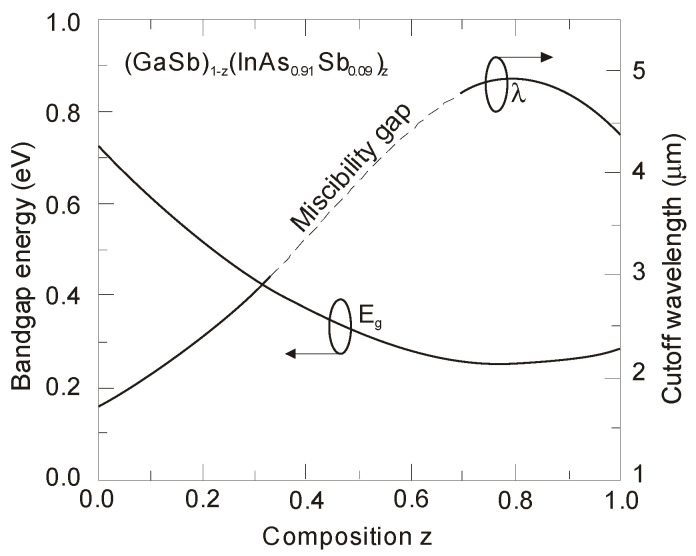
The energy bandgap of Ga_x_In_1−x_As_y_Sb_1–y_ with *x* and *y* compositions chosen in the ratio (GaSb)_1–z_(InAs_0.91_ Sb_0.09_)_z_ can be tuned continuously from ~475 to 730 meV while maintaining lattice-matching to a GaSb substrate.

All epitaxial techniques require large area, single crystal, and lattice-matched substrates. Ternary and quaternary layers lattice matched to InAs or GaSb substrates could be tailored to detect wavelengths in the range of 0.8 to 5 μm. As shown in Figure 32, the Ga_x_In_1–__x_As_y_Sb_1–__y_ energy bandgap can be continuously tuned from ~475 to 730 meV while maintaining lattice-matching to a GaSb substrate [50,120], which is not retained for materials in this range such as InGaAs on InP. Both ternary (GaInSb and InAsSb) and quaternary (GaInAsSb and AlGaAsSb) materials, still not available on the market, show good performance for wavelength range ≥2 μm at the research level. The emergence of new GaInSb virtual substrates has created a promising potential for development of high performance IR detectors. Several research groups have succeeded in growing GaInSb single crystals, where there is a need to underline the properties of the Ga_0.38_In_0.62_Sb lattice matched to InAs_0.35_Sb_0.65_, with Sb composition providing the lowest energy bandgap.

### 5.1. Technology and Properties

A variety of InAsSb photodiodes configurations have been proposed during the last forty years including n-p, n-p^+^, p^+^-n and P-i-N structures in both planar and mesa configurations. In addition, the techniques used to form p-n junctions were different, starting of Zn diffusion, through Be ion implantation in LPE technique, to more advanced epitaxial techniques such as MBE and MOCVD. The last two are predominant in fabrication of modern antimonide-based IR detectors, that essentially relies on n-type material with concentrations ~10^16^ cm^−3^. A summary of works for the fabrication of InAsSb photodiodes is given in recently published monograph [88].

**Figure 33:**
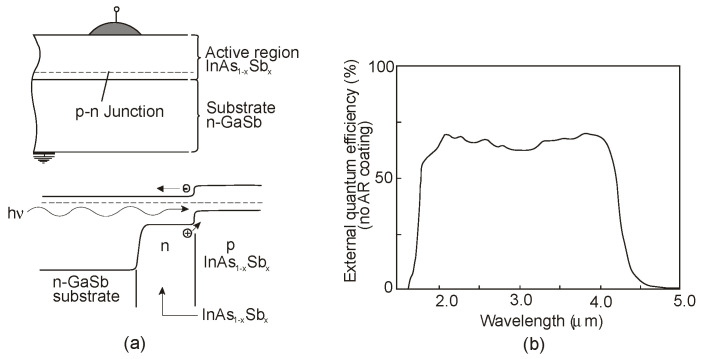
BSI InAs_0.86_Sb_0.14_/GaSb photodiode: (**a**) device structure and energy-band diagram of the structure, (**b**) spectral response at 77 K (after [9]).

Considerable step in development of InAsSb photodiodes was obtained in 1980 when nearly lattice-matched system of InAs_1−x_Sb_x_ (0.09 ≤ *x* ≤ 0.15) and GaSb were used [9]. Slight lattice mismatch up to 0.25% for the InAs_0.86_Sb_0.14_ epitaxial layer was accommodated in terms of low etch-pit density (≈10^4^ cm^−2^). Figure 33a shows a schematic structure of a back-side-illuminated (BSI) InAs_1−x_Sb_x_/GaSb photodiode. The wider-gap GaSb is a transmission window for photons reaching the InAs_1−x_Sb_x_ active layer and where they are absorbed. Part of the photons is also absorbed in GaSb substrate, which determines the cut-on wavelength (1.7 μm at 77 K), while the active region establishes *λ_cut-off_* = 4.3 μm (see Figure 33b). The InAs_1−x_Sb_x_ active layer was grown using LPE technique. Both undoped n-type layer and Zn doped p-type layer show the carrier concentrations of approximately 10^16^ cm^−3^. High quality of InAs_0.86_Sb_0.14_ photodiodes was demonstrated by a high *R_o_A* product in excess of 10^9^ Ωcm^2^ at 77 K.

**Figure 34:**
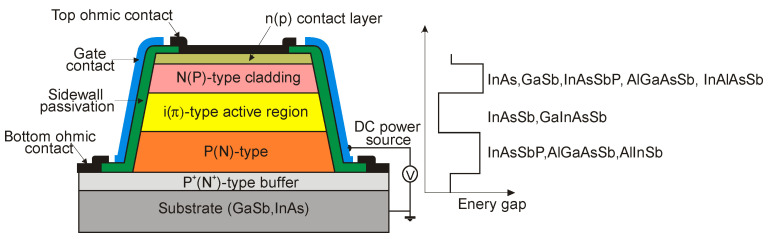
N-i-P double heterostructure photodiode schematic cross-section. Different combinations of III–V materials for active and cladding layers are also shown (after [91]).

P-i-N heterostructure device with an unintentionally doped InAsSb active layer (π or ν; doping level ~10^15^ cm^−3^) sandwiched between P and N wider-bandgap layers have been proposed by several groups to improve device performance at near-room temperature operation. Lower diffusion dark current, higher *R_o_A* product, and detectivity are related to the lack of injection of minority carriers from the wide-bandgap layers. Figure 34 shows schematic architecture of the N-i-P heterostructure and the different combinations of III-V materials for an active and contact layers. For InAsSb absorber, usually p-type GaSb and n-type InAs are used for the contact layers. Depending on the configuration of contact layers, both BSI and front-side illumination (FSI) can be used. However, for the BSI photodiode, the transparency of the substrates is also crucial. In spite of the relatively low absorption coefficients of commonly used substrates (GaSb, InAs), they are thinned to small thicknesses, even less than 10 μm. Sometimes, the substrates are completely etched off. What is more, many fabrication processes are not possible for InAs due to its fragility. The transparency of the substrates can be improved by using a heavy n-type doping and the use of the BM effect. A strong degeneracy of the electrons in the CB occurs already for a relatively low electron concentration (>10^17^ cm^−3^). For example, in a heavily doped n^+^-InAs (*N_d_* = 6 × 10^18^ cm^−3^), the BM shift makes the corresponding substrates transparent to the 3.3 μm [121]. 

**Figure 35:**
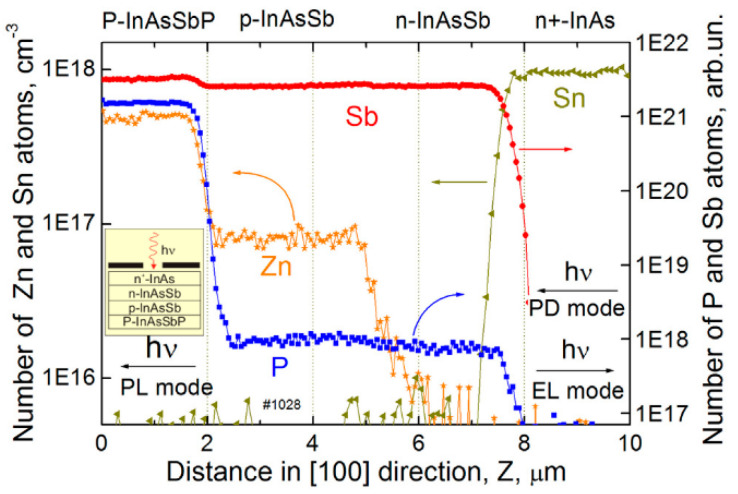
Distribution of P, Zn, Sn, and Sb atoms along the growth direction (SIMS data) of LPE-grown P-InAsSbP(Zn)/p-InAs_0.88_Sb_0.12_ (Zn)/n-InAs_0.88_Sb_0.12_/n^+^-InAs(Sn) DH photodiode. Arrows on the right and left denote photon flux with respect to heterostructure layers at the photoluminescence (PL), photodiode (PD), and electroluminescence (EL) modes (after [125]).

In addition, many of the investigations were made in the Ioffe Physical-Technical Institute, see [121,122,123,124], on different antimonide-based ternary and quaternary alloys as a materials for MWIR double heterostructure (DH) photodiodes for near-room temperature operation. These DH devices with about several-μm thick undoped n-InAs_1−x_Sb_x_ active layers and p-InAs_1−x−y_Sb_x_P_y_ 3-μm-thick Zn-doped cladding contact layers are LPE grown on n-type InAs(100) substrates (with *n* = 2 × 10^16^ cm^−^^3^ for undoped or *n*^+^ = 2 × 10^18^ cm^−^^3^ for the Sn-doped substrates). For example, Figure 35 presents the distribution of P, Zn, Sn, and Sb atoms along the growth direction of the BSI P-InAsSbP(Zn)/p-InAs_0.88_Sb_0.12_(Zn)/n-InAs_0.88_Sb_0.12_/n^+^-InAs(Sn) DH photodiode obtained by the secondary ion mass spectroscopy (SIMS). InAs_0.88_Sb_0.12_ absorber with smooth Zn distribution in the vicinity of the p-n junction is optimized for the MWIR range. 

**Figure 36:**
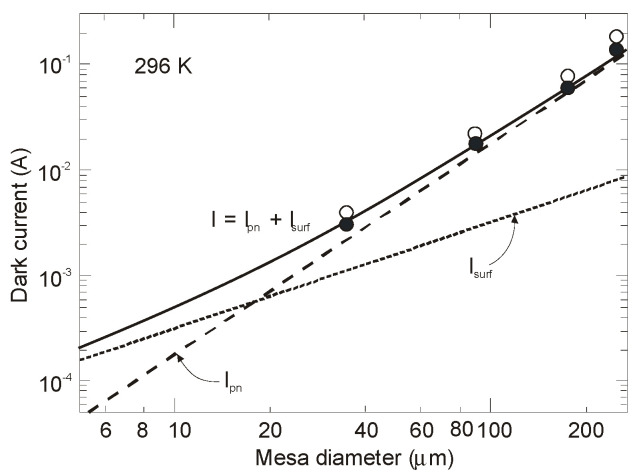
Total dark current at a reverse bias of 0.1 V (•) and 0.2 V (○) in InAsSbP/InAs_0.7_Sb_0.3_ DH photodiode at room temperature. The lines present the simulated bulk (*I_pn_*) and surface (*I_surf_*) leakage currents as well the sum of the above two currents at a reverse bias of 0.1 V (after [126]).

Figure 36 shows the detector dimensional dependence of dark currents for the InAsSbP/InAs_0.7_Sb_0.3_ DH photodiodes fabricated in Ioffe Institute with active 3-μm thick n-type InAsSb region. High circular mesas were defined by a standard optical photolithography and a wet chemical etching process. Next, circular Au- or Ag-based reflective anode and cathode contacts were formed by sputtering and thermal evaporation in vacuum. Finally, flip-chip bonding/packaging procedure has been implemented. Some chips were equipped with aplanatic hyperhemispherical Si immersion lenses (*Ø* = 3.5 mm) with antireflection coating. After [126], InAsSbP/InAs_0.7_Sb_0.3_ DH photodiodes demonstrate diffusion-limited current at near room temperature and tunnel current at 212–250 K with predominantly series resistance in the temperature range of 270–300 K. At a room-temperature, the surface leakage current dominates the bulk one starting at mesa diameter less than ≈ 17 μm. As is marked in Section 3.3, the surface of semiconductor like InAs (also bulk InAsSb and InAs/InAsSb T2SLs) exhibits n-type conductivity even when the material is p-doped. After etching, the detector pixels contain exposed side-walls with a p-n^+^ surface junction when absorber is doped on a p-type. Thus, for good performance, such detectors would require passivation.

**Figure 37:**
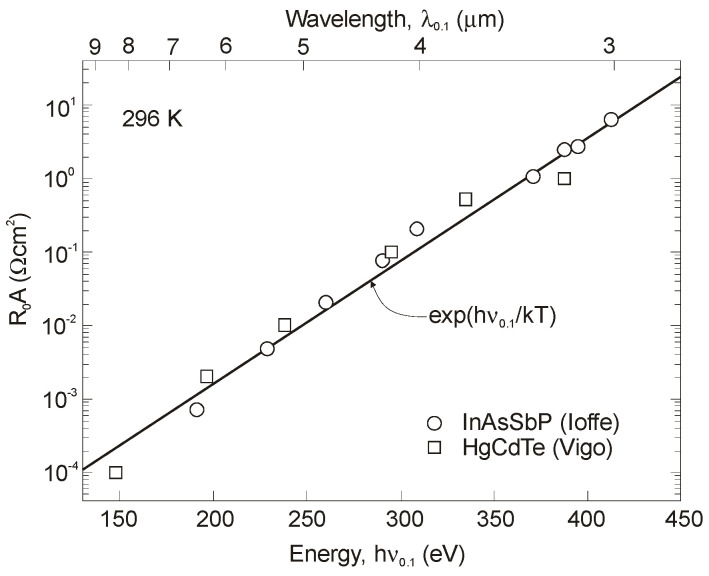
*R_o_A* product in series of InAsSb DH at room temperature (after [127]).

Figure 37 summarizes experimental data of *R_o_A* product versus photon energy for n^+^-InAs/n-InAsSb/p-InAsSbP DH photodiodes in wide wavelength spectral range to 9 μm. The experimental data for InAsSb photodiodes are comparable with those of HgCdTe photodiodes produced by VIGO System. It is assumed that the photon energy in the region of 90% photocurrent drop is being close to the energy gap of photodiode active region. An exponential dependence of *R_o_A* product, approximated by exp(hν_0.1_/kT), shows that the transport properties are determined by the diffusion current and the leakage current flow mechanism is negligible.

**Figure 38:**
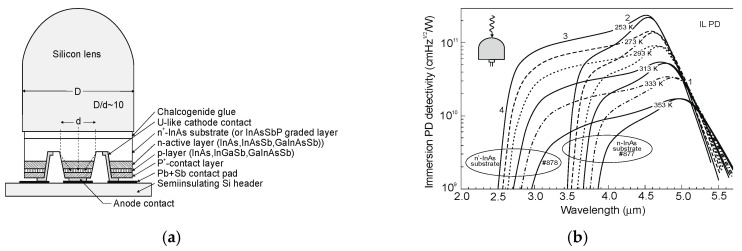
DH InAsSb immersion photodiodes: (**a**) construction of the immersion photodiode (after [121]) and (**b**) detectivity spectra at different temperatures for InAsSb photodiodes with n-InAs and n^+^-InAs substrates (after [123]).

The final construction of immersion photodiode is similar to that shown in Figure 38a [123]. Figure 38b shows the Johnson-limited detectivity at different temperatures of InAsSb DH photodiodes with immersion lenses. As seen from this figure, on the spectral responsivity characteristics, four distinct regions can be distinguished: region of a rapid responsivity decay in the wavelength range from 4.7 μm to 5.5 μm, so called the cut-off region (1), highest responsivity region with a sharp longwave response decline (2), smooth response decline region (3), and finally fast shortwave response decline region, so called cut-on region (4). A short-wave responsivity limit depends on the transparency of the substrate. In the case of a highly doped n^+^-InAs substrate, the BM effect shifts the detector response by 1 µm towards the shorter wavelengths (see difference in short wavelength spectral responsivity between heavily doped-#878 sample and undoped-#877 sample).

**Figure 39:**
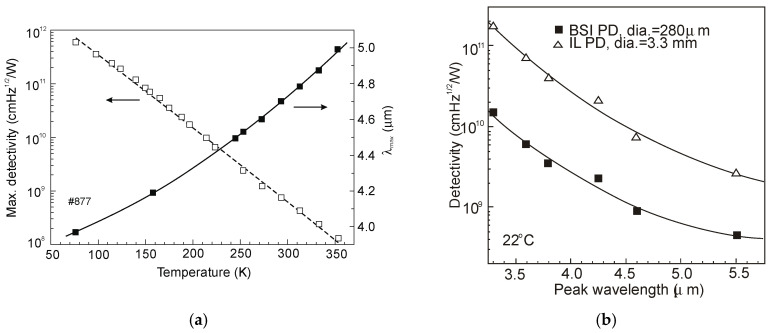
Detectivity of InAsSb DH photodiodes: (**a**) maximum detectivity and peak wavelength versus temperature (after [123]); (**b**) peak detectivity versus peak wavelength of photodiodes without (BSI) and with Si lenses (IL) (after [121]).

Figure 39a summarizes the peak detectivity of photodiodes in dependence of temperature. The next figure, Figure 39b, presents detectivities in dependence on peak wavelength for the flat BSI photodiodes and the immersion lens photodiodes (immersion illuminated—IL). For the immersion lens photodiodes, the peak detectivities are generally about one order of magnitude higher than those for bare chip PDs-see Figure 39b. The photodiodes developed at the Ioffe Institute are superior to the majority published in literature. At the same time, the *R_o_A* products are lower than that given in [8].

**Figure 40:**
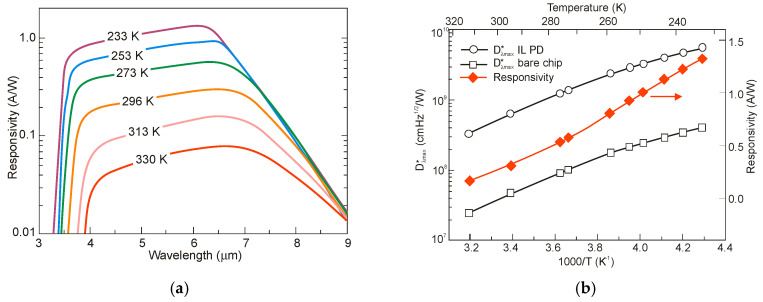
DH InAsSbP/InAs_0.7_Sb_0.3_ p-n photodiodes grown onto InAs substrates: (**a**) photoresponse spectra in the 233–330 K interval, (**b**) maximum detectivity for bare chip (⧠) and immersion lens (○) photodiodes (left scale) and responsivity (right scale,) versus temperature in a 195-µm wide bare chip (after [125]).

More recently, the Ioffe’s research group has demonstrated the InAsSbP/InAs_0.7_Sb_0.3_ DH photodiodes grown onto InAs substrates with peak responsivity about 6.5 μm. Their spectral current responsivity in temperature range between 233 K and 330 K is shown in Figure 40a. The Jonson noise limited detectivity at 233 K, achievable by a 2-stage thermoelectric cooler, is 3.2 × 10^8^ cmHz^1/2^/W at *λ* = 6.5 μm. The use of the integrated with photodiode a hyperhemispherical immersion Si lens (*Ø* = 3.5 mm) increases the D* value to 4.3 × 10^9^ cmHz^1/2^/W (see Figure 40b).

### 5.2. Performance Limits

Despite the promise of the III-V based detectors, HgCdTe remains the highest-performing IR material technology for a number of applications. High-quality InAsSb photodiodes optimized for the 3–5 μm spectral region with performance comparable to HgCdTe have been developed over the last 30 years. However, a long-standing hope that the InAsSb might become a useful material in 8–12 μm spectral band has still not been realized. In comparison with HgCdTe, the main obstacles to achieving this goal are: poor quality crystal structure (there is no ideal III–V substrate/epitaxial combination that is appropriate for LWIR spectral band), poor SRH lifetimes, and relatively high background carrier concentration (lowest at the level of 5 × 10^14^ cm^−3^). Moreover, the observed energy gaps of InAs_1−x_Sb_x_ with middle Sb compositions (close to minimum energy gap (see Figure 4 and Figure 5)) are not inherent and well controlled due to CuPt-type ordering and residual strain effects. The design of buffer layer and its thickness have strong impact on the *R_o_A* product and spectral responsivity [126].

The *R_o_A* product is a commonly encountered figure of merit for a photovoltaic detector and is defined as

(12) $$R_{o}A={\left(\frac{\partial J}{\partial V}\right)}_{V_{b}=0}^{-1},$$

at zero-biased voltage *V_b_* = 0. Here *J* = *I*/*A* is the current density, and *A* is the detector area.

**Figure 41:**
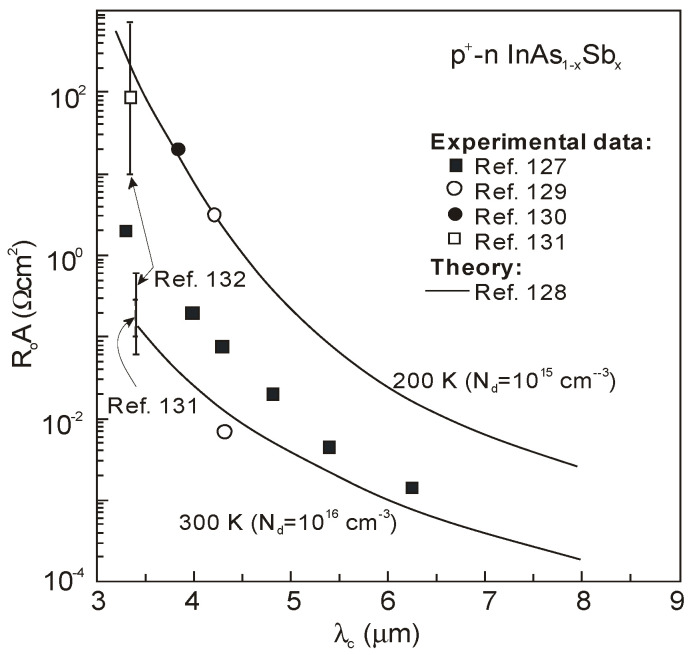
The *R_o_A* product versus *λ_cut-off_* for InAs_1−x_Sb_x_ DH photodiodes at temperatures 200 and 300 K. The solid curves are calculated for two doping concentrations (10^15^ cm^−3^ and 10^16^ cm^−3^) in the active region of photodiode with a thickness of 15 μm; the carrier concentration of 10^18^ cm^−3^ in p^+^-cap layer with thickness equal 1 μm is assumed. The experimental data are taken from [127] (∎), [129] (o), [130] (•), [131] (□), and [132].

Rogalski et al. have reported a theoretical analysis of MWIR InAs_1−x_Sb_x_ (0 ≤ *x* ≤ 0.4) photodiodes with operation extending to the temperature range of 200–300 K [128]. Figure 41 presents a theoretical critical value of the *R_o_A* product for InAsSb DH photodiodes operating at 200 and 300 K and in spectral range between 3 and 8 μm. With the current state of technology, the electron concentration in the undoped absorber is at the level of 10^15^–10^16^ cm^−3^, thus the theoretical calculations have been done for two doping close to the level available in practice. For comparison with theoretical predictions, only limited experimental data published to 1996 is marked. The agreement is satisfactory assuming 15-μm thick base region of photodiode (solid lines). However, as is shown in this figure, the room-temperature experimental results published recently in [127], are located above theoretically predicted line. The main reason of this discrepancy is considerably lower thickness of active region-typically 4 μm. By reducing the photodiode volume in which diffusion current is generated, the corresponding *R_o_A* product increases by a factor of *L_h_*/*d* [3], where *L_d_* is the minority diffusion length and *d* is the thickness of base region.

**Figure 42:**
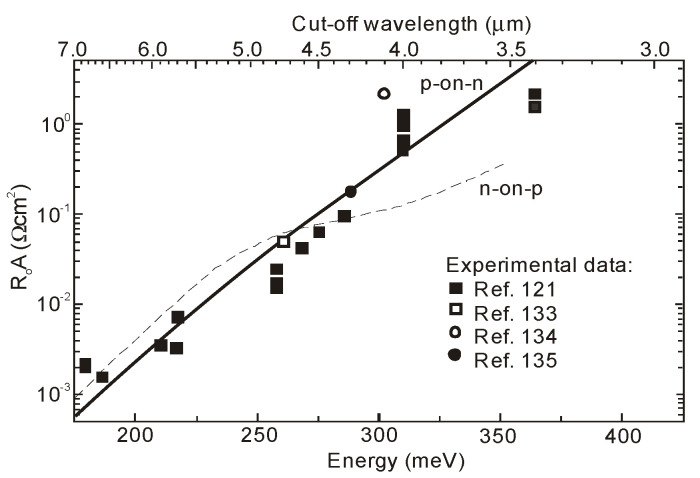
The *R_o_A* product versus *λ_cut-off_* for InAs_1−x_Sb_x_ p-on-n and n-on-p photodiodes at room temperature. The theoretical lines are calculated for doping concentration of 10^16^ cm^−3^ in both p- and n-type 5-µm thick absorber. The gathered experimental data concerns p-on-n photodiodes with n-type active region (after [91]).

More recently, Wróbel et al. have considered the effects of doping profiles on room temperature MWIR InAsSb photodiode parameters (*R_o_A* product and detectivity) [91]. In theoretical estimates a new dependence of the spin-orbit-splitting energy on the Sb molar composition was taken into account [89] -see Figure 17. Figure 42 shows the dependence of the *R_o_A* product on the *λ_cut-off_* for InAs_1−x_Sb_x_ p-on-n and n-on-p photodiodes at 300 K. Electron and hole concentration in a 5-µm thick active regions, respectively in p-on-n and n-on-p photodiode, was assumed at the level of 10^16^ cm^−3^. It is clearly shown that in the photodiode with p-type active region with a Sb composition close to InAs (0 ≤ *x* ≤ 0.15; *λ_cut-off_* < 4.5 µm), Auger S mechanism considerably decreases *R_o_A* product in comparison with the photodiode with n-type absorber, in which Auger S mechanism is not significant. However, for longer wavelengths (*x* ≥ 0.15; *λ_cut-off_* > 4.5 µm), the n-on-p photodiode is more optimal than p-on-n one.

The theoretical relations presented in Figure 42 were also compared with the experimental values for the p-on-n photodiode. A good agreement between both types of data was obtained, even though some experimental points are located above the theoretical line. This can probably be observed if the thickness of the active region is less than the diffusion length of the minority carriers.

Knowing the *R_o_A* product, the normalized detectivity limited by the thermal noise can be calculated using formula

(13) $$D^{*}=\frac{\eta \lambda q}{2hc}{\left(\frac{R_{0}A}{kT}\right)}^{1/2},$$

assuming *QE*, *η* = 0.7.

**Figure 43:**
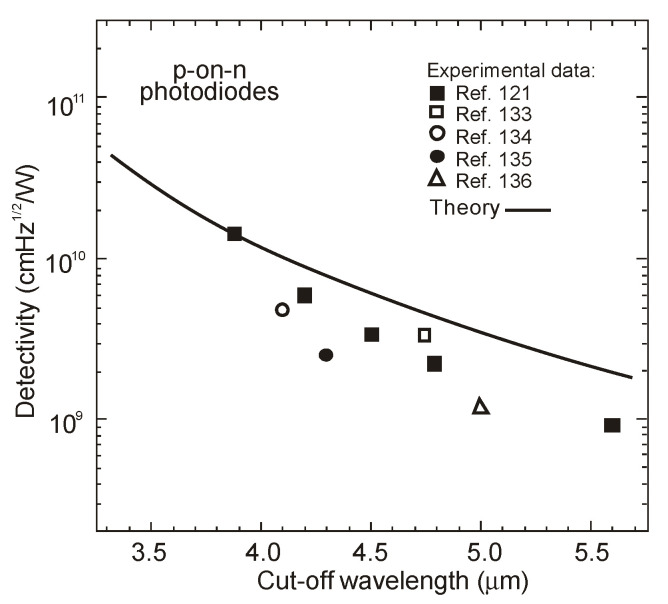
Normalized detectivity versus *λ_cut-off_* for InAs_1−x_Sb_x_ p-on-n and n-on-p photodiodes at room temperature. The theoretical line is calculated for 5-µm thick active region with doping concentration of 10^16^ cm^−3^. The gathered experimental data is taken from literature (after [91,121,133,134,135,136]).

The next figure (Figure 43) shows experimental and theoretical normalized detectivity of p-on-n InAsSb photodiodes operating at room-temperature and different *λ_cut-off_*. The discrepancy between experimental data and theoretical prediction increases versus *λ_cut-off_*. It may be caused by decrease of measured *QE*, while the theoretical was assumed to be constant.

**Figure 44:**
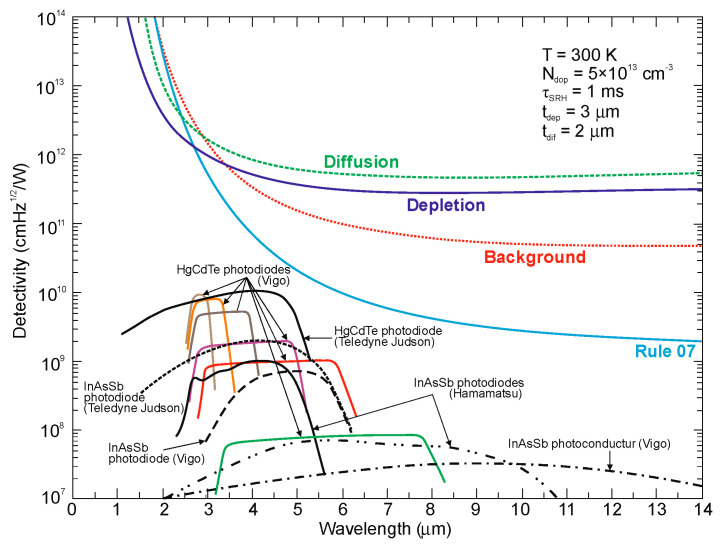
Detectivity on wavelength dependence for the commercially available room-temperature HgCdTe photodiodes and InAsSb photodetectors [137,138,139,140]. The theoretical curves are calculated for P-i-N HOT HgCdTe photodiodes assuming the value of *τ_SRH_* = 1 ms, the absorber doping level of 5 × 10^13^ cm^−3^ and the thickness of active region *t* = 5 µm (*t_dif_*-diffusion thickness, *t_dep_*-depletion thickness) [119].

Figure 44 compares the experimental detectivity values of commercially available different types of InAsSb photodetectors (mainly photodiodes) operating at room temperature with experimentally measured HgCdTe photodiodes and theoretically predicted curves for P-i-N higher operating temperature (HOT) HgCdTe photodiodes. All experimental data gathered in this figure indicates on the sub-BLIP photodetectors’ performance. As is shown, the best quality HOT photodiodes are fabricated by Teledyne Judson Technologies with detectivity about one order below BLIP curve for *λ_cut-off_* ~5 μm.

At present stage of the HgCdTe technology, the “Rule 07” metric (specified in 2007 [118]) is not a proper approach for prediction of the HgCdTe detector and system performance and as a reference benchmark for alternative technologies. The Rule 07 coincides well with theoretically predicted curve for Auger-suppressed p-on-n photodiode with electron concentration in active region ~10^15^ cm^−3^. For sufficiently long SRH carrier lifetime in HgCdTe, which is experimentally supported at doping level below 5 × 10^13^ cm^−3^, the internal P-i-N HgCdTe photodiode current is suppressed and performance is limited by the background radiation [117]. In *Extended Abstracts* of *The 2019 U.S. Workshop on the Physics and Chemistry of II-VI Materials* [132], it was suggested to replace “Rule 07” with “Law 19”. The “Law 19” corresponds exactly with the BLIP curve for room temperature [117]. The internal photodiode current can be several orders of magnitude below “Rule 07” versus *λ_cut-off_* and operating temperature. In this context, the alternative technologies should be considered and evaluated. 

**Figure 45:**
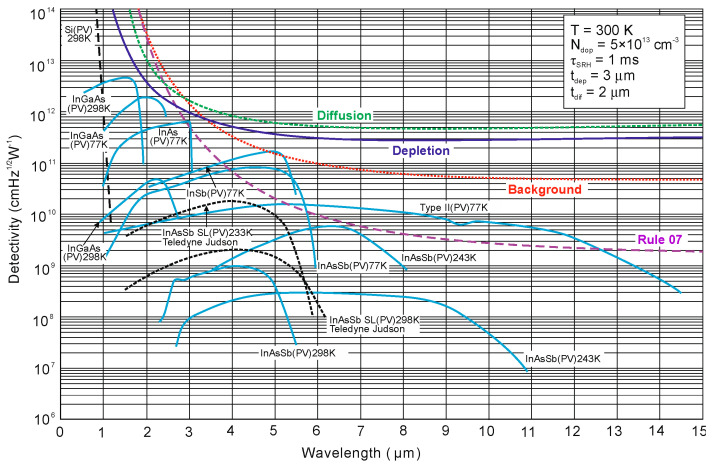
Spectral detectivity curves of commercially available photovoltaic (PV) detectors at different temperatures (after [140]). The theoretical curves are calculated for P-i-N HOT HgCdTe photodiodes assuming the value of *τ_SRH_* = 1 ms, the absorber doping level of 5 × 10^13^ cm^−3^, and the thickness of active region *t* = 5 µm [119].

Figure 44 shows that the potential properties of HOT HgCdTe photodiodes operating in longer wavelength IR range (above 3 μm) guarantee achieving more than order of magnitude higher detectivity (above 10^10^ Jones) in comparison with value predicted by “Rule 07” [119]. The above estimates provide further mobilization for achieving high performance MWIR and LWIR HgCdTe FPAs operating in HOT conditions, at low production costs. In the most optimistic scenario, antimonide/arsenide-based photodetectors can totally supplant the Hg_1−x_Cd_x_Te devices for each spectral range and operating temperature. Particular fabrication efforts provide Hamamatsu with a variety of IR detectors with different spectra response characteristics-see Figure 45.

### 5.3. Barrier Detectors

The first barrier detector was proposed by A.M. White in 1983 [141] as a high impedance photoconductor. Its design was similar to the currently used nBn detector: a thin wide bandgap layer was placed between a bandgap gap n-type absorber region and the same narrow bandgap contact region. A.M. White in his prescient patent also proposed a bias-selectable two-color detector realized and exploited currently in HgCdTe and in the T2SL material systems.

The barrier detector principle of operation is to allow the flow of one carrier type (electron or hole), while blocking the others-hence the term “unipolar barrier”. Thus, the concept of unipolar barrier assumes almost zero offset approximation throughout the heterostructure in one band and a high barrier in the other band. The most popular between different types of unipolar barrier detectors is the nBn detector. 

**Figure 46:**
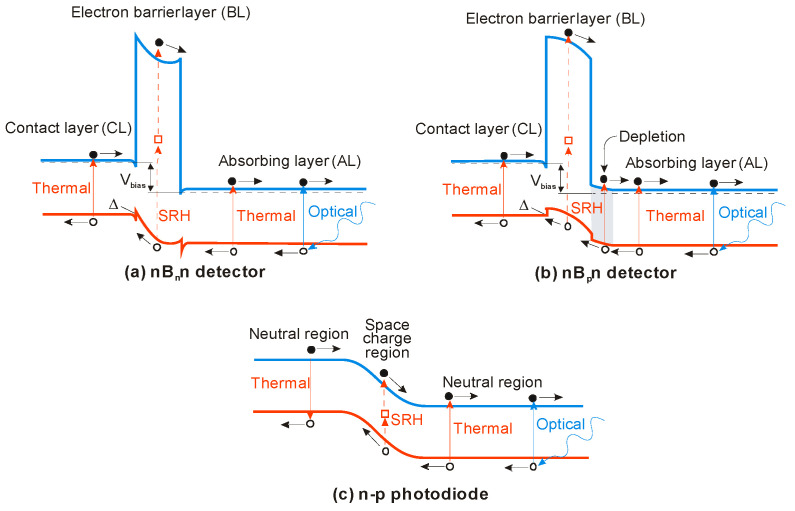
Bandgap diagram of nB_n_n (**a**) and nB_p_n (**b**) barrier detectors (Δ-VBO) and p-n photodiode (**c**).

A typical nBn concept assumes a uniform n-type doping through the heterostructure (see Figure 46a), which is key to maintain a low, diffusion limited dark current. From one side of the unipolar barrier (B) is a narrow-bandgap semiconductor which constitutes the photon-absorbing layer with a thickness comparable to the absorption length of light in the device, typically several microns. The narrow-bandgap semiconductor on the other side of the barrier plays a role of a contact layer for device biasing. Barrier should be located near the minority carrier collector and away from the region of optical absorption. As mentioned, the barrier needs to be carefully engineered. A nearly zero VBO should allow photogenerated holes to flow to the contact (cathode), while large offset in the CB should block majority carrier dark current, reinjected photocurrent. 

The nBn detector can be considered as a hybrid of the p-n photodiode and the photoconductor. The p-n junction (space charge area) (see Figure 46c) is replaced by the barrier, and p contact is replaced by the n-type layer. Due to the absence of majority carrier flow, in this respect it is similar to a photoconductor with unity gain.

The operating principles of the nBn and related detectors have been widely demonstrated in the literature [101,142,143,144,145,146,147,148,149,150]_._ The idea of nBn concept has originated with bulk InAs [142]. However, it is difficult to realize little or no VBO using this material and other bulk IR detector materials such as InSb and HgCdTe. In the middle of first decade of the XXI century, the IR technology has changed after the introduction of 6.1 Å III-V material family and first demonstration of high-performance detectors and FPAs [142,143]. The introduction of broken-gap T2SLs was particularly helpful in the design of unipolar barriers, as they enable any modification of the detector architecture with better control of band edge alignments [151]. The ability to tune the positions of the CB and VB edges independently is easier than in bulk materials.

In general, unipolar barriers architecture is used to increase the photogenerated carriers’ collection efficiency, reduce dark current (associated with SRH processes), and noise without inhibiting photocurrent flow. In particular, the barrier plays a role of self-passivation and serves to reduce the surface leakage current. Other key benefits of uniform doping of nBn detector (n-type barrier, B_n_) is the absence of depletion regions in the narrow-gap absorption layer. It offers a way for materials with relatively poor SRH lifetimes, such as all III-V compounds, to grow on lattice-mismatched substrates such as GaAs, with a reduced penalty of excess of the dark current generated by SRH centers associated with dislocations. 

The papers [144,145] established a criterion for combination of voltage and barrier doping concentration allowing operation with no depletion region in the narrow gap active layer. In some material system, a barrier in the VB is present when the barrier layer is n-type (B_n_), that can significantly impede hole current flow from absorber to the contact layer. In such situation, a relatively high voltage (so called “turn-on voltage”) is required to overcome. A p-type doping of the barrier, B_p_, helps to reduce the barrier in the VB (see Figure 46b) but rather a potential well for holes in the VB which does not impede hole transport between the absorption layer and the contact layer. However, in the last case, a p-type barrier layer inherently causes depletion regions to form in the narrow-gap absorption layer for all voltages, what should be avoided (depletion regions cause excessive G-R dark currents).

Detailed growth procedures and device characterization of MWIR detectors based on InAsSb/AlAsSb material system were published in several papers, e.g., References [144,145,147,152,153]. The principal layers of the device structures were n-type doped and consisted of a thick InAs_1−x_Sb_x_ absorption layer (1.5–3 μm), a thin AlAs_1−x_Sb_x_ barrier layer (0.2–0.35 μm), and a thin (0.2–0.3 μm) InAs_1−x_Sb_x_ contact layer. The bottom contact layer was highly doped. InAsSb/AlAsSb nBn structures were grown on either GaAs(100) or GaSb(100) substrates by using MBE technique [153]. The n-type doping was usually obtained by either Si or Te elements. The remaining structures were grown directly onto lattice-matched GaSb (100) substrates, while lattice-mismatched GaAs (100) substrates were used, structures were grown on a 4-μm thick GaSb buffer layer.

**Figure 47:**
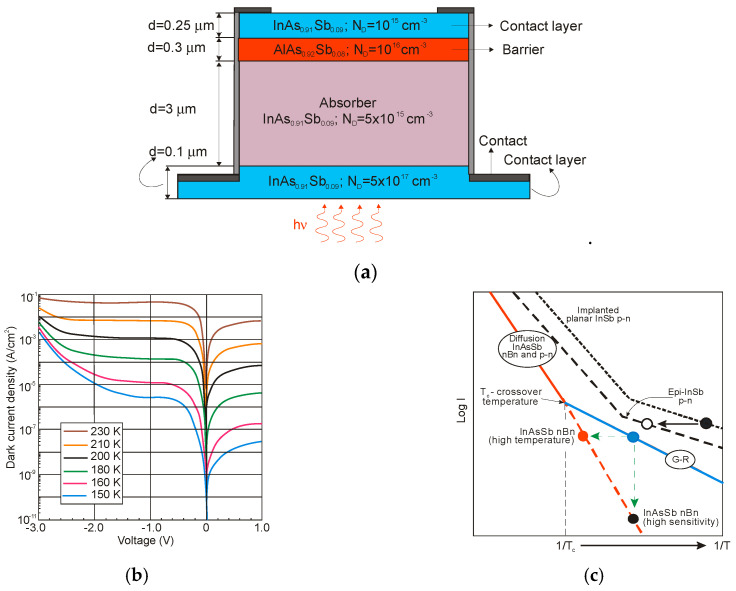
InAsSb/AlAsSb nBn MWIR detector: (**a**) the device structure ([154]), (**b**) dark current density versus voltage for InAs_0.805_Sb_0.195_ for selected temperatures 4096 (18 µm pitch) detectors (*λ_cut-off_* ≈ 4.9 µm at 150 K) tied together in parallel (after [155]), and (**c**) schematic Arrhenius plot of temperature dependence of the dark current in two type InSb p-n junctions (made by ion-implantation and MBE grown (dashed line). The diffusion and G-R limited portions of the InAsSb curves are also labeled.

Figure 47a shows an example of such an nBn structure that was theoretically investigated by Martyniuk and Rogalski [154]. The alloy compositions of *x* = 0.09 to 0.2 for the InAs_1−x_Sb_x_ absorber layers provided the *λ_cut-off_* wavelengths of ~4.1 to ~5.0 μm at 150 K, respectively. The current-voltage characteristics were taken from [155]. The dark current density for nBn InAs_0.805_Sb_0.195_ structure was 1.0 × 10^−3^ A/cm^2^ at 200 K and 3.0 × 10^−6^ A/cm^2^ at 150 K. 

In nBn InAsSb detector, the depletion current is totally suppressed (see Figure 47c) because there is no depletion region. In the low-temperature region, for temperatures below *T_c_*, the nBn detector exhibits a higher signal-to-noise ratio in comparison with the conventional p-n diode operating at the same temperature, or otherwise the nBn detector can operate at a higher temperature than a conventional p-n diode with the same value of dark current. InAsSb p-n photodiode is characterized by a higher crossover temperature, *T_c_*, between diffusion and G-R contribution than InSb, which results from the larger bandgap of InAsSb than InSb (only in a certain range of Sb molar composition, for InAsSb close to InAs).

In InAsSb nBn detector (n-type absorber), the dark current density limited by the Auger 1 and SRH mechanisms can be calculated as

(14) $$J_{dark}=\frac{qN_{d}t}{2\tau_{A1}^{i}}+\frac{qn_{i}^{2}t}{\left(N_{d}\;+\;\;n_{i}\right)\tau_{SRH}},$$

where *N_d_* and *t* are the doping and thickness of active region, respectively. The influence of G-R current associated with a depletion region (*J_dep_* = *qn_i_w*/*τ_SRH_*, were *w* is the depletion width) can be omitted.

**Figure 48:**
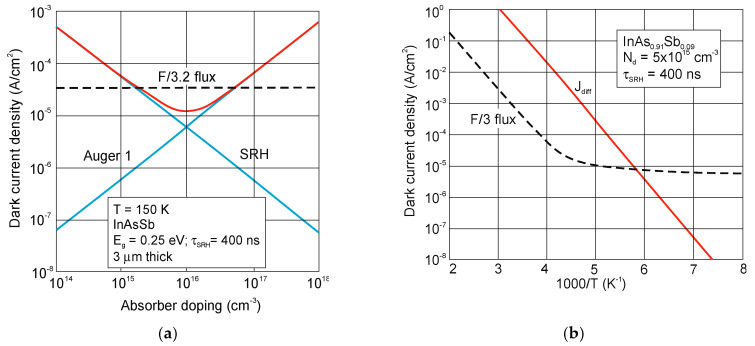
Dark current density for InAsSb nBn absorber (*τ_SRH_* = 400 ns, *t* = 3 µm): (**a**) versus doping concentration, and (**b**) versus inverse temperature for a donor doping 5 × 10^15^ cm^−3^ (after [78]).

According to Equation (14), the Auger 1 generation varies as *N_d_*, whereas the SRH generation varies as 1/*N_d_*. It follows that that the diffusion dark current mostly depends on absorber doping concentration and on value of SRH lifetime. Kinch has modeled the diffusion current in an InAsSb absorber versus doping concentration at 150 K (assuming *τ_SRH_* = 400 ns (see Figure 19) and an absorber thickness of 3 μm). As is shown in Figure 48a, the optimum doping is 10^16^ cm^−3^. Next, Figure 48b shows the dark current density for InAs_0.91_Sb_0.09_ absorber versus inverse temperature for doping concentration 5 × 10^15^ cm^−3^. This figure shows that the dark current is equal to the *f*/3 background flux at temperature of ~175 K. At 150 K, the dark current density is 2 × 10^−7^ A/cm^2^, in agreement with experimental nBn InAsSb data [156]. In this way, the nBn InAsSb detector operating with *f*/3 optics reaches BLIP for *T* > 160 K (temperature two times higher than traditional InSb).

**Figure 49:**
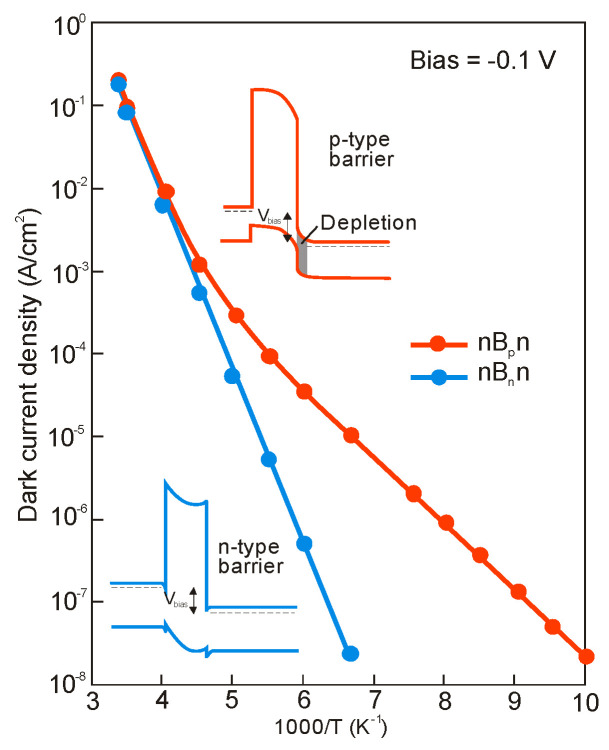
Dark current density vs. temperature for two identical InAsSb/AlSbAs nBn devices with opposite barrier doping. Active layer bandgap wavelength is 4.1 µm at 150 K (after [150]).

Interesting difference in dark current density versus temperature is shown in Figure 49 for two nominally identical nBn devices, each operating at a bias of −0.1 V. The devices differ only in the doping of the barrier layer. The nB_n_n device with a uniform n-type doping through the entire structure exhibits a single straight line, typical for the diffusion limited behavior. The nB_p_n device, with a barrier doped opposite to the absorber, exhibits two-slope behavior, typical for the diffusion limited mode at high temperatures and the G-R limited behavior at low temperatures. As is shown, the dark current density at 150 K is more than two orders of magnitude greater for the device with the p-type due to the presence of the depletion region. It appears that for a typical *QE* of 70% at *f*/3 optics, the BLIP temperature is about 140 K compared with ~175 K for the detector with the n-type barrier.

Over present-day IR technologies, InAsSb-based barrier detectors offer numerous advantages, such as reduced depletion and surface leakage currents, normal-incidence absorption. However, the promise of the excellent performance of these detectors has yet to be practically realized. Martyniuk and Rogalski have predicted the temperature dependencies of dark currents of different types of IR detectors, including InAsSb nBn detectors, optimized at a *λ_cut-off_* = 5 μm [157]. 

**Figure 50:**
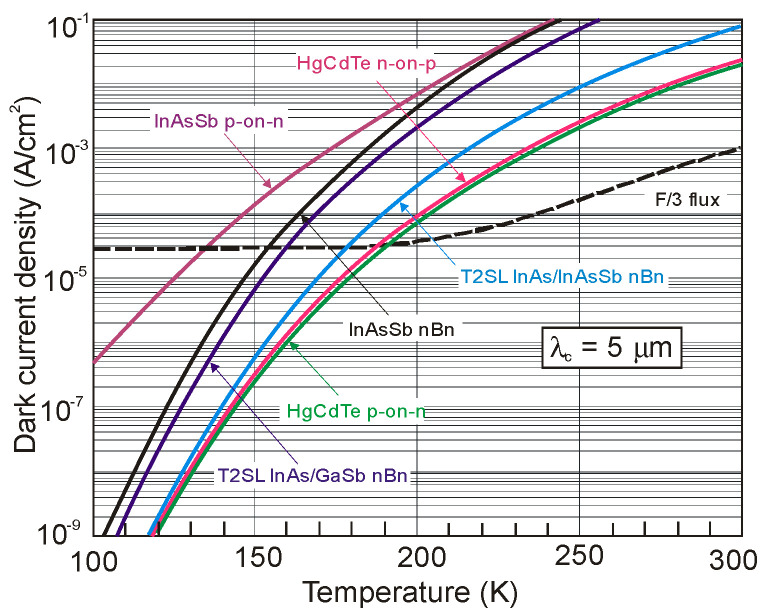
Dark current density versus temperature for InAsSb p-on-n photodiode and nBn detector, T2SL nBn detectors, and HgCdTe photodiodes with *λ_cut-off_* = 5 µm.

The advantages of the nBn structure, compared to the InAsSb photodiode with the built-in depletion region, are clearly visible. The nBn structure enables operation at much higher temperatures while maintaining the same dark current values (see Figure 50). However, still these values are not comparable to the HgCdTe photodiodes with p-on-n or even n-on-p design. More comparable to HgCdTe photodiodes is T2SL InAs/InAsSb nBn detector. MWIR HgCdTe systems operating at *f*/3 optics at 160 K are commercially available.

Early efforts in development of InAsSb barriers detectors focused on lattice-matched structures to GaSb substrates with *λ_cut-off_* ~ 4.1 μm. Different strategies were attempted to extend the *λ_cut-off_* above 5 μm including various metamorphic buffer layers and InSb interface ML into the InAsSb [158,159]. It appears that the activation energies estimated from temperature dependence of dark currents is higher than the bandgap of InAsSb absorbers, which is explained by location of the accumulation layer between the barrier and absorber due to band bending [160]. Some barrier detectors also exhibits an increase in the turn-on bias versus temperature as a result of the Fermi level position changes in low-doped or unintentionally doped absorbers. At higher temperature, a higher voltage is needed to deplete the barrier-absorber interface region [161].

Lin et al. [42] have described bulk InAsSb barrier detectors with a *λ*_cut-off_ about 10 μm at 77 K. The estimated InAs_0.60_Sb_0.40_ absorber parameters at 77 K are as follows: the hole mobility of 10^3^ cm^2^/Vs, the minority hole lifetime of 185 ns, and the diffusion length of 9 μm. The current-voltage characteristics have been influenced by G-R component associated with a depletion region adjacent to the barrier, as well as probably tunneling components. To reach diffusion-limited dark current, the VBO associated with the heterointerfaces must be eliminated.

**Figure 51:**
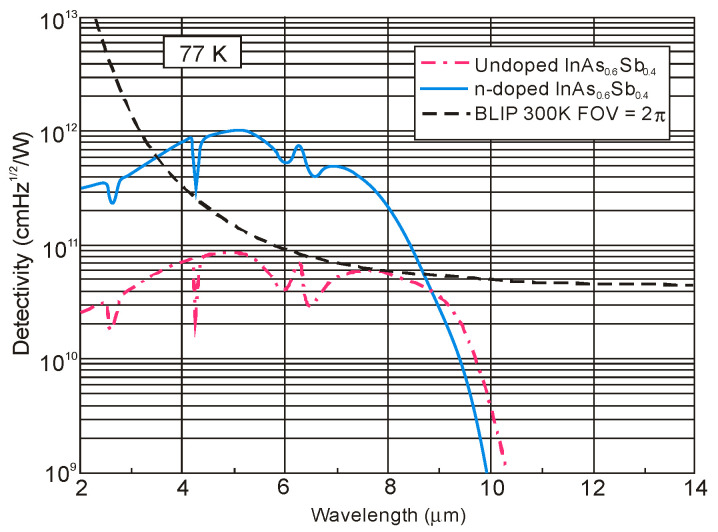
Spectral detectivity of barrier detectors with 1 µm thick InAs_0.6_Sb_0.4_ absorbers at *T* = 77 K. Dashed and solid lines represent devices with undoped and n-type doped absorbers, respectively. Dotted line shows the 300 K BLIP limit for a 2π FOV (after [42]).

The spectral detectivity curves of LWIR nBn InAsSb detector, with two absorber’s doping levels, are shown in Figure 51. The demonstrated detectivity of 2 × 10^11^ cmHz^1/2^/W at 2π FOV and wavelength of 8 μm was estimated in spite of the significant shift towards shorter wavelengths (BM shift) with doping. For 1 μm thick InAs_0.60_Sb_0.40_ absorber at *λ* = 8 μm, an absorption coefficient was estimated at 3 × 10^3^ cm^−1^, what implies *QE* = 22%. The *QE* increases with bias, until it reaches a constant level for −0.4 V and with thickness of absorption layer (to 40% for 3-μm thick absorber). Generally, due to poor quality crystal structure, the performance of LWIR bulk InAsSb detectors are considerably inferior in comparison with HgCdTe photodiodes. 

## 6. InAs/InAsSb Superlattice Detectors

**Figure 52:**
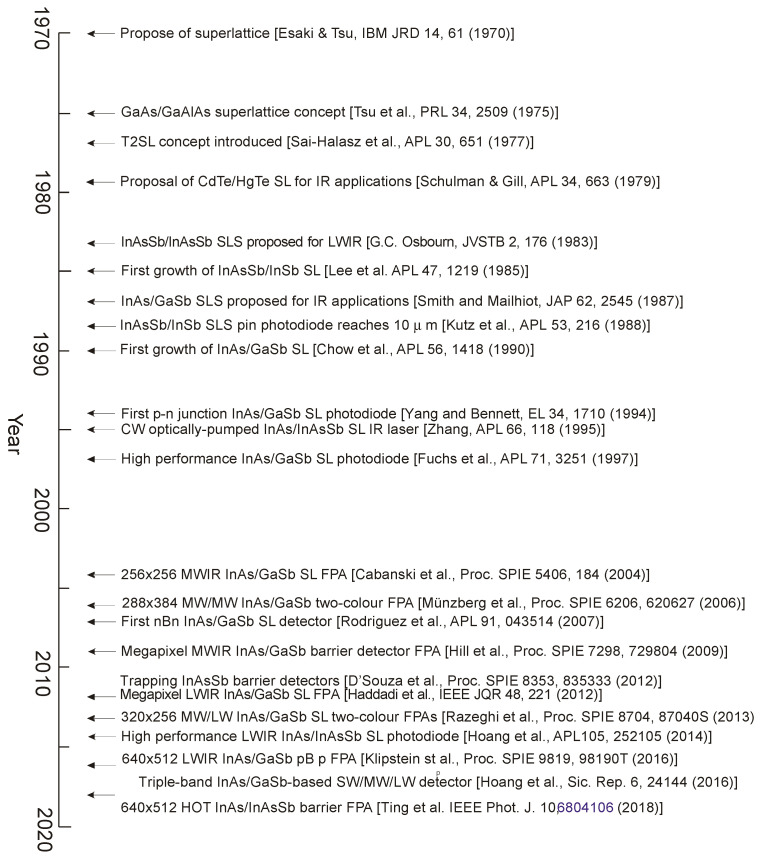
Roadmap development of T2SL IR photodetectors including InAs/InAsSb SL photodiodes and barrier detectors.

Development of the T2SLs has more than forty years history. Figure 52 gives it a short description in the form of a timeline, including InAs/InAsSb SLs. The development of InAs/InAsSb SLs for IR detector applications is described in detail in several papers [19,21,159,162]. It has a long and interesting history that predates the InAs/GaSb SL detectors. Up till 2010 this material system was much less explored than the T2SLs InAs/GaSb. In the last decade, a rapid progress in their fabrication is made due to some defect tolerance and its robust material properties. In general, the growth of the InAs/InAsSb T2SLs is easier compared to the InAs/GaSb ones and InAs/InAsSb SLs have longer minority carrier lifetimes (see Section 4.3). On the other hand, its spectral tuneability is somewhat smaller and the InAs/InAsSb T2SLs has weaker optical absorption. In addition, vertical hole transport properties, especially in the LWIR range, are more challenging. 

### 6.1. Performance Limits

The InAs/GaSb SL detectors are most commonly designed with p-type absorbers, while in the case of InAs/InAsSb SL detectors, it is better to use an n-type absorber due to their surface properties. In InAs/InAsSb SLs, the surface Fermi level is pinned above the CB minimum, resulting in a degenerate n-type surface. Thus, for the n-type absorber no special surface treatment is required since the surface band-bending potential repels the minority carriers (holes) away from the surface. In a p-type absorber, the minority electrons are attracted to the n-type surface contributing to the surface leakage current. In addition, the depletion region in the surface p-n^+^ junction is a source of both G-R and tunneling dark currents.

Summarizing the above discussion-choosing between the two types of SLs, InAs/GaSb and InAs/InAsSb, it is necessary to take into account the *λ_cut-off_*, the ease of growth technology and processing capabilities (including passivation). In general, the InAs/InAsSb SLs are more effective for implementation in the MWIR range, where they could have an advantage over the InAs/GaSb ones.

**Figure 53:**
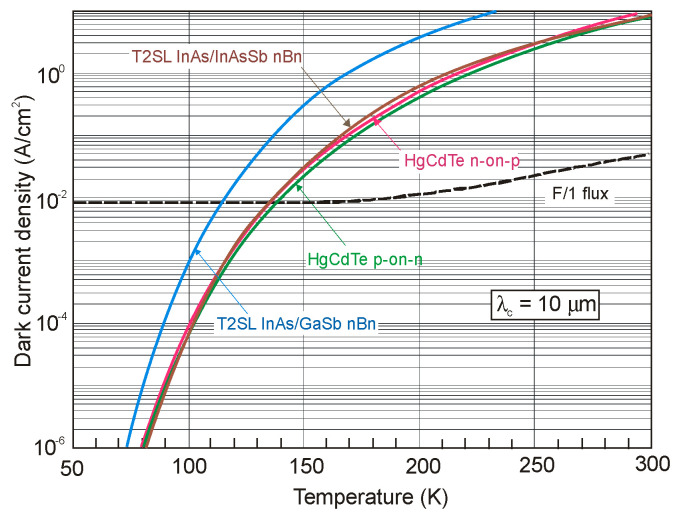
Dark current density versus temperature for T2SL nBn detectors and HgCdTe photodiodes with a *λ_cut-off_* = 10 µm.

Figure 50 and Figure 53 compare the predicted dependence of dark current density on operating temperature for different material systems including InAs/InAsSb SLs with *λ_cut-off_* of 5 and 10 µm. The LWIR InAs/GaSb T2SL materials have larger dark current densities than HgCdTe. Their inferior parameters appear to result from a relatively high background concentrations (about 10^15^ cm^−3^) and a shorter minority carrier lifetime. The BLIP performance with *f/1* optics for InAs/InAsSb nBn detector is achieved at about 130 K and is close to HgCdTe photodiode.

**Figure 54:**
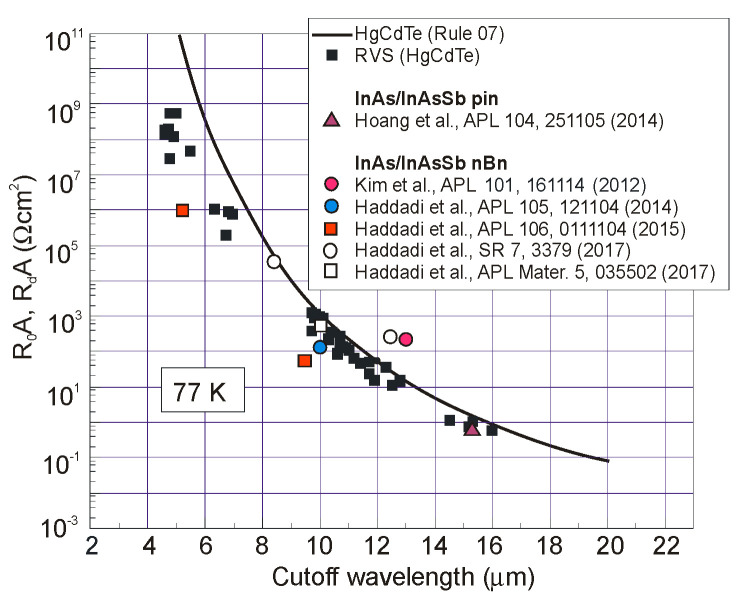
*R_o_A* and *R_d_A* product of nonbarrier and barrier T2SL InAs/InAsSb detectors on *λ_cut-off_* wavelength dependence compared to experimental data and trend line (“Rule 07”) for HgCdTe photodiodes at 77 K. The experimental data for InAs/InAsSb SLs devices are taken from [163,164,165,166,167,168].

Figure 54 compares the *R_o_A* and *R_d_A* values for InAs/InAsSb SL and HgCdTe devices operating in a wide IR spectral range. For good photoresponse of the nBn detectors, the devices must be biased typically in the range above −100 mV. Thus, the *R_d_A* product is used instead of the *R_o_A*, where *R_d_* is the dynamic resistance at non-zero bias voltage. The solid line is the trend line calculated with Teledyne empirical model (“Rule 07” [118]) for P-on-n HgCdTe structures. The HgCdTe experimental data are given for photodiodes fabricated by Raytheon Vision Systems (RVS). As we can see, the upper results for InAs/InAsSb SL detectors rival that of practical HgCdTe devices.

**Figure 55:**
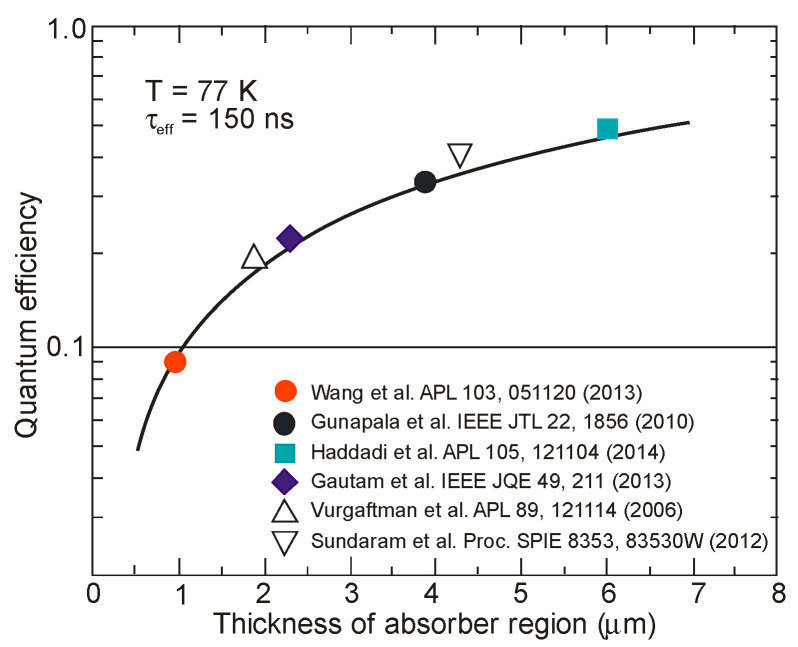
*QE* versus thickness of active region for InAs/InAs_1−x_Sb_x_ T2SL photodetectors at 77 K. The theoretical prediction (solid line) is compared with the experimental *QE* reported by different research groups as indicated inside the figure (adapted after [169]).

In the case of InAs/InAsSb SL barrier detectors having a *λ_cut-off_* ~12 µm, the values of *R_o_A* > 10^2^ cm^2^ at 77 K are measured. These values are higher than those measured for HgCdTe photodiodes. Generally, however, the InAs/InAsSb SL detectors are characterized by lower *QE* ~ 40–50% (see Figure 55) than HgCdTe photodiodes (>70%), mainly due to lower absorption coefficient.

The main technological challenge for the fabrication of SL-based devices is the growth of high-quality active region with sufficient thickness to achieve satisfactory *QE*. For InAs/InAsSb photodetectors, 5 µm thick absorber is sufficient to provide high enough *QE*. It may provide *QE* about 50%, due to a longer effective lifetime and diffusion length, in comparison with InAs/GaSb SLs. Figure 54 shows the theoretical *QE* of InAs/InAsSb T2SL detectors versus the absorber thickness, calculated for the effective carrier lifetime of 150 ns [169]. Table 4 summarizes reports of InAs/InAsSb-based SL IR detector results.

**Figure 56:**
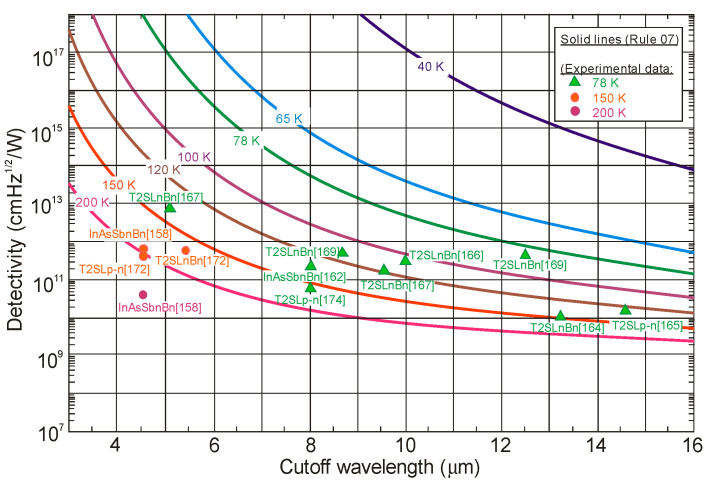
The simulated detectivity of the P-on-n HgCdTe detectors (Rule 07, *QE* = 70%) versus *λ_cut-off_* and temperature. The experimental data is taken with different sources (as marked) for InAs/InAsSb T2SL photodetectors operating at 78 K, 150 K, and 200 K.

Figure 56 shows the comparison of the simulated detectivity of P-on-n HgCdTe photodiodes versus *λ_cut-off_* and operating temperature with the experimental data T2SL InAs/InAsSb photodetectors operating at 78 K, 150 K and 200 K. The solid lines present theoretical thermal limited detectivity for HgCdTe photodiodes, modeled using the “Rule 07” dark current density. This figure indicates that the measured thermally limited detectivity of T2SLs InAs/InAsSb photodetectors exhibits lower performance in comparison to the HgCdTe photodiodes not even reaching the HgCdTe level. That is mainly related to the fairly high background concentrations (~1 × 10^15^ cm^−3^) and a lower absorption coefficient and in consequence lower *QE*. Improvement in those two fundamental parameters seems to be critical to reach the theoretically predicted T2SLs photodiodes performance.

### 6.2. Superlattice Detector Structures

The majority of the T2SL devices are grown by MBE being expensive for mass production, while the recent interest in T2SLs growth by MOCVD is observed which is related to the low-cost and versatile production guaranteed by that technique [173,174].

In the last decade, the particular contribution in development of T2SLs InAs/InAsSb detectors and FPAs has given two research groups; group at Jet Propulsion Laboratory (JPL) [107,158,159,161,171,172] and the Razeghi group from Northwestern University [164,165,166,167,168,173,174]. Both groups have demonstrated the versatility of the T2SLs InAs/InAsSb with operating *λ_cut-off_* ranging from 2 to 14 μm and beyond. In this way, SLs material expends the limited spectral range covered by traditional bulk III-V IR detectors based on InGaAs and InSb.

Table 4 indicates that the T2SLs InAs/InAsSb detectors are fabricated mainly in two configurations: p-n junction photodiodes (impurity diffusion [178], MBE [164], MOCVD [173,174]) and nBn barrier detectors (mainly MBE). The T2SL InAs/InAsSb photodiodes, as shown in Figure 34, are mainly constructed on P-i-N heterostructures. The low minority carrier concentration in the high bandgap materials allows for the suppression of diffusion dark current and reaching higher *R_d_A* product and detectivity.

In comparison with the p-n junction T2SLs InAs/InAsSb photodiodes, SLs IR barrier devices are more effective (see Figure 50 and Figure 53). As is described in Section 5, the unipolar barrier structures allow reducing G-R dark current by suppressing SRH mechanism and surface leakage. That is particularly useful for the p-n devices based on III-V materials, inherently exhibiting excessive depletion dark current and suffering from the lack of proper surface passivation.

**Figure 57:**
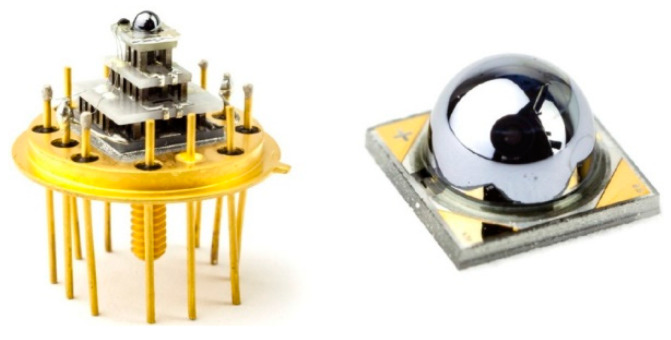
The photodetector assembled on the 3-stage Peltier thermoelectrical cooler and GaAs immersion microlens. The improvement in detectivity reached by hyperhemispherical microlens is proportional to $n_{r}^{2}$, where *n_r_* is the GaAs refractive index (after [180]).

InAsSb-based bulk and SLs photodetector performance has been increased by the monolithically integrated microlenses based on GaAs/GaSb substrates [180,181]. The hemispherical or hyperhemispherical microlenses increase the effective detector’s optical area by $n_{r}^{2}$ of $n_{r}^{4}$, respectively, where *n_r_* is the index of refraction of the lenses [182]. The detectivity gain achieved with hyperhemispherical immersion is $n_{r}^{2}$. Since the refractive index of GaAs is 3.4, a factor of 10 improvements in the detectivity is observed for n-type and p-type InAsSb absorbers [180]. Figure 57 shows the immersion lens formed from GaAs substrates using numerically controlled micromachining.

**Figure 58:**
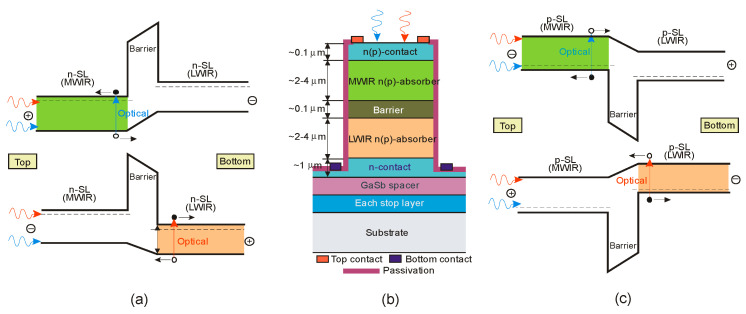
Schematic of nBn (**a**) and pBp (**c**) T2SL dual-band detector operations under forward and reverse bias. Schematic view of dual-band MWIR/LWIR detector architecture is shown in centre (**b**).

The nBn (pBp) detector design is also useful for the realization of dual-band detection capabilities shown schematically in Figure 58. The dual-band barrier structures could be easily fabricated due to the highly developed III-V materials growth techniques. Typical dual-band, barrier T2SLs detector consist of the two narrow bandgap absorbers optimized for the two different IR spectral regions being separated by ~0.1 μm thick wide bandgap barrier for majority carriers (no barrier for minority carriers is assumed). That architecture blocks the majority carrier current between the two electrodes by the large energy offset, while there is no barrier for photo-generated minority carriers. Both barriers should be characterized by negligibly small VB or CB discontinuity with respect to both n-type (*p*-type) doped active regions. The absorbers (channels) are addressed consequently by the polarity of the applied voltage. Detector with pBp design exhibits faster operation with lower integration times, due to the higher minority carrier mobility (electrons).

Krishna et al. have demonstrated two-color barrier detectors based on nBn or pBp InAs/GaSb SL architectures [183,184]. Furthermore, their improvement in InAs/InAsSb two-band SL fabrication was demonstrated in [166,168]. 

#### 6.2.1. Photodiodes

Both MWIR [174,185] and LWIR [164,173] T2SLs InAs/InAsSb photodiodes have been demonstrated. The experimentally measured dark current density of MWIR photodiodes with a *λ_cut-off_* = 5.4 μm at 77K, was found larger than T2SLs InAs/GaSb detectors [186]. This was related to the higher probability of the carrier tunneling due to reduced VBO and CBO in T2SLs InAs/InAsSb.

The better quality LWIR T2SLs InAs/InAsSb photodiodes have been demonstrated by the Razeghi group [164]. Even if the Sb composition increases (*x_Sb_* = 0.43), the material quality is still high leading to the detector’s high performance. The absorber *λ_cut-off_* depends on the VB level in the InAsSb layer being directly related to the Sb composition. The presented samples were grown by MBE on Te-doped (001) GaSb substrate. The analyzed device is built of 0.5-μm InAsSb buffer layer, a 0.5-μm bottom Si-doped n-type contact (*N_D_* ~10^18^ cm^−3^), a 0.5-μm thick n-type barrier, a 2.3-μm Be-doped p-type active region (*N_A_* ~10^15^ cm^−3^), and a 0.5-μm top p-type (*N_A_* ~10^18^ cm^−3^). Finally, the structure was capped with a 200-nm p-type GaSb layer. 

**Figure 59:**
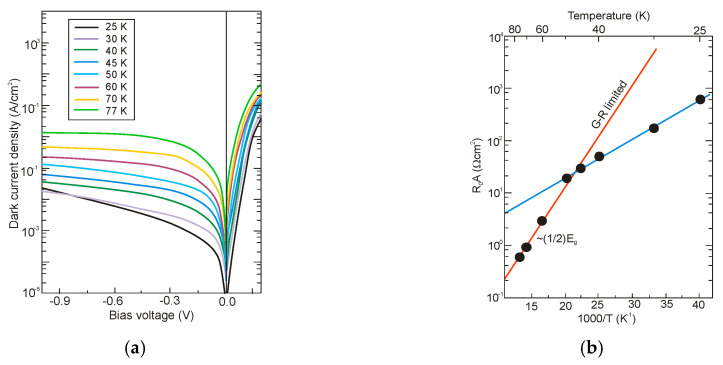
Dark electrical characteristics of LWIR InAs/InAsSb SL photodiodes: (**a**) current-voltage characteristics versus temperatures; (**b**) *R_o_A* versus temperature (after [164]).

Figure 59 shows the electrical characteristics of the LWIR InAs/InAsSb SL photodiode within temperature range from 25 to 77 K. At 77K the *R_o_A* product is 0.84 Ωcm^2^ indicating lower value in comparison with InAs/GaSb counterpart. For *T* > 50 K, the diode exhibits an Arrhenius type behavior with related activation energy, *E_a_* = 39 meV meeting the relation *E_a_* ~*E_g_*/2 of the absorber (~80 meV for 15 μm) indicating on the active region G-R current as the limiting mechanism. For *T* < 50 K, the *R*_o_A product does not follow the trend line and is less sensitive versus temperature. This behavior indicates on the other mechanisms either the tunneling or surface leakage at this range of temperature.

**Figure 60:**
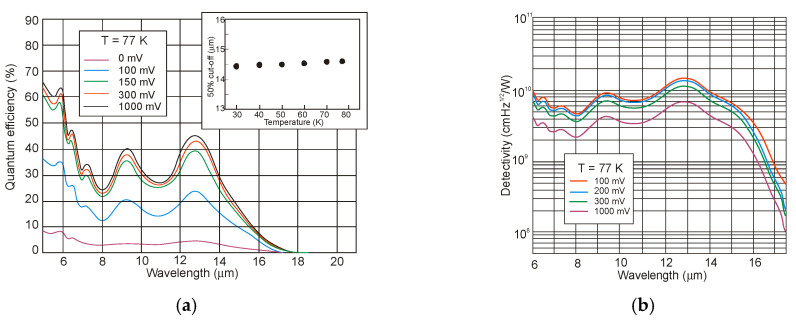
Spectral characteristics of LWIR T2SLs InAs/InAsSb photodiode: (**a**) *QE* spectrum with different applied bias at 77 K. Inset: 50% *λ_cut-off_* versus temperature; (**b**) the calculated shot noise and Johnson noise limited D* at 77K for different applied bias (after [164]).

The spectral characteristics of photodiode are presented in Figure 60. At 77 K, the sample exhibited a 100% *λ_cut-off_* = 17 µm and 50% *λ_cut-off_* = 14.6 µm. High *QE* is reached when biased >150 mV and saturates at 300 mV where the peak current responsivity reaches 4.8 A/W, corresponding to a *QE* of 46% for a 2.3 µm thick absorber. Detectivity shown in Figure 60b, limited by shot noise and Johnson noise, is estimated using equation

(15) $$D^{*}=R_{i}{\left(2qJ_{dark}+\frac{4kT}{RA}\right)}^{-1/2},$$

where *R_i_* is the device responsivity.

The effective passivation of T2SLs InAs/InAsSb photodiodes is in a very early stage of development. Usually, photodiodes are not passivated. The simplest passivation is based on the common dielectric insulators deposited onto exposed surface of the device, utilized in the silicon industry (such as an oxide or nitride of silicon). In the case of T2SLs InAs/InAsSb photodiodes, the passivation challenges are caused by the well-known surface accumulation of InAsSb. Sulphur anodic was found to be more effective passivant for p^+^B_p_nn^+^ detector structure compared to fluoride and chloride anodic films [186]. In [187] the dark current suppression was observed in 8-μm *λ_cut-off_* T2SLs InAs/InAsSb structure with combination of a double-electron barrier and SiO_2_ passivation. In addition, the effect of hydrogenation could be taken into account for passivation of bulk defects in MWIR T2SLs InAs/InAsSb [188].

#### 6.2.2. Barrier Detectors

Klipstein et al. [189] proposed the division of the barrier detectors into two groups: XB_n_n and XB_p_p, where X is a contact layer where doping, material, or both can be varied. Usually, however, the most popular are four types of SLs barrier detectors schematically described in Table 5.

**Figure 61:**
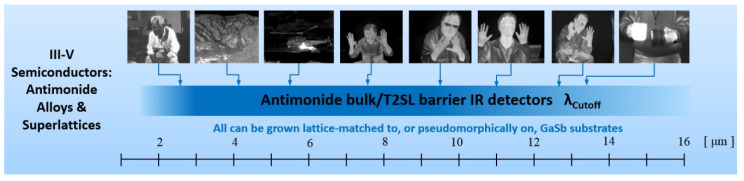
Images from JPL antimonide bulk and T2SLs barrier FPAs with *λ_cut-off_* in wide IR range (after [159]).

Main activity in development of the T2SLs InAs/InAsSb barrier detectors is directed to nBn devices operating in MWIR spectral range at much higher temperatures than InSb photodiodes. Generally, however, these detectors can be optimized for wider spectral range up to VLWIR [191]. The unipolar hole barriers are only available for T2SLs InAs/GaSb and mostly for LWIR. In addition, there is no good hole barrier for T2SLs InAs/InAsSb. Figure 61 shows same images taken with JPL T2SLs arrays in wide IR spectral range. In this section the representative examples of barrier detector performance are presented.

The JPL nBn device structure grown by MBE on GaSb substrate is similar to that shown in Figure 47a. The top/bottom contacts and active layers consist of 16/20 and 525 periods of (37Å, 13Å) T2SLs InAs/InAs_0.66_Sb_0.34_, respectively. As a unipolar barrier located between the top contact and absorber, 120-nm thick AlAs_0.085_Sb_0.915_ Be-doped to 10^15^ cm^−3^ is used. The top contact and active layers are n.i.d, while the bottom contact is Te-doped to 10^17^ cm^−3^.

**Figure 62:**
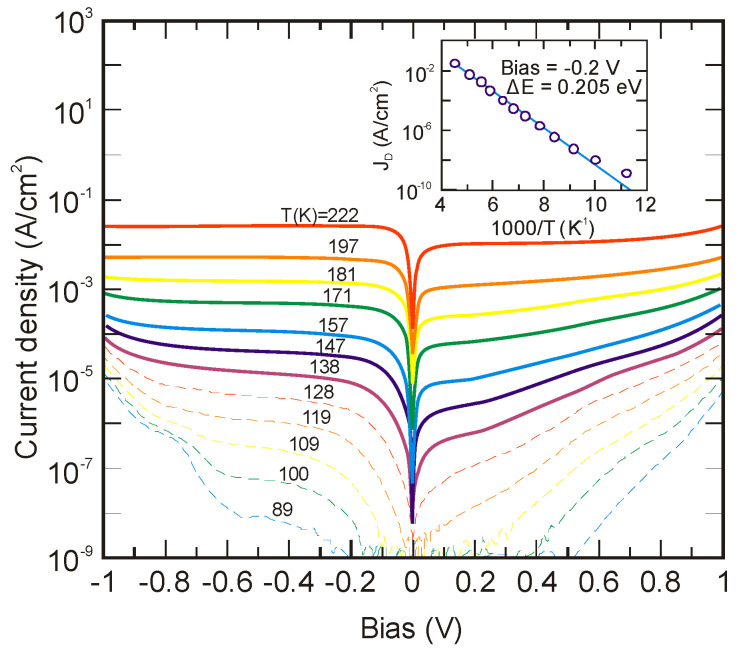
Dark current density versus voltage, for temperatures 89–222 K. The inset presents the dark current temperature dependence −0.2 V (after [171]).

The experimentally measured dark current density versus applied voltage for temperature range between 89 K and 222 K is shown in Figure 62. The inset shows an Arrhenius plot of dark current under bias −0.2 V with activation energy of 0.205 eV. The dark current density at 157 K and bias voltage of −0.2 V is 9.6 × 10^−5^ A/cm^2^, which is ~4.5 higher than “Rule 07” for *λ_cut-off_* = 5.4 μm. 

**Figure 63:**
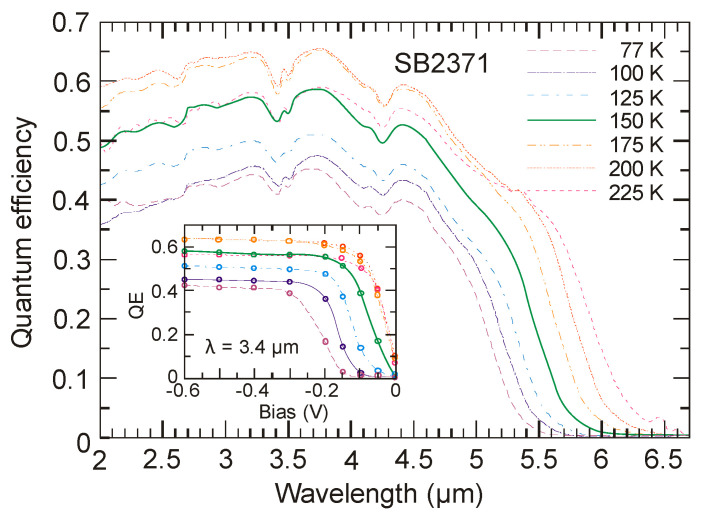
BSI spectral *QE* for MWIR detector for temperatures ranging from 77 to 225 K. The inset shows the *QE* measured at 3.4 µm versus voltage at the same set of temperatures [171].

The *QE* shown in Figure 63 is derived from measured responsivity of the BSI samples through the GaSb substrate. The *QE* increases within the range 77–175 K (probably due to reduced hole mobility), and next decreases in the higher temperature (probably due to influence of interfacial detects at the barrier/absorber interface). The insert in the figure for *λ_cut-off_* = 3.4 μm shows that a low reverse voltage is sufficient to reach maximum responsivity, where hB AlAsSb doping influence on the value of nonzero turn-on voltage is visible.

**Figure 64:**
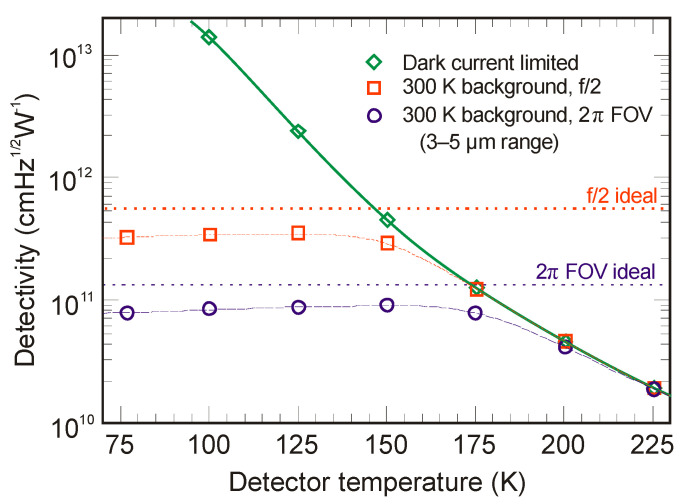
300 K background, 3–5 µm blackbody detectivity versus temperature for *f*/2 optics and 2π FOV (dashed lines); the corresponding ideal D* values are indicated by dotted lines. The shot noise limited D* is also depicted (solid line) (after [171]).

The MWIR T2SLs InAs/InAsSb nBn detector operating in HOT conditions has demonstrated significant advantages over InSb. It is presented in Figure 64, where the 300 K background limited detectivity versus detector temperature for *f*/2 optics and 2π field of view (FOV) is shown. The assessed BLIP temperatures are 152 K and 181 K for *f*/2 and 2π FOV, respectively. In this way, this barrier detector combines the higher operating temperature (comparable with HgCdTe) with the material robustness of InSb. However, for low-background applications, the T2SLs InAs/InAsSb barrier detector still has disadvantages compared to HgCdTe-higher G-R dark current due to shorter SRH lifetimes [171].

Hood et al. [192] modified the barrier structure to present better in performance pBn T2SLs InAs/GaSb LWIR device. In the proposed architecture, the p-n junction can be located at the interface between the heavily doped p-type contact and the low doped barrier or within the low doped barrier itself. Similarly to the nBn, the pBn architecture reduces G-R currents related to the SRH centers (the depletion region is moved to the barrier-no depletion in the low bandgap n-type absorber). In addition, the electric field dropping in the barrier increases the response time which is related to the carriers sweeping from the active layer. Recently, the design, growth, and characterization of the first MWIR T2SLs InAs/InAsSb pBn barrier photodetectors for HOT condition have been demonstrated [176,193].

In the last several years the idea of barrier detectors has been extended to short wavelength infrared wavelength (SWIR) region using InGaAs and InGaAsSb alloy systems [107,194,195,196]. The MBE technology is used as a standard growth method for SWIR detectors fabrication.

Savich et al. [195] carried out a comparison of electrical and optical performance of the typical photodiodes and nBn detectors with *λ_cut-off_* = 2.8 μm fabricated with both lattice-mismatched InGaAs and lattice-matched InGaAsSb active layers. In order to minimize the number of defects in the InGaAs active layer grown on InP substrate, a 2 μm AlInAs step-graded buffer was grown where the lattice constant was graded from that of InP to that of In_0.82_Ga_0.18_As. Both the typical photodiode and nBn detector include this step-graded buffer. The extra pseudomorphic AlAsSb unipolar barrier is added to maintain a high barrier in conduction band in comparison to In_0.82_Ga_0.18_As.

In the case of lattice-matched to GaSb, the quaternary composition of In_0.30_Ga_0.70_As_0.56_Sb_0.44_ at the edge of the miscibility gap was used to maintain the *λ_cut-off_* and lattice-matching requirements. In nBn detector, also pseudomorphic AlGaSb unipolar barrier with a large CBO and zero VBO in comparison to the In_0.30_Ga_0.70_As_0.56_Sb_0.44_ absorber was implemented. 

**Figure 65:**
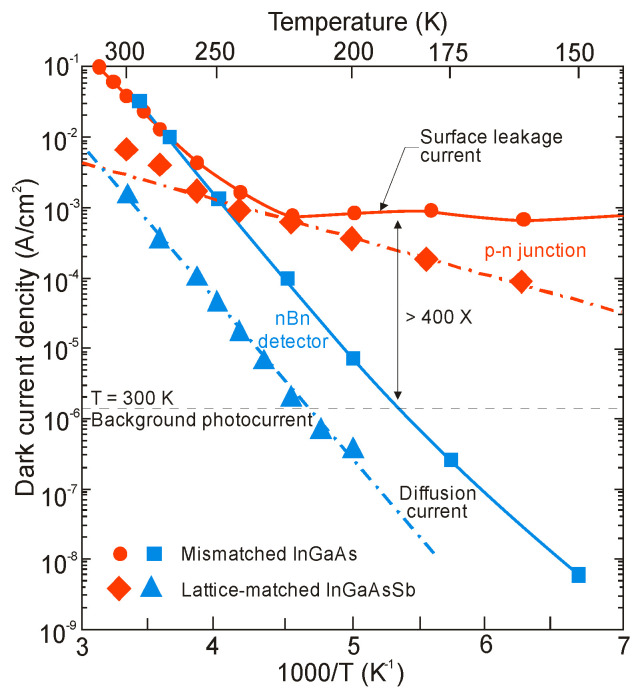
Arrhenius plots for both InGaAs and InGaAsSb p-n junctions and nBn detectors with *λ_cut-off_* =2.8 µm (adapted after [195]).

Figure 65 shows temperature dependent dark current characteristics for both lattice-mismatched InGaAs and lattice-matched InGaAsSb detectors under reverse bias 100 mV [195]. InGaAs on InP materials exhibit low material quality, i.e., threading dislocations occurring due to the lattice mismatch increasing dark current.

The p-n InGaAs photodiode is limited by surface leakage current for *T* < 220 K, while the nBn detector remains diffusion limited down to 150 K. At the room temperature background photocurrent level, the nBn detector’s dark current is reduced by 400× in comparison to the typical photodiode.

The p-n InGaAsSb device is limited by depletion region current for *T* < 250 K, while the nBn remains diffusion limited down to 250 K. At the 300 K background photocurrent level of the nBn, dark current is suppressed by nearly three orders of magnitude in comparison to the typical photodiode.

**Figure 66:**
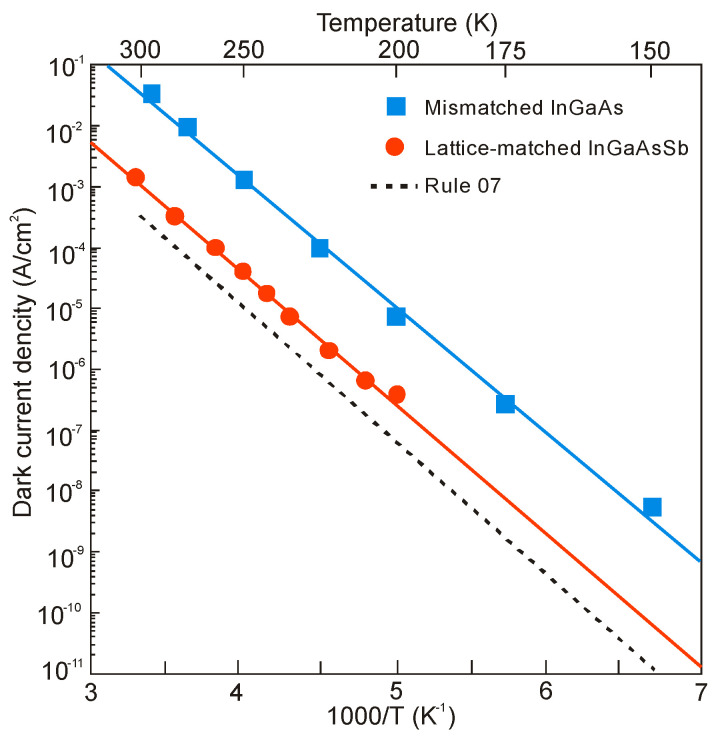
InGaAs and InGaAsSb nBn dark current comparison. The lattice matched InGaAsSb barrier detector exhibits at least an order of magnitude reduction in the dark current in comparison to the mismatched InGaAs nBn detector (after [195]).

Figure 66 shows that InGaAsSb nBn detector being lattice-matched to a GaSb substrate presents performance near “Rule 07” where dark current is 10 to 20 times lower in comparison to the lattice-mismatched InGaAs counterpart.

**Figure 67:**
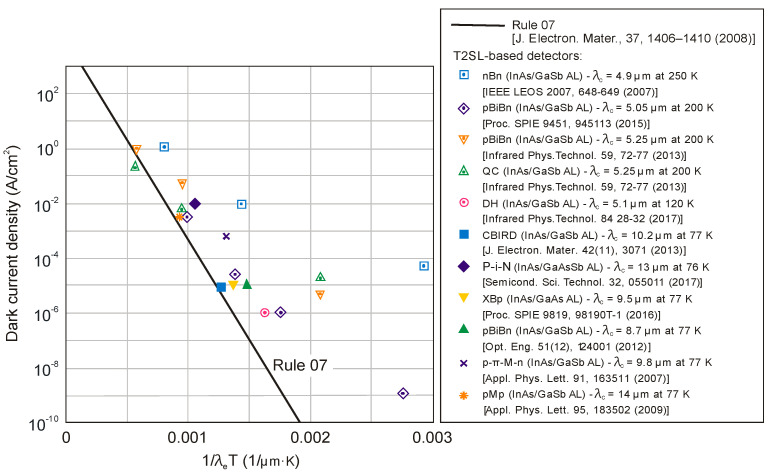
Comparison of various types InAs/GaSb SL detectors with the “Rule 07”.

**Figure 68:**
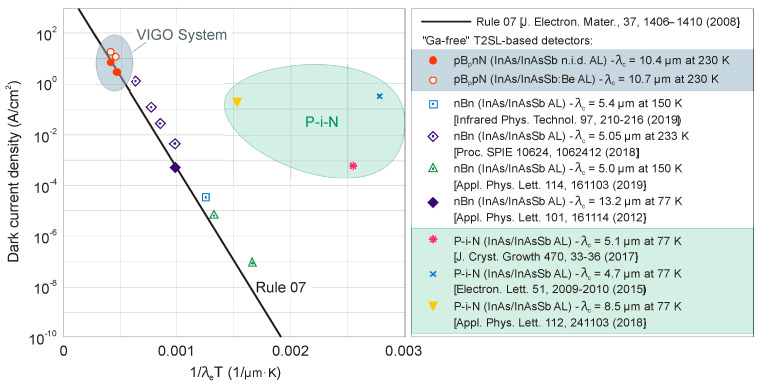
Comparison of various types Ga-free InAs/InAsSb SL detectors with the “Rule 07”.

Figure 67 and Figure 68 compare dark currents of different types of barrier and p-i-n detectors constructed on the basis of InAs/GaSb and Ga-free InAs/InAsSb SLs, respectively. Experimental data collected from the literature [107,143,147,155,161,163,173,181,196,197,198,199,200,201,202] is referred to the “Rule 07”. The dark currents of the InAs/GaSb SL detectors differ more from “Rule 07” than the data for Ga-free SL detectors. The best results were achieved by the pMp and the CBIRD detectors, both operating at 77 K. Fabricated by VIGO System Ga-free InAs/InAsSb SLs pBnN detector reaches dark currents measured at high temperatures (210 K and 230 K) in accordance with the “Rule 07” -see Figure 68. Similar results were reached by the detector described in the work of Kim et al. [163] but operating in 77 K. This figure also shows the advantage of barrier detectors over P-i-N devices.

#### 6.2.3. Multicolor Detectors

**Figure 69:**
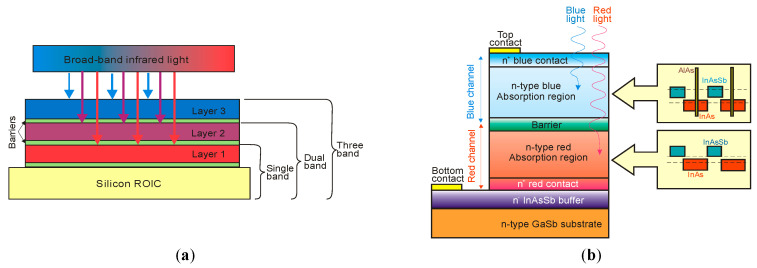
Multicolor detector cell: (**a**) architecture of a three-band photodetector. The very first IR band is absorbed in layer 3, while longer wavelength is transmitted through to the next constituent layers. The active layers are separated by thin barriers. (**b**) SWIR/MWIR dual-band nBn T2SLs InAs/InAsSb back-to-back p-i-n-n-i-p structure and schematic band alignment of SLs building active layers (dotted lines represent the effective bandgaps of SLs) (after [168]).

Apart from HgCdTe photodiodes and quantum well infrared photodetectors (QWIPs), T2SLs have emerged as a candidate suitable for multispectral detection due to flexibility in bandgap tuning assuming lattice-matching requirements [203]. The single multicolor detector cell is built of the group of detectors being sensitive to a given spectral band (see Figure 69). The radiation goes through the SWIR detector, while the MWIR/LWIR passes freely to the next detector. The constituent layers absorb IR up to its *λ_cut-off_* being transparent to the longer wavelengths collected in subsequent layers. The detector structure is fabricated by proper order: MWIR/LWIR optically behind SWIR detector. The operating IR range is addressed by changing the polarity of the voltage.

Northwestern University presented different types of bias-switchable dual-band nBn T2SLs InAs/InAsSb detectors to include MWIR/LWIR and LWIR1/LWIR2 ranges [166,168,204]. The fabrication of dual-band photodetectors is challenging to grow proper electron barrier and SLs designs for both absorption regions with zero VB discontinuity.

Figure 69b presents schematic diagram of a bias-selectable nBn dual-band IR detector based on T2SL InAs/InAsSb/AlAsSb structure together with band alignment of the SLs in two absorption layers. The SLs design of the red absorption region, with nominal *λ_cut-off_* ~12 μm at 77 K, consists of 30/10 MLs of T2SLs InAs/InAs_0.48_Sb_0.52_, respectively. The SLs design of the blue absorption region, with a nominal *λ_cut-off_* ~8 μm at 77 K, consists of 13/1.5/13/9 MLs of T2SLs InAs/AlAs/InAs/InAs_0.48_Sb_0.52_, respectively. The SLs blue channel absorption region contains extra AlAs layer providing the necessary flexibility in the band structure design. The AlAs layer compensates the excessive tensile strain generated by the InAsSb layer in the SLs. The electron barrier is made of 4/3/4/3/4/9 MLs of T2SLs InAs/AlAs_0.55_Sb_0.45_/InAs/AlAs_0.45_Sb_0.55_/InAs/InAs_0.45_Sb_0.55_ with a nominal *λ_cut-off_* ~4 μm at 77 K. This barrier design provides more flexibility to accommodate the lattice strain.

**Figure 70:**
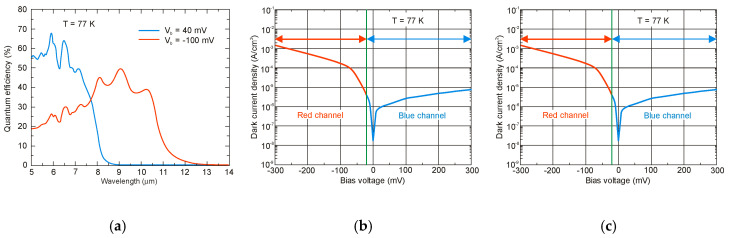
Performance characteristics of bias-selectable dual-band nBn InAs/InAsSb SL detector at 77 K: (**a**) spectral *QE* in FSI configuration without any anti-reflection coating for two applied bias: + 40 mV (blue channel) and −100 mV (red channel), (**b**) dark current density versus voltage for blue and red channels, (**c**) D* of the blue and red channels at +40 and −100 mV voltage (after [168]).

The two active regions are selected by changing the voltage polarity. The optical performance of the detector is shown in Figure 70a. The blue and red channels exhibit 100% *λ_cut-off_* ~8.6 μm (~145 meV) and ~12.5 μm (~100 meV) at 77 K, respectively. The blue channel *QE* saturates at the level of 65% at 6.45 μm and the red channel reaches 50% at 9 μm.

Figure 70b shows the dark current density of the dual-band LWIR1/LWIR2 photodetector measured when covered by a 77K cold-shield. At +40 mV, dark current density for the blue channel reaches 1.13 × 10^−^^6^ A/cm^2^ and detectivity reaches 5.1 × 10^12^ cmHz^1/2^/W at 77 K. The red channel exhibits dark current density at the level of 1.7 × 10^−^^4^ A/cm^2^ at −100 mV and detectivity of 4.5 × 10^11^ cmHz^1/2^/W. The detectivity is estimated using Equation (15).

The results presented here indicate that the T2SLs InAs/InAsSb have the potential for multispectral IR imagers and can be a possible alternative for current state-of-the-art T2SLs InAs/GaSb.

## 7. InAsSb-based Superlattice Focal Plane Arrays

The noise equivalent difference temperature (*NEDT*) is a figure of merit for thermal imagers giving information on the detector array sensitivity and can be expressed according to the relations [3,78].

(16) $$NEDT=\frac{1+\left(J_{dark}/J_{\Phi }\right)}{\sqrt{N_{w}}\left(\frac{1}{\Phi_{B}}\;\frac{d\Phi_{B}}{dT}\right)},$$

(17) $$J_{\Phi }=q\eta \Phi_{B},$$

(18) $$\sqrt{N_{w}}=\frac{\left(J_{dark}+J_{\Phi }\right)\tau_{int}}{q},$$

where *Φ_B_*—background flux, *τ_int_*—integration time, *N_w_*-well capacity of readout, *J_Φ_* = *ηΦ_B_A*—background flux current. The overall *QE* of the detector, including the internal *QE* is given by *η*. The cold shield efficiency and optics transmission are taken to be unity.

Assuming that the scene contrast, C = *(dΦ_B_/dT)/Φ_B_*, *NEDT* can be rewritten:

(19) $$NEDT=\frac{1+\left(J_{dark}/J_{\Phi }\right)}{\sqrt{N_{w}}C}.$$

The calculated *C* for MWIR and LWIR is 3.5–4% and 1.7% at 300 K, respectively. Well capacity for a 15-μm pixel stays within the range *N_w_* = 1 × 10^6^ to 1 × 10^7^ electrons. Equation (18) indicates that if the *I_dark_*/*I_Φ_* ratio increases and/or *QE* decreases, the longer integration time and the faster optics have to be implemented. 

**Figure 71:**
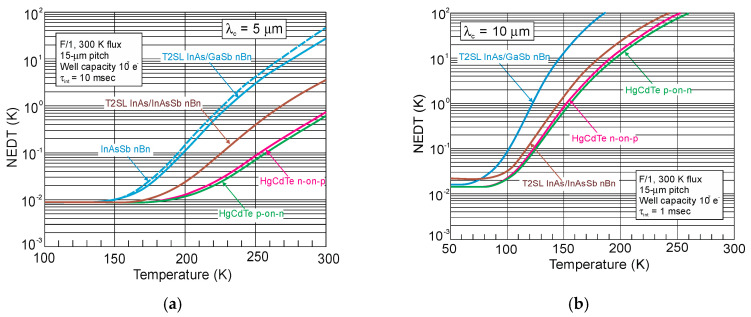
*NEDT* versus temperature for barrier detectors and HgCdTe photodiodes for *λ_cut-off_* = 5 µm (**a**) and 10 µm (**b**).

Figure 71 presents *NEDT* versus temperature for barrier detectors and HgCdTe photodiodes with *λ_cut-off_* = 5 μm and 10 μm. The theoretical HgCdTe photodiodes performance limit is higher than for barrier detectors for *T* > 150 K in a MWIR and *T* > 80 K in a LWIR range. However, taking into account both types of T2SLs, the operating temperature of InAs/InAsSb based detector is higher. For low temperatures the figure of merit of both T2SLs allows for similar performance to be reached, being mainly limited by the readout circuits.

### 7.1. InAsSb nBn Detector FPAs

The MWIR InAsSb nBn arrays are fabricated by several companies. The nBn cell is considered to be self-passivating design, decreasing leakage current and improving reliability and manufacturability. The simple design (see Table 5) is a major advantage in the large IR FPA state of the art.

First nBn InAsSb array commercially developed by Lockheed Martin Santa Barbara Focalplane operates within the temperature range *T* = 145–175 K. The 12 μm pixel-pitch MWIR nBn sensor of the 1280 × 1024 format is packaged in a 1.4” diameter dewar with net housing length ~3.8” to include the cooler. That cooler and electronics are reported to consume 5 W and guarantees 25k hours of operation [205].

**Figure 72:**
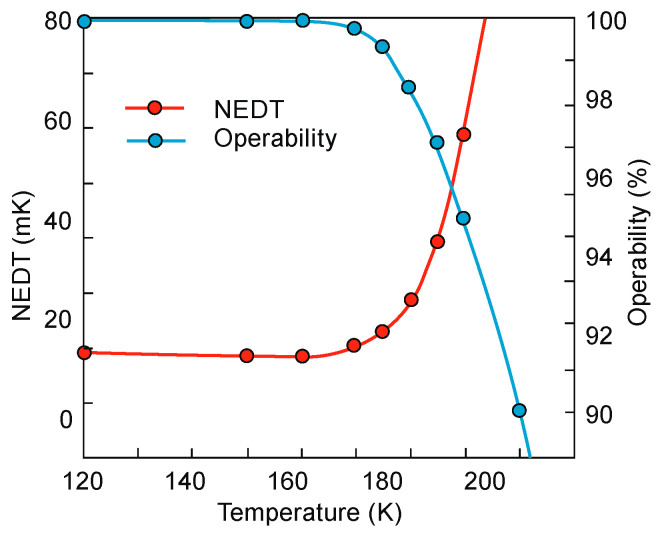
“Kinglet” *NEDT* and pixel operability temperature dependence for *f*/3.2 optics (after [206]).

Klipstein et al. demonstrated nBn array operating in the MWIR (3.4–4.2 μm) at *T* = 150 K, with *f*/5.5 optics developed by SCD known as “Kinglet” [206]. The 640 × 512 “Kinglet” is based on SCD’s Pelican-D readout integrated circuit (ROIC) and 15-μm pitch InAs_0.91_Sb_0.09_/B-AlAsSb architecture. The *NEDT* for *f*/3.2 optics and the pixel operability versus temperature is presented in Figure 72, reaching 20 mK at *τ_int_* = 10 ms and the pixel operability after a typical two point nonuniformity correction is higher than 99.5%, respectively. The *NEDT* and operability change sharply for *T* > 170 K, being consistent with the BLIP temperature of 175 K.

**Figure 73:**
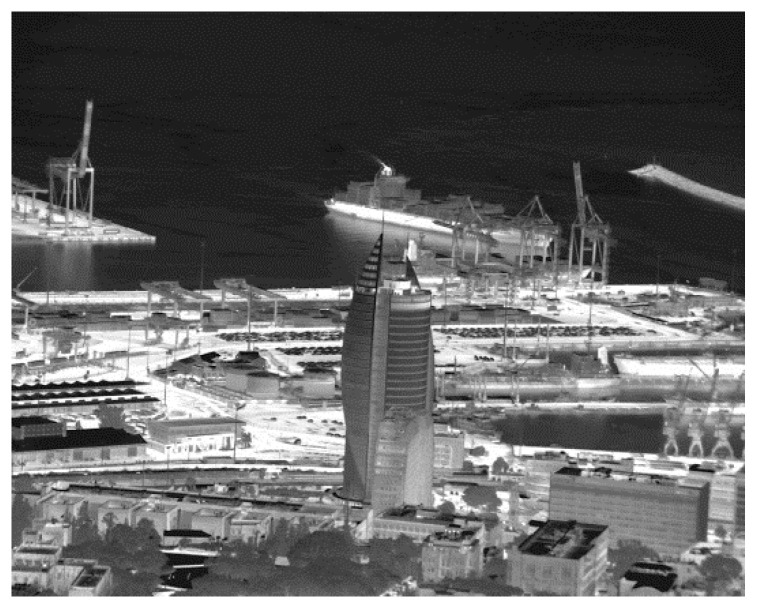
Image taken by 15 µm HOT Hercules 1280 × 1024, InAs_0.91_Sb_0.09_ XBn FPA at *f*/3 optics, and 150 K (after [208]).

Recently, SCD has presented several 10 μm pitch nBn 1920 × 1536, 1280 × 1024 and 640 × 512 arrays, integrated with the Blackbird ROICs designed for HOT conditions [207]. Table 6 summarizes the performance of two InAsSb barrier devices fabricated by IR Cameras and SCD. In comparison with standard InSb photodiode arrays, the integrated detector cooler assembles (IDCAs) of nBn devices are characterized by SWaP advantages. For 15-µm pitch 1280 × 1024 FPA, the IDCA weight is reduced by 10%, the power consumption by 70%, and the mean time to failure (MTTF) is increased by more than 100%. Figure 73 presents an image reached by a HOT Hercules array at 150 K [208]. The very first demonstration of the 5 µm pixels 2040 × 1156 was reported in [209].

### 7.2. InAs/InAsSb Superlattice Detector FPAs

T2SLs are especially helpful for fabrication of barrier detectors due to almost perfect control of band edge alignments. Their compatibility with conventional p-n photodiode architecture gives further enhancement in performance improvement. As was first shown by Savich at el. [210], depending on the barrier placement, different dark current components are filtered.

**Figure 74:**
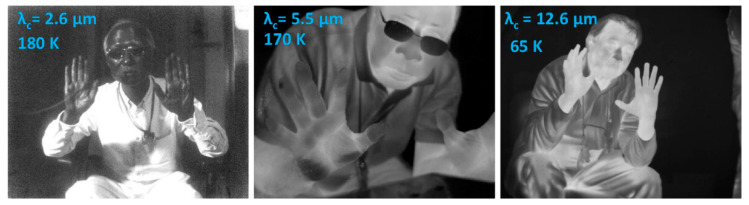
e-SWIR, MIWR, and LWIR images registered by Sb-based FPA. The e-SWIR FPA is based on a bulk InGaAsSb, T2SLs InAs/InAsSb active layer was used for the MWIR and LWIR (after [107]).

JPL demonstrated extended SWIR (e-SWIR), MWIR, and LWIR images taken with Sb-based FPAs. Whereas the e- SWIR detector is based on a bulk InGaAsSb absorber, the T2SLs InAs/InAsSb active layers were used for MWIR and LWIR. Table 7 presents high-performance, cost-effective 640 × 512 FPAs for MWIR and LWIR ranges. HOT nBn photodetectors operate at 150 K covering the near infrared (NIR, 0.75–1.4 µm), SWIR (1.4–3 µm), and MWIR atmospheric transmission window (3–5 µm) with very high *QE*. Figure 74 shows 640 × 512 FPAs images taken in different spectral ranges.

**Figure 75:**
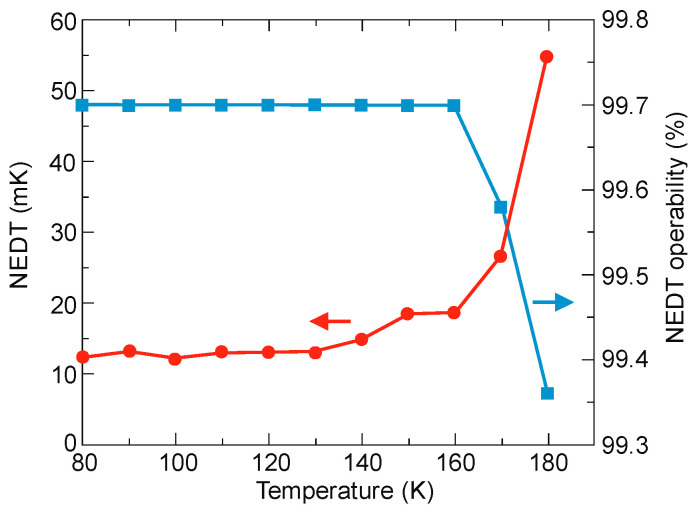
*NEDT* and operability versus temperature for 24 µm pitch MWIR 640 × 512 nBn InAs/InAsSb FPA.

Figure 75 shows the performance of the 24 μm pitch 640 × 512 FPA with *λ_cut-off_* ~5.4 μm (see Table 7) and direct injection (DI) ROIC with *N_w_* = 8 × 10^6^ electrons. *NEDT* is below 19 mK for *T* < 160 K, and increases up to ~55 mK at 180 K. The operability is 99.7% up to 160 K and decreases to 99.3% at 180 K. *NEDT* exhibits excellent uniformity with 8 mK distribution [172]. Both *NEDT* and operability are similar to those described previously for 15 μm pitch nBn InAs_0.91_Sb_0.09_/B-AlAsSb 640 × 512 FPA shown in Figure 72.

**Figure 76:**
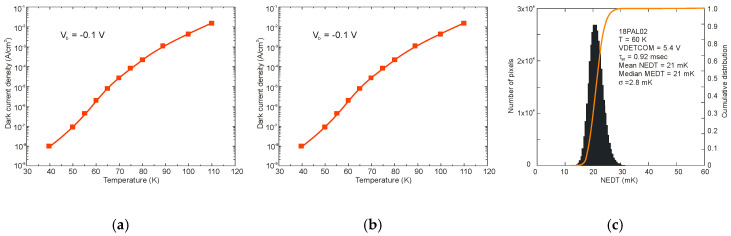
The LWIR *(λ_cut-off_* = 11 µm at 60 K) nBn T2SLs InAs/InAsSb photodetectors performance: (**a**) dark current density versus temperature for −0.1 V, (**b**) BSI *QE* (no AR coating) measured for *T* = 60–75 K, (**c**) *NEDT* for 640 × 512 FPA for *T* = 60 K, 300 K blackbody temperature and *f*/4 cold stop (after [212]).

The LWIR barrier structures have also shown significant improvement in operating temperature in comparison to the typical p-n designs. Figure 76 presents the dark current density and the *QE* versus temperature for LWIR square mesa T2SLs InAs/InAsSb barrier detectors (area 250 × 250 µm^2^) [212]. The devices were grown on a 4”, Te-doped GaSb (100) substrate by MBE. The −50 mV dark current density at 70 K is 4 × 10^−5^ A/cm^2^. The *QE* derived from BSI (through the GaSb substrate) responsivity measurements has not been corrected for substrate reflection or transmission.

The LWIR detector was used to fabricate 24 µm pitch, 640 × 512 FPA and Lockheed Martin SBF-193 ROIC. The measured 640 × 512 FPA *NEDT* histogram for *T* = 60 K, 300 K blackbody temperature and *f*/4 cold stop is shown in Figure 76c. The 21 mK *NEDT* is in fair agreement with the estimated *NEDT* based on a single element test detector data [212].

## 8. Conclusions

There are many critical challenges for future civilian and military IR detector applications. The main efforts are directed to decrease IR imaging systems SWaP by increasing the FPA’s operating temperature. Nowadays, HgCdTe is the most common material for IR photodetectors being the reference for alternative technologies to include uncooled operation. After sixty years of development, HgCdTe has just reached ultimate HOT performance limit estimated by “Law 19”-the ultimate photodiode performance metric [117]. This metric has been recently supported by experimental data of fully-depleted HgCdTe P-i-N photodiodes. The “Law 19” corresponds exactly the BLIP for room temperature operation.

The InAsSb-based IR photodetectors’ performance is comparable with HgCdTe:

- III-V compounds exhibit inherently short SRH lifetimes typically below 1 μs requiring the nBn design to operate at higher temperatures (this applies both to the bulk InAsSb and the T2SLs),
- T2SLs InAs/InAsSb has emerged in recent years as an alternative to the T2SLs InAs/GaSb due to the three reasons: (i) a better growth controllability and simpler manufacturability, (ii) longer SRH lifetimes (10 μs in MWIR SLs in comparison with 400 ns for InSb), and (iii) significantly higher operating temperature than InSb,
- HgCdTe exhibits long SRH lifetimes >200 μs to 50 ms depending on the *λ_cut-off_* [78] (can operate with either architecture and may be diffusion or depletion current limited),
- III-Vs exhibit comparable performance to HgCdTe for equivalent *λ_cut-off_*, but for lower temperature, due to the inherent difference in SRH lifetimes,
- III-V materials are more robust than their II-VI counterparts due to stronger (less ionic) chemical bonding what allows to reach better operability, spatial uniformity, temporal stability, scalability, producibility, and affordability-the so-called “ibility” advantages.

Table 8 gathers the LWIR detectors state of the art fabricated from selected materials. The highest level of maturity (technology readiness level, TRL = 9) is credited to HgCdTe photodiodes, microbolometers, and for QWIPs. The T2SLs InAs/InAsSb structures have proven the potential for MWIR and LWIR applications with performance comparable to HgCdTe for the same *λ_cut-off_*. Strong progress toward mature SLs and barrier detector technologies, including their commercialization, has been observed in the last decade. It is expected that InAsSb-based materials strengthen dominant position of InSb especially in MWIR FPA market in volume due to “ibility” advantages of III-V semiconductors. HgCdTe detectors allow one to reach lower dark current and HOT conditions and are/will be a material of choice for more demanding applications.

## Figures and Tables

**Table 1:** MOCVD and MBE methods comparison (after [28]).

Category	MBE	MOCVD
**Growth**	Fast switching for proper interfaces.	High growth rate for bulk layers.
	Able to grow thermodynamically forbidden materials.	Growth near thermodynamic equilibrium, excellent quality/crystallinity.
	No hydrogen passivation, no burn in inherent to MOCVD.	Ability to control background doping.
	Uniformity easier to tune, largely set by reactor geometry.	
**Economic**	Longer individual campaigns, less setup variability.	Shorter maintenance periods.
		More flexibility for source and reactor configuration changes.
	Overhead does not scale with run rate. Contribution per wafer increases with wafer volume.	Economic to idle. Overhead cost scales with run rate.
	Lower material cost/wafer.	Higher safety risk, increasing scrutiny of legislative bodies worldwide.

**Table 2:** Fundamental properties of semiconductors used in IR photodetectors manufacturing.

Semiconductor	Si	Ge	GaAs	AlAs	InP	InGaAs	AlInAs	InAs	GaSb	AlSb	InSb	HgTe	CdTe
Family	IV	IV	III–V	III–V	III–V	III–V	III–V	III–V	III–V	III–V	III–V	II–VI	II–VI
Lattice constant (Å)/structure	5.431 (D)	5.658 (D)	5.653 (ZB)	5.661 (ZB)	5.870 (ZB)	5.870 (ZB)	5.870 (ZB)	6.058 (ZB)	6.096 (ZB)	6.136 (ZB)	6.479 (ZB)	6.453 (ZB)	6.476 (ZB)
Bulk modulus (Gpa)	98	75	75	74	71	69	66	58	56	55	47	43	42
Band gap (eV)	1.124 (id)	0.660 (id)	1.426 (d)	2.153 (id)	1.350 (d)	0.735 (d)		0.354 (d)	0.730 (d)	1.615 (id)	0.175 (d)	−0.141 (d)	1.475 (d)
Electron effective mass	0.26	0.39	0.067	0.29	0.077	0.041		0.024	0.042	0.14	0.014	0.028	0.090
Hole effective mass	0.19	0.12	0.082 (L) 0.45 (H)	0.11 (L) 0.40 (H)	0.12 (L) 0.55 (H)	0.05 (L) 0.60 (H)		0.025 (L) 0.37 (H)	0.4	0.98	0.018 (L) 0.4 (H)	0.40	0.66
Electron mobility (cm^2^/Vs)	1450	3900	8500	294	5400	13800		3 × 10^4^	5000	200	8 × 10^4^	26500	1050
Hole mobility (cm^2^/Vs)	505	1900	400	105	180			500	880	420	800	320	104
Electron saturation velocity (10^7^ cm/s)	1.0	0.70	1.0	0.85	1.0			4.0			4.0		
Thermal cond. (W/cmK)	1.31	0.31	0.5		0.7			0.27	0.4	0.7	0.15		0.06
Relative dielectric constant	11.9	16.0	12.8	10.0	12.5			15.1	15.7	12.0	17.9	21	10.2
Substrate	Si, Ge		GaAs		InP			InAs, GaSb			InSb	CdZnTe, GaAs, Si	
MW/LW detection mechanism	Heterojunction internal photoemission		QWIP, QDIP		QWIP			Bulk (MW) Superllatice (MW/LW) Band-to-band (B-B)			Bulk BtB	Bulk BtB	

D—diamond, ZB—zincblende, id—indirect, d—direct, L—light hole, H—heavy hole, QWIP—quantum well infrared photodetectors, QDIP—quantum dot infrared photodetectors.

**Table 3:** Physical properties of narrow gap III–V alloys.

	T(K)	InAs	InSb	GaSb	InAs_0.35_Sb_0.65_
Lattice structure		cub.(ZnS)	cub.(ZnS)	cub.(ZnS)	cub.(ZnS)
Lattice constant a (nm)	300	0.60584	0.647877	0.6094	0.636
Thermal expansion coefficient α (10^−6^ K^−1^)	300 80	5.02	5.04 6.50	6.02	
Density ρ (g/cm^3^)	300	5.68	5.7751	5.61	
Melting point T_m_ (K)		1210	803	985	
Energy gap E_g_ (eV)	4.2	0.42	0.2357	0.822	0.138
	80	0.414	0.228		0.136
	300	0.359	0.180	0.725	0.100
Thermal coefficient of E_g_	100–300	–2.8 × 10^−4^	–2.8 × 10^−4^		
Effective masses:					
$m_{e}^{*}/m$	4.2	0.023	0.0145	0.042	
	300	0.022	0.0116		0.0101
$m_{\mathrm{lh}}^{*}/m$	4.2	0.026	0.0149		
$m_{\mathrm{hh}}^{*}/m$	4.2	0.43	0.41	0.28	0.41
Momentum matrix element P (eVcm)		9.2 × 10^−8^	9.4 × 10^−8^		
Mobilities:	77	8 × 10^4^	10^6^		
μ_e_ (cm^2^/Vs)	300	3 × 10^4^	8 × 10^4^	5 × 10^3^	5 × 10^5^
	77		1 × 10^4^	2.4 × 10^3^	5 × 10^4^
μ_h_ (cm^2^/Vs)	300	500	800	880	
Intrinsic carrier concentration *n_i_* (cm^−3^)	77	6.5 × 10^3^	2.6 × 10^9^		2.0 × 10^12^
	200	7.8 × 10^12^	9.1 × 10^14^		8.6 × 10^15^
	300	9.3 × 10^14^	1.9 × 10^16^		4.1 × 10^16^
Refractive index *n_r_*		3.44	3.96	3.8	
Static dielectric constant *ε_s_*		14.5	17.9	15.7	
High frequency dielectric constant *ε_∞_*		11.6	16.8	14.4	
Optical phonons:					
LO (cm^−1^)		242	193		≈220
TO (cm^−1^)		220	185		≈200

**Table 4:** InAs/InAsSb superlattice infrared detectors.

	SL Period [nm]	Polarity	Size [µm]	Absorber Thickness [µm]	T [K]	*λ_c_* [µm]	*J_d_ (T,V_b_)* [A/cm^2^]	R_v_ (λ,T,V_b_);QE [A/W]	D*( λ, T, V_b_) [cmHz^1/2^/W]	References
InAs/InAs_0.62_Sb_0.38_	18.4	nBn	s = 410	2.2	77	13.2	5 × 10^−4^ (77, −0.3 V)	0.24 (12, 77, −0.3)	1 × 10^8^ (12, 77, −0.3)	Kim et al. [163]
InAs/InAs_0.57_Sb_0.43_	14.7		d = 100–400	2.3	77	14.6	0.7 (77, −0.3 V)	4.8 (12.8, 77, −0.3); ~46%	4.6 × 10^11^ (4.5, 150, −0.2)	Hoang et al. [164]
InAs/InAs_0.45_Sb_0.55_	10.5	pN	s = 100–400	2.6	77	10	4.4 × 10^−4^ (77, −0.09 V)	3.5 (7.9, 77, −0.09); ~54%	2.8 × 10^11^ (7.9, 77, −0.09)	Haddadi et al. [165]
Dual band InAs/AlAs/InAs/ InAs_0.48_Sb_0.52_ InAs/InAs_0.48_Sb_0.52_	6.3 10.5	nBn	s = 100–400	2	77	5.1 9.5	1 × 10^−7^ (77, 0.1 V) 5.7 × 10^−4^ (77, −0.15 V)	1.5 (4, 77, 0.1); ~45% 2.25 (7, 77, −0.15); ~40%	8.2 × 10^12^ (4, 77, 0.1) 1.6 × 10^11^ (7, 77, −0.15)	Haddadi et al. [166]
InAs/InAs_0.50_Sb_0.50_	12	nBn	s = 100–400	2	77	10	8 × 10^−5^ (77, −0.08 V)	2.65 (7.5, 77, −0.8); ~43%	4.7 × 10^11^ (7.5, 77, −0.08)	Haddadi et al. [167]
Dual band InAs/AlAs/InAs/ InAs_0.48_Sb_0.52_ InAs/InAs_0.48_Sb_0.52_	22 25	nBn	s = 100–400	4	77	8.7 12.5	1.13 × 10^−6^ (77, 0.04 V) 1.7 × 10^−4^ (77, −0.1 V)	3.37A/W (6.45, 77, 0.04); 65% 3.7 A/W (9, 77, −0.1); 50%	5.1 × 10^12^ (6.45, 77, 0.04) 4.5 × 10^11^ (9, 77, −0.1)	Haddadi et al. [168]
InAs/InAsSb T2SLs			d = 0.25, 1		208	5.0	1 × 10^−4^ (208, −0.3 V)	1.35 (4.1, 208, −0.3 V)	2.6 × 10^10^ (4.1, 208, −0.3 V)	Teledyne Judson [139]
InAs/InAs_0.65_Sb_0.35_	5.5	nBn	d = 60–300	3	150	4.6	4 × 10^−5^ (150, −2.5 V)			Perez et al. [170]
InAs/InAs_0.66_Sb_0.34_	5	nBn	s = 250	2.6	150	5.4	4.5 × 10^−4^ (150, −0.2 V)	~52%	4.6 × 10^11^ (4.5, 150, −0.2 V)	Ting et al. [171,172]
InAs/InAs_0.65_Sb_0.35_	12	pin	s = 400	2	77	8.0	9 × 10^−4^ (77, −20 mV)	1.26 (7.5, 77, −); ~21%	5.4 × 10^10^ (7.5, 77, −)	Wu et al. [173]
InAs/InAs_0.79_Sb_0.21_	6.6	pin		0.2–1.9	150	5.0	3.3 × 10^−4^ (150, −20 mV)	1.76 (4, 150, 0); ~55%	1.2 × 10^11^ (4, 150, −0.02)	Wu et al. [174]
InAs/InAs_0.65_Sb_0.35_	5.15	nBn	d = 60–310	3	150	5	1 × 10^−3^ (150, −0.5 V)			Durlin et al. [175]
InAs/InAs_0.5_Sb_0.5_	7.4	pBn	d = 100–400	2.1	150	4.4	1.16 × 10^−5^ (150, −0.05 V)	1.48 (4.4, 150, −0.05); 47%	7.1 × 10^11^ (4.4, 150, −0.05)	Wu et al. [176]
InAs/InAs_0.84_Sb_0.16_ monolithically Si-integrated	13.3	nBn	d = 50	4	200	5.5	1.4 × 10^−2^ (200, −0.1)	0.88 (5, 200, −0.1); 25%	1.5 × 10^10^ (5.0, 200, −0.1)	Delli et al. [177]
InAs/InAs_0.8_Sb_0.2_	13.5	p-n	d = 320	1	150	4.5	9.1 × 10^−6^	0.78 (4.5, 150); 25%	3.4 × 10^11^ (4.5, 150)	Wu et al. [178]
InAs/AlAsSb/InAs/InAsSb/ AlAsSb/InAsSb	-	p-i-n	s = 100–400	1	300	1.8	9.6 × 10^−5^ (300, −0.05)	0.47 (1.8, 300); 37%	6.45 × 10^10^ (1.8, 300, −0.05)	Haddadi et al. [179]

d-diameter of detector; s-square detector.

**Table 5:** T2SLs barrier detectors.

Flat-Band Energy Diagrams	Example of Detector Structure	Description	Reference
**nBn** 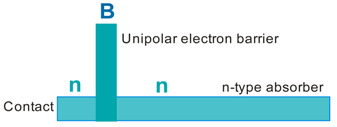	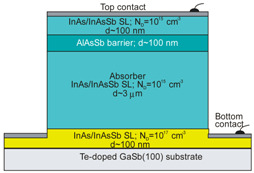	The top contact, absorber, and bottom contact layers are built of of T2SLs InAs/InAsSb. As a unipolar barrier located between the top contact and absorber, about 100-nm thick AlAsSb Be-doped to 10^15^ cm^−3^ is used. The top contact and absorber layers are n.i.d while the bottom contact is Te-doped to 10^17^ cm^−3^.	[171]
**pBn barrier** 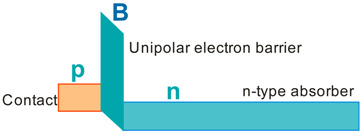	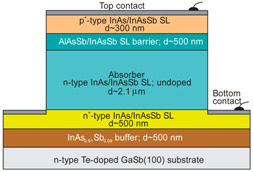	The epitaxial growth starts with a 700 nm thick n^+^-doped GaSb/InAs_0.91_Sb_0.09_ buffer layer, next a 500 nm thick n^+^-doped bottom contact layer, a 2.1 μm n.i.d active layer, a 500 nm electron barrier, and a 300 nm thick p-type top contact. The top/bottom contacts and active layer are built of T2SLs InAs/InAsSb. Si and Be are used for n-type and p-type dopants, respectively.	[176]
**pBp** 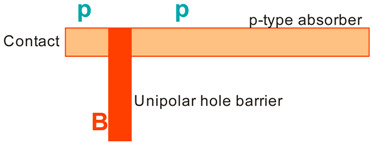	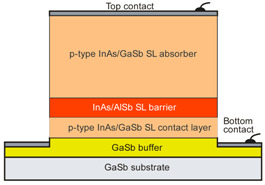	The contact layers and absorber are built of T2SLs InAs/GaSb. The barrier layer is based on a T2SLs InAs/AlSb. The proper lattice match to the GaSb substrate is ensured by InSb-like interfaces.	[189]
**CBIRD (Complementary barrier infrared detector)** 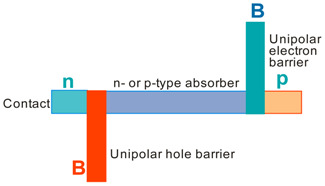	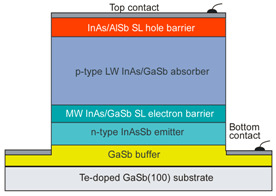	The detector is built of a lightly p-type T2SLs InAs/GaSb active layer being sandwiched between n-type T2SLs InAs/AlSb hole barrier (hB) and wider T2SLs InAs/GaSb electron barrier (eB). The barriers are designed to exhibit zero CBO and VBO in relation to the active layer. A heavily doped n-type InAsSb layer adjacent to the eB plays the role of the bottom contact. The SRH and trap-assisted tunneling (TAT) are reduced by the N-p heterojunction between the hB and active layer.	[190]

**Table 6:** nBn InAsSb FPA characteristics.

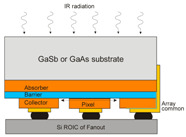 **nBn Detector Array Architecture**	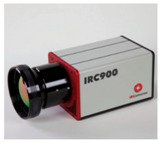 **IRC912 nBn (IRCameras)** sales@ircameras.com	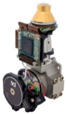 **Blackbird XBn HOT (SCD)** https://www.scd.co.il/products/
**Parameter**	**Performance**	
Array format	1280 × 1024	1280 × 1024
Pixel pitch	12 μm	10 μm
FPA temperature		150 K
FPA spectral range	1–5.3 μm	1–4.2 μm
*NEDT*	<35 mK	<25 mK at 70% well fill capacity
Well capacity	2 Me^−^	0.3 Me^−^, 0.2 Me^−^, 02 Me^−^, 3.5 Me^−^,
Maximum frame rate	119	60 Hz full frame
Optics	*f*/2.3, *f*/4	*f*/1.5, *f*/2, *f*/3.4, *f*/4
Cooler options	5 W	Rotary 0.5W cooler (standard)
Weight	<7 pounds	~720 gr.
Size	5.1″ × 5.8″ × 8″	Length (optical axis) −140 mm

**Table 7:** Performance of InAs/InAsSb T2SLs nBn detector FPAs ([172,211]).

	MWIR	LWIR
Format	640 × 512	640 × 512
Pixel size	24 × 24 µm^2^	24 × 24 µm^2^
Cut-off wavelength	5.37 μm (160 K)	12.5 µm (60 K)
Quantum efficiency	52% (150 K, no A/R coating)	30% (60 K)
Dark current	9.6 × 10^−5^ A/cm^2^ (−0.2 V, 157 K) (~4.5 × Rule’07)	2.6 × 10^−5^ A/cm^2^ (−0.05 V, 60 K)
Detectivity	3 × 10^11^ Jones (150 K, f/2 optics, 300 K background)	
NEDT	18.7 mK (160 K); 26.6 mK (170 K)	16.3 mK (62 K)
Operability	99.7% (160 K); 99.6% (170 K)	99.7% (60 K)

**Table 8:** LWIR device state-of-the-art. TRL-technology readiness level (TRL = 10 ideal maturity).

	Bolometer	HgCdTe	QWIP	T2 SLs InAs/GaSb	InAsSb-Based
**Maturity level**	TRL 9	TRL 9	TRL 9	TRL 7–8	TRL 7–8
**Status**	Applications where medium to low performance is required.	Application where high performance is required.	Commercial	Research and development.	Research and development.
**Operating temp.**	Un-cooled	Cooled	Cooled	Cooled	Cooled/TE cooled
**Manufacturability**	Excellent	Poor	Excellent	Very good	Very good
**Cost**	Low	High	Medium	Medium	Medium
**Prospect for large format**	Excellent	Very good	Excellent	Excellent	Excellent
**Availability of large substrate**	Excellent	Poor	Excellent	Very good	Very good
**Military applications**	Weapon sight, night vision goggles, missile seekers, small UAV sensors, unattended ground sensors.	Missile intercept, tactical ground and air born imaging, hyper spectral, missile seeker, missile tracking, space based sensing.	Being evaluated for some military applications and astronomy sensing.	Being evaluated for some military applications.	Being developed in university and evaluated industry research environment.
**Limitations**	Low sensitivity and long time constants, mechanical stability.	Performance susceptible to manufacturing variations. Difficult to extend to >14-µm cutoff.	Narrow bandwith and low sensitivity.	Requires a further investment and fundamental material breakthrough to mature.	Degradation of detector performance in a space environment due to the radiation-induced minority carrier lifetime degradation.
**Advantages**	Low cost and requires no active cooling, leverages standard Si manufacturing equipment.	Near theoretical performance, will remain material of choice for minimum of the next several years. High performance of substrate-removed arrays for space missions.	Low cost applications. Leverages commercial manufacturing processes. Very uniform material.	Theoretically better then HgCdTe, leverages commercial III–V fabrication techniques.	MWIR devices with significantly higher operating temperature than InSb.
